# Supplementary material for: Scalable phylogenetic profiling using MinHash uncovers likely eukaryotic sexual reproduction genes
Source: PLoS Comput Biol. 2020 Jul 22;16(7):e1007553. doi: 10.1371/journal.pcbi.1007553 (PMC7423146; doi:10.1371/journal.pcbi.1007553)
Supplement: Attachment — Submitted filename: Response-to-referees.pdf [file pcbi.1007553.s007.pdf]

Reviewer #1:

In my opinion phylogenetic profiles are one those methods that are intensively researched and developed by computational biologists but relatively poorly utilized by molecular biologist - some notable exceptions of course excluded. The reasons for this relative lack of utilization are many many fold, as also discussed in this manuscript. I sincerely hope that this manuscript will help to close this gap. I do have some comments perhaps not so much on the novel proposed methodology, as more on the way in which the results are introduced and contextualized.

RESPONSE: We thank the reviewer for their supportive and constructive assessment.

The introduction introduces the initial lack of genome diversity of eukaryotes as one of the issues in adopting phylogenetic profiles for eukaryotes, and then introduces OMA and the HOGs as a nice orthology database with "2000 cellular organisms". However it is not mentioned how many (and how diverse) eukaryotes OMA contains. It is my impression that the amount and diversity of eukaryotes in OMA is a minority in these 2000 organisms. I think it would be more transparent if the authors explicitly mention the amount (and "diversity") of eukaryotic organisms in OMA.

RESPONSE: We now provide the number and distribution of eukaryotic species in the introduction.

The introduction seems to suggest that phylogenetic profiles for many orthology databases are currently not offered. This is not completely true. The STRING-DB still allows phylogenetic profile searches not just on normalized "homology" (by default) but also on orthologs groups (although this option is somewhat hidden).

RESPONSE: We now cite STRING-DB and the phylogenetic profiling method they use to construct their profiles and detect coevolution. Also, we now mention the difference in approach ("Although this approach captures information on the distribution of extant distances, it does not reconstitute the evolutionary history of protein families and may lack information relative to duplication and loss events. Furthermore, as we show in the Methods section, the truncated Singular Value Decomposition approach does not scale well beyond a few genomes at a time.").

The introduction argues that the main reason that phylogenetic profiles are not used as much in eukaryotes as they could is speed of similarity computation. Perhaps this is indeed going to be a problem in the near future, but as general assertion I am not entirely convinced this statement is fully true. In our work we have so far been easily able on our local (admittedly beefy) workstations to successfully compute phylogenetic profile similarity for large eukaryotic data sets. Perhaps this point could be more made strongly if the present manuscript would include a smart implementation of jaccard of profile similarities on simple OMA/HOG presence/absence profile and show that indeed how/where the computational bottleneck is. (or perhaps the manuscript already present such an analysis and I missed it).

RESPONSE: We have added a figure (new Figure 6) illustrating the much better scaling properties of MinHash based data structures. This shows the utility of our approach in the current research context where the number of genomes is growing exponentially.

I think that the orthology database and the method of phylogenetic profile searching are not strictly necessarily connected. The introduced MinHash search method seems to need an orthology that allows a species tree to be annotated with duplications and losses. Such data are available elsewhere. Most easily they should be extractable from the PANTHER database. But also EGGNOG is hierarchical and they could perhaps also be retrieved from numerous ENSEMBL compara genome subsets. I think it would strengthen the message of applicability of this method if it would be applied to other orthology datasets.

RESPONSE: The methods section has been reworked to more clearly describe the different steps of the pipeline as well as the inputs and outputs of each step. We now explicitly mention the possibility of using other sources of orthology data as input using other databases, for instance by converting gene trees to OrthoXML using tools such as ETE ("pyHam can also be used to infer enhanced phylogenies for other datasets available in OrthoXML format such as ENSEMBL (Zerbino et al. 2018) or with data generated from phylogenetic trees such as those found in PANTHER (Mi et al. 2017) through the use of the function etree2orthoxml() in the tree analysis package ETE3 (Huerta-Cepas, Serra, and Bork 2016).") We thank the reviewer for noting this important point and now we think our method applicability is clear and accessible to the reader.

For evaluating potential novel connections to kinetochore it appears the proteins detailed in Table 2 exemplify another problem with finding wide-spread utilization of phylogenetic profiles by molecular biologists. So I reached out via the bioRxiv version of this article to a molecular biologist somewhat familiar with the kinetochore. It seems that the co-evolution of APC12 with CDC26 is a spurious orthology/identifier problem as CDC26 is a synonym of APC12 and reference [29] used as evidence still using the old nomenclature for APC12. The co-evolution of KNL1 with TACC3 is asserted to bind to the kinetochore but insofar as they understand the literature this is not the case and reference [30] is also not showing that. Some very indirect linkage of TACC3 to kinetochore function is known to the extent that TACC3 is microtubule-associated and seems to be stabilizing the spindle, but that does not qualify as being part of the well defined set of complexes that make up the kinetochore. The other links were seen as not specific enough to be relevant for a molecular biologists but I guess this dismissal by experimentalist is more a Gene Ontology versus real biology problem than something inherent to phylogenetic profiles.

RESPONSE: Thank you again for your detailed critique and consideration of our conclusions regarding the molecular biology of the kinetochore. Indeed we overlooked the equivalence between cdc26 and apc12 and some of the returned results may not be directly physically bound to the kinetochore complex. We have revised this section to make this clear.

In the discussion, potential expansions of this method to account for neofunctionalization after duplications are mentioned. This is indeed one of those cool and difficult things on thinking about phylogenetic profiles and the evolution of function. When discussing this possible extension of the method it could be worth to add another citation to an already extensive citation list. Because this paper: doi: 10.1016/j.celrep.2015.01.025 from Tobias Meyer already makes phylogenetic profile searches where the neofunctionalization is explicitly taken into account.

RESPONSE: Yes, this method does address this concept explicitly. We have added the citation and mention this study in the text.

Reviewer #2:

Dear Authors. Please see some of my comments on the annotated pdf attached. Although you have presented a study with potential relevance in bioinformatics and computational biology, I consider that the ms needs heavy revisions to accomplish the criteria for publication in the Journal.

RESPONSE: We thank the reviewer for the detailed feedback, which greatly helped us to improve the organisation and clarity of the manuscript. Throughout, we have reformulated, shortened and also deleted much of the redundant expositions, mainly in the results section, as suggested. We also added citations as per your suggestions.

The methods section has now been rewritten to make the steps of the pipeline, as well as the input and output of each step, more explicit.

As per your suggestions we have moved material explaining the construction of the pipeline from the results sections to the introduction and shortened it, removing redundancy.

Font sizes have been corrected throughout the manuscript for consistency.

The figure showing the ROC characteristics of the various profiling methods has been revised to include a clearer legend.

Figure legends and table descriptions have also been shortened throughout the manuscript.
