# Supplementary material for: Scalable phylogenetic profiling using MinHash uncovers likely eukaryotic sexual reproduction genes
Source: PLoS Comput Biol. 2020 Jul 22;16(7):e1007553. doi: 10.1371/journal.pcbi.1007553 (PMC7423146; doi:10.1371/journal.pcbi.1007553)
Supplement: Attachment — Submitted filename: PCOMPBIOL-D-19-01799_R1_reviewer_P-T.pdf [file pcbi.1007553.s008.pdf]

Revision 1

PLOS Computational Biology

# Scalable Phylogenetic Profiling using MinHash Uncovers Likely Eukaryotic Sexual Reproduction Genes

--Manuscript Draft--

| | |
|---|---|
| **Manuscript Number:** | PCOMPBIOL-D-19-01799R1 |
| **Full Title:** | Scalable Phylogenetic Profiling using MinHash Uncovers Likely Eukaryotic Sexual Reproduction Genes |
| **Short Title:** | Scalable Phylogenetic Profiling Uncovers Likely Eukaryotic Sexual Reproduction Genes |
| **Article Type:** | Research Article |
| **Keywords:** | phylogenetic profiling; co-evolution; phylogenetics; orthologs; function prediction; sexual reproduction; kinetochore; network; interaction; complex |
| **Abstract:** | Phylogenetic profiling is a computational method to predict genes involved in the same biological process by identifying protein families which tend to be jointly lost or retained across the tree of life. Phylogenetic profiling has customarily been more widely used with prokaryotes than eukaryotes, because the method is thought to require many diverse genomes. There are now many eukaryotic genomes available, but these are considerably larger, and typical phylogenetic profiling methods require at least quadratic time as a function of the number of genes. We introduce a fast, scalable phylogenetic profiling approach entitled HogProf, which leverages hierarchical orthologous groups for the construction of large profiles and locality-sensitive hashing for efficient retrieval of similar profiles. We show that the approach outperforms Enhanced Phylogenetic Tree, a phylogeny-based method, and use the tool to reconstruct networks and query for interactors of the kinetochore complex as well as conserved proteins involved in sexual reproduction: Hap2, Spo11 and Gex1. HogProf enables large-scale phylogenetic profiling across the three domains of life, and will be useful to predict biological pathways among the hundreds of thousands of eukaryotic species that will become available in the coming few years. HogProf is available at https://github.com/DessimozLab/HogProf . |
| **Additional Information:** | |
| **Question** | **Response** |
| **Financial Disclosure**<br><br>Enter a financial disclosure statement that describes the sources of funding for the work included in this submission. Review the submission guidelines for detailed requirements. View published research articles from *PLOS Computational Biology* for specific examples.<br><br>This statement is required for submission and **will appear in the published article** if the submission is accepted. Please make sure it is accurate. | We thank Monique Zahn for helpful feedback on the manuscript. This work was funded by a grant by the Novartis Foundation for Medical-Biological Research (#17B111 to CD), by the Swiss National Science Foundation (Grant 183723 to CD), by the Swiss Leading House for the Latin American Region (to CD and PSA), and by the Agencia Nacional de Promoción Científica y Tecnológica (PICT-2017-0854 to PSA).<br><br>* http://www.stiftungmedbiol.novartis.com<br>* https://cls.unisg.ch/de/forschung/leading-house/funding-instruments/2019-seed-money-grants<br>* http://www.snf.ch<br>* https://www.argentina.gob.ar/ciencia/agencia<br><br>The funders had no role in study design, data collection and analysis, decision to publish, or preparation of the manuscript. |

*Powered by Editorial Manager® and ProduXion Manager® from Aries Systems Corporation*

| | |
|---|---|
| **Unfunded studies**<br>Enter: *The author(s) received no specific funding for this work.*<br><br>**Funded studies**<br>Enter a statement with the following details:<br>• Initials of the authors who received each award<br>• Grant numbers awarded to each author<br>• The full name of each funder<br>• URL of each funder website<br>• Did the sponsors or funders play any role in the study design, data collection and analysis, decision to publish, or preparation of the manuscript?<br>• **NO** - Include this sentence at the end of your statement: *The funders had no role in study design, data collection and analysis, decision to publish, or preparation of the manuscript.*<br>• **YES** - Specify the role(s) played.<br><br>* typeset | |
| **Competing Interests**<br><br>Use the instructions below to enter a competing interest statement for this submission. On behalf of all authors, disclose any competing interests that could be perceived to bias this work—acknowledging all financial support and any other relevant financial or non-financial competing interests.<br><br>This statement **will appear in the published article** if the submission is accepted. Please make sure it is accurate. View published research articles from *PLOS Computational Biology* for specific examples. | The authors have declared that no competing interests exist. |

| | |
|---|---|
| **NO authors have competing interests**<br><br>Enter: *The authors have declared that no competing interests exist.*<br><br>**Authors with competing interests**<br><br>Enter competing interest details beginning with this statement:<br><br>*I have read the journal's policy and the authors of this manuscript have the following competing interests: [insert competing interests here]*<br><br>* typeset | |
| **Data Availability**<br><br>Authors are required to make all data underlying the findings described fully available, without restriction, and from the time of publication. PLOS allows rare exceptions to address legal and ethical concerns. See the PLOS Data Policy and FAQ for detailed information.<br><br>A Data Availability Statement describing where the data can be found is required at submission. Your answers to this question constitute the Data Availability Statement and **will be published in the article**, if accepted.<br><br>**Important:** Stating 'data available on request from the author' is not sufficient. If your data are only available upon request, select 'No' for the first question and explain your exceptional situation in the text box.<br><br>Do the authors confirm that all data underlying the findings described in their manuscript are fully available without restriction? | Yes - all data are fully available without restriction |
| **Describe where the data may be found in full sentences. If you are copying our sample text, replace any instances of XXX with the appropriate details.** | The data is available as supplementary materials. The code is released on GitHub under an open source license. |

| | |
|---|---|
| • If the data are **held or will be held in a public repository**, include URLs, accession numbers or DOIs. If this information will only be available after acceptance, indicate this by ticking the box below. For example: *All XXX files are available from the XXX database (accession number(s) XXX, XXX.*).<br>• If the data are all contained **within the manuscript and/or Supporting Information files**, enter the following: *All relevant data are within the manuscript and its Supporting Information files.*<br>• If neither of these applies but you are able to provide **details of access elsewhere**, with or without limitations, please do so. For example:<br><br>*Data cannot be shared publicly because of [XXX]. Data are available from the XXX Institutional Data Access / Ethics Committee (contact via XXX) for researchers who meet the criteria for access to confidential data.*<br><br>*The data underlying the results presented in the study are available from (include the name of the third party and contact information or URL).*<br>• This text is appropriate if the data are owned by a third party and authors do not have permission to share the data.<br><br>* typeset | |
| Additional data availability information: | |

*Reviewer #1:*

*In my opinion phylogenetic profiles are one those methods that are intensively researched and developed by computational biologists but relatively poorly utilized by molecular biologist - some notable exceptions of course excluded. The reasons for this relative lack of utilization are many many fold, as also discussed in this manuscript. I sincerely hope that this manuscript will help to close this gap. I do have some comments perhaps not so much on the novel proposed methodology, as more on the way in which the results are introduced and contextualized.*

**RESPONSE: We thank the reviewer for their supportive and constructive assessment.**

*The introduction introduces the initial lack of genome diversity of eukaryotes as one of the issues in adopting phylogenetic profiles for eukaryotes, and then introduces OMA and the HOGs as a nice orthology database with "2000 cellular organisms". However it is not mentioned how many (and how diverse) eukaryotes OMA contains. It is my impression that the amount and diversity of eukaryotes in OMA is a minority in these 2000 organisms. I think it would be more transparent if the authors explicitly mention the amount (and "diversity") of eukaryotic organisms in OMA.*

**RESPONSE: We now provide the number and distribution of eukaryotic species in the introduction.**

*The introduction seems to suggest that phylogenetic profiles for many orthology databases are currently not offered. This is not completely true. The STRING-DB still allows phylogenetic profile searches not just on normalized "homology" (by default) but also on orthologs groups (although this option is somewhat hidden).*

**RESPONSE: We now cite STRING-DB and the phylogenetic profiling method they use to construct their profiles and detect coevolution. Also, we now mention the difference in approach ("Although this approach captures information on the distribution of extant distances, it does not reconstitute the evolutionary history of protein families and may lack information relative to duplication and loss events. Furthermore, as we show in the Methods section, the truncated Singular Value Decomposition approach does not scale well beyond a few genomes at a time.").**

*The introduction argues that the main reason that phylogenetic profiles are not used as much in eukaryotes as they could is speed of similarity computation. Perhaps this is indeed going to be a problem in the near future, but as general assertion I am not entirely convinced this statement is fully true. In our work we have so far been easily able on our local (admittedly beefy) workstations to successfully compute phylogenetic profile similarity for large eukaryotic data sets. Perhaps this point could be more made strongly if the present manuscript would include a smart implementation of jaccard of profile similarities on simple OMA/HOG presence/absence profile and show that indeed how/where the computational bottleneck is. (or perhaps the manuscript already present such an analysis and I missed it).*

**RESPONSE: We have added a figure (new Figure 6) illustrating the much better scaling properties of MinHash based data structures. This shows the utility of our approach in the current research context where the number of genomes is growing exponentially.**

*I think that the orthology database and the method of phylogenetic profile searching are not strictly necessarily connected. The introduced MinHash search method seems to need an orthology that allows a species tree to be annotated with duplications and losses. Such data are available elsewhere. Most easily they should be extractable from the PANTHER database. But also EGGNOG is hierarchical and they could perhaps also be retrieved from numerous ENSEMBL compara genome subsets. I think it would strengthen the message of applicability of this method if it would be applied to other orthology datasets.*

**RESPONSE: The methods section has been reworked to more clearly describe the different steps of the pipeline as well as the inputs and outputs of each step. We now explicitly mention the possibility of using other sources of orthology data as input using other databases, for instance by converting gene trees to OrthoXML using tools such as ETE ("pyHam can also be used to infer enhanced phylogenies for other datasets available in OrthoXML format such as ENSEMBL (Zerbino et al. 2018) or with data generated from phylogenetic trees such as those found in PANTHER (Mi et al. 2017) through the use of the function etree2orthoxml() in the tree analysis package ETE3 (Huerta-Cepas, Serra, and Bork 2016).") We thank the reviewer for noting this important point and now we think our method applicability is clear and accessible to the reader.**

*For evaluating potential novel connections to kinetochore it appears the proteins detailed in Table 2 exemplify another problem with finding wide-spread utilization of phylogenetic profiles by molecular biologists. So I reached out via the bioRxiv version of this article to a molecular biologist somewhat familiar with the kinetochore. It seems that the co-evolution of APC12 with CDC26 is a spurious orthology/identifier problem as CDC26 is a synonym of APC12 and reference [29] used as evidence still using the old nomenclature for APC12. The co-evolution of KNL1 with TACC3 is asserted to bind to the kinetochore but insofar as they understand the literature this is not the case and reference [30] is also not showing that. Some very indirect linkage of TACC3 to kinetochore function is known to the extent that TACC3 is microtubule-associated and seems to be stabilizing the spindle, but that does not qualify as being part of the well defined set of complexes that make up the kinetochore. The other links were seen as not specific enough to be relevant for a molecular biologists but I guess this dismissal by experimentalist is more a Gene Ontology versus real biology problem than something inherent to phylogenetic profiles.*

**RESPONSE: Thank you again for your detailed critique and consideration of our conclusions regarding the molecular biology of the kinetochore. Indeed we overlooked the equivalence between cdc26 and apc12 and some of the returned results may not be directly physically bound to the kinetochore complex. We have revised this section to make this clear.**

*In the discussion, potential expansions of this method to account for neofunctionalization after duplications are mentioned. This is indeed one of those cool and difficult things on thinking about phylogenetic profiles and the evolution of function. When discussing this possible extension of the method it could be worth to add another citation to an already extensive citation list. Because this paper: doi: 10.1016/j.celrep.2015.01.025 from Tobias Meyer already makes phylogenetic profile searches where the neofunctionalization is explicitly taken into account.*

**RESPONSE: Yes, this method does address this concept explicitly. We have added the citation and mention this study in the text.**

Reviewer #2:

Dear Authors. Please see some of my comments on the annotated pdf attached. Although you have presented a study with potential relevance in bioinformatics and computational biology, I consider that the ms needs heavy revisions to accomplish the criteria for publication in the Journal.

**RESPONSE: We thank the reviewer for the detailed feedback, which greatly helped us to improve the organisation and clarity of the manuscript. Throughout, we have reformulated, shortened and also deleted much of the redundant expositions, mainly in the results section, as suggested. We also added citations as per your suggestions.**

**The methods section has now been rewritten to make the steps of the pipeline, as well as the input and output of each step, more explicit.**

**As per your suggestions we have moved material explaining the construction of the pipeline from the results sections to the introduction and shortened it, removing redundancy.**

**Font sizes have been corrected throughout the manuscript for consistency.**

**The figure showing the ROC characteristics of the various profiling methods has been revised to include a clearer legend.**

**Figure legends and table descriptions have also been shortened throughout the manuscript.**

Manuscript Click here to access/download;Manuscript;resubmission.docx



# Scalable Phylogenetic Profiling using MinHash Uncovers Likely Eukaryotic Sexual Reproduction Genes

**David Moi[1,2,3,*], Laurent Kilchoer[1,2,3], Pablo S. Aguilar[4,5] and Christophe Dessimoz[1,2,3,6,7,*]**

[1]Department of Computational Biology, University of Lausanne, Switzerland; [2]Center for Integrative Genomics, University of Lausanne, Switzerland; [3]SIB Swiss Institute of Bioinformatics, Lausanne, Switzerland; [4]Instituto de Investigaciones Biotecnologicas (IIBIO), Universidad Nacional de San Martín Buenos Aires, Argentina; [5]Instituto de Fisiología, Biología Molecular y Neurociencias (IFIBYNE-CONICET), [6]Department of Genetics, Evolution, and Environment, University College London, UK; [7]Department of Computer Science, University College London, UK.

*Corresponding authors: david.moi@unil.ch and christophe.dessimoz@unil.ch

## Abstract

Phylogenetic profiling is a computational method to predict genes involved in the same biological process by identifying protein families which tend to be jointly lost or retained across the tree of life. Phylogenetic profiling has customarily been more widely used with prokaryotes than eukaryotes, because the method is thought to require many diverse genomes. There are now many eukaryotic genomes available, but these are considerably larger, and typical phylogenetic profiling methods require at least quadratic time as a function of the number of genes. We introduce a fast, scalable phylogenetic profiling approach entitled HogProf, which

leverages hierarchical orthologous groups for the construction of large profiles and locality-sensitive hashing for efficient retrieval of similar profiles. We show that the approach outperforms Enhanced Phylogenetic Tree, a phylogeny-based method, and use the tool to reconstruct networks and query for interactors of the kinetochore complex as well as conserved proteins involved in sexual reproduction: Hap2, Spo11 and Gex1. HogProf enables large-scale phylogenetic profiling across the three domains of life, and will be useful to predict biological pathways among the hundreds of thousands of eukaryotic species that will become available in the coming few years. HogProf is available at https://github.com/DessimozLab/HogProf.

## Introduction

The NCBI Sequence Read Archive (SRA) contains 1.6x$10^{16}$ nucleotide bases of data and the quantity of sequenced organisms keeps growing exponentially. To make sense of all of this new genomic information, annotation pipelines need to overcome speed and accuracy barriers. Even in a well-studied model organism such as *Arabidopsis thaliana*, nearly a quarter of all genes are not annotated with an informative gene ontology term (Skunca, Altenhoff, and Dessimoz 2012; "TAIR - Portals - Genome Snapshot" n.d.). One way to infer the function of a gene product is to analyse the biological network it is involved in. Using guilt by association strategies it is possible to infer function based on physical or regulatory interactors. Unfortunately, biological network inference

is mostly limited to model organisms and genome scale data is only available through the use of noisy high-throughput experiments.

To ascribe biological functions to these new sequences, most of which originate from non-model organisms, computational methods are essential (reviewed in Cozzetto and Jones 2017). Among the computational function prediction techniques that leverage the existing body of experimental data, one important but still underutilised approach in eukaryotes is *phylogenetic profiling (Pellegrini et al. 1999)*: positively correlated patterns of gene gains and losses across the tree of life are suggestive of genes involved in the same biological pathways.

Phylogenetic profiling has been more commonly performed on prokaryotic genomes than on eukaryotic ones. Perhaps due to the relative paucity of eukaryotic genomes in the 2000s, earlier benchmarking studies observed poorer performance in retrieving known interactions with eukaryotes than with Prokaryotes (Snitkin et al. 2006; Jothi, Przytycka, and Aravind 2007; Ruano-Rubio, Poch, and Thompson 2009). The situation today is considerably different; the GOLD database (Mukherjee et al. 2017) tracks over 6000 eukaryotic genomes. Multiple successful applications of phylogenetic profiling in eukaryotes have been published in recent years. For example, they have been used to infer small RNA pathway genes (Tabach et al. 2013), the kinetochore network (van Hooff et al. 2017), ciliary genes (Nevers et al. 2017), or homologous recombination repair genes (Sherill-Rofe et al. 2019).

Large-scale phylogenetic profiling with complex eukaryotic genomes is computationally challenging since most state-of-the-art phylogenetic profiling methods typically scale at least quadratically with the number of gene families and linearly with the number of genomes. As a

result, most mainstream phylogenomic databases, such as Ensembl (Zerbino et al. 2018), EggNOG (Huerta-Cepas et al. 2016), OrthoDB (Zdobnov et al. 2017), or OMA (Altenhoff et al. 2018) do not provide phylogenetic profiles. One available resource is STRING (Szklarczyk et al. 2017), a protein interaction focused database which integrates multiple channels of evidence to support each interaction. The links between profiles STRING offers are obtained using SVD-phy (Franceschini et al. 2016) which represents profiles as bit-score distances between all proteins present in a given proteome and their closest homologues in all of the genomes included in the analysis. Dimensionality reduction is applied to the matrix to remove signal coming from the species tree and the profiles are clustered to infer interactions. In STRING, this is implemented with their set of 2031 organisms for which profile distance matrices are precalculated and incorporated into their network inference pipeline. Although this approach captures information on the distribution of extant distances, it does not reconstitute the evolutionary history of protein families and may lack information relative to duplication and loss events. Furthermore, as we show in the *Methods section*, the truncated Singular Value Decomposition approach does not scale well beyond a few genomes at a time.

To construct profiles representing groups of homologues, some pipelines resort to all-vs-all sequence similarity searches to derive orthologous groups and only count binary presence or absence of a member of each group in a limited number of genomes (Ta, Koskinen, and Holm 2011; Kensche et al. 2008) or forgo this step altogether and ignore the evolutionary history of each protein family, relying instead on co-occurrence in extant genomes (Niu et al. 2017). Other tree-based methods infer the underlying evolutionary history from the presence of extant homologues (Li et al. 2014).

Here, we introduce a scalable approach which combines the efficient generation of phylogeny-aware profiles from hierarchical orthologous groups with ultrafast retrieval of similar profiles using locality sensitive hashing. A scalable phylogenetic profiling method using locality-sensitive hashing and hierarchical orthologous groups

Most phylogenetic profiling methods consist of two steps: creating a profile for each homologous or orthologous group, and comparing profiles. When they were first implemented, profiles were constructed as binary vectors of presence and absence across species (Pellegrini et al. 1999). Since then, variants have been proposed, which take continuous values (van Hooff et al. 2017)—such as alignment scores with the gene of a reference species (Sherill-Rofe et al. 2019)—or which count the number of paralogs present in each species. Yet other variants convey the number of events on branches of the species tree (Ruano-Rubio, Poch, and Thompson 2009).

In our pipeline, we leveraged the already existing OMA orthology inference algorithm to provide the input data to create our profiles. The OMA database describes the orthology relationships among all protein coding genes of currently 2288 cellular organisms (1674 bacteria, 152 archaea, and 462 eukaryotes). Within eukaryotes, OMA includes 188 animals, 135 fungi, 57 plants, and 82 protists and has been benchmarked and integrated with other proteomic and genomic resources (Altenhoff et al. 2018). One core object of this database is the Hierarchical Orthologous Group (HOG) (Altenhoff et al. 2013). Each HOG contains all of the descendants of a single ancestor gene. When a gene is duplicated during its evolution, the paralogous genes and the descendants of the orthologue are contained in separate subhogs which describe their lineage back to their single ancestor gene (hence the hierarchical descriptor).

We captured the evolutionary history of each HOG in enhanced phylogenies and encoded them in probabilistic data structures (Fig. 1). These are used to compile searchable databases to allow for the retrieval of coevolving HOGs with similar evolutionary histories and compare the similarity of two HOGs. The two major components of the pipeline that are responsible for constructing the enhanced phylogenies and calculating probabilistic data structures to represent them are pyHam (Train et al. 2018) and Datasketch ("Datasketch: Big Data Looks Small — Datasketch 1.0.0 Documentation" n.d.), respectively. Further details on the implementation are provided in the Methods section. The combination of these two tools now allows for the main innovation of our pipeline: the efficient exploration and clustering of profiles to study known and novel biological networks.

Currently, existing profiling pipelines are limited with respect to the computational power required to cluster profiles using their respective distance metrics. Due to this bottleneck, profiling efforts are typically focused on reconstructing pathways with known interactors using existing annotations and evidence rather than being used as an exploratory tool to search for new interactors and reconstituting completely unknown networks.

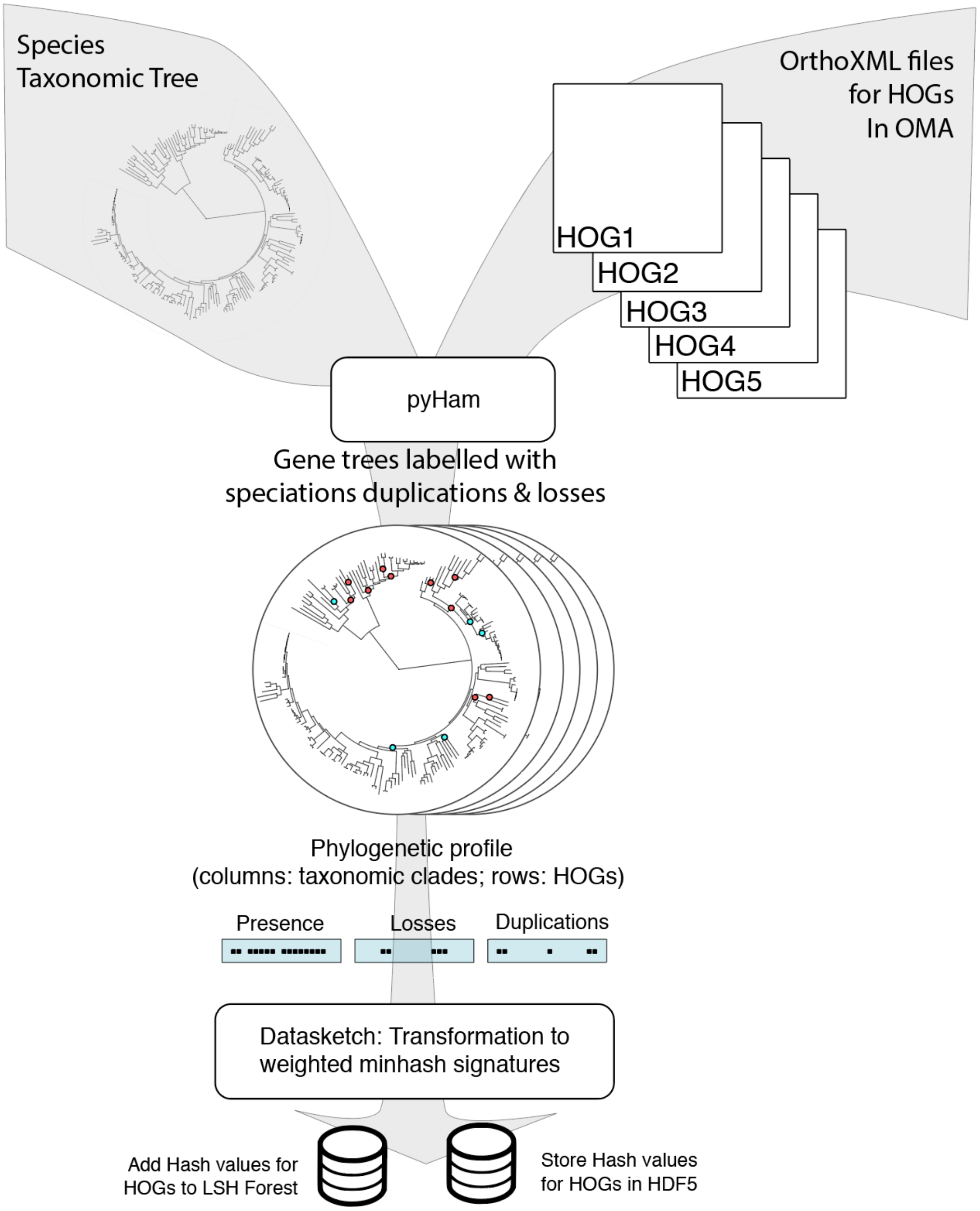


**Fig. 1. Diagram summarizing the different steps of the pipeline to generate the LSH Forest and hash signatures for each HOG.** The labelled phylogenetic trees generated by pyHam are converted into phylogenetic profiles and used to generate a weighted MinHash signature with Datasketch. The hash signatures are inserted into the LSH Forest and stored in an HDF5 file.

The tool we have ~~created~~ developed leverages the properties of MinHash signatures to allow for the selection of clade subsets and for clade weightings in the construction of profiles and make it possible to build profiles with the complete set of genomes contained in OMA. We show that the method outperforms other phylogeny-based methods (Ta, Koskinen, and Holm 2011; Glazko and Mushegian 2004; Ranea et al. 2007), and illustrate its usefulness by retrieving biologically relevant results for several genes of interest. Because the method is unaffected by the number of genomes included and scales logarithmically with the number of hierarchical orthologous groups added, it will efficiently perform with the exponentially growing number of genomes as they become available.

The code used to generate the results in this manuscript are available at https://github.com/DessimozLab/HogProf.

## Results

In the following sections we first compare our profiling distance metric against other profile distances in order to characterize the Jaccard hash estimation's precision and recall characteristics. Following this quantification, we show our pipeline's capacity in reconstituting a well known interaction network as well as augmenting it with more putative interactors using its search functionality. Finally, to illustrate a typical use case of our tool, we explore a poorly characterized network.

**Accuracy of predicted phylogenetic profiles in an empirical benchmark**

We compared the performance of our profiling metric to existing profile distances using benchmarking data available in Ta *et al.* (2011). In that benchmark, the true positive protein-protein interactions (PPIs) were constructed using data available from CORUM (Giurgiu et al. 2018) and the MIPS (Mewes et al. 2004) databases for the human and yeast interaction datasets. True negatives were constructed by mixing proteins known to be involved in different complexes. The dataset is balanced with 50% positive and 50% negative samples. Using their Uniprot identifiers, these interaction pairs were mapped to their respective HOGs and their profiles were compared using the hash based Jaccard score estimate. The comparison below shows HogProf alongside other profiling distance metrics that are considerably more computationally intensive, including the Enhanced Phylogenetic Tree (EPT) metric shown in Ta *et al.* (2011). Yet, our approach outperformed these previous methods, yielding the highest Area Under the Curve for both yeast and human datasets (Figure 2, Table 1).

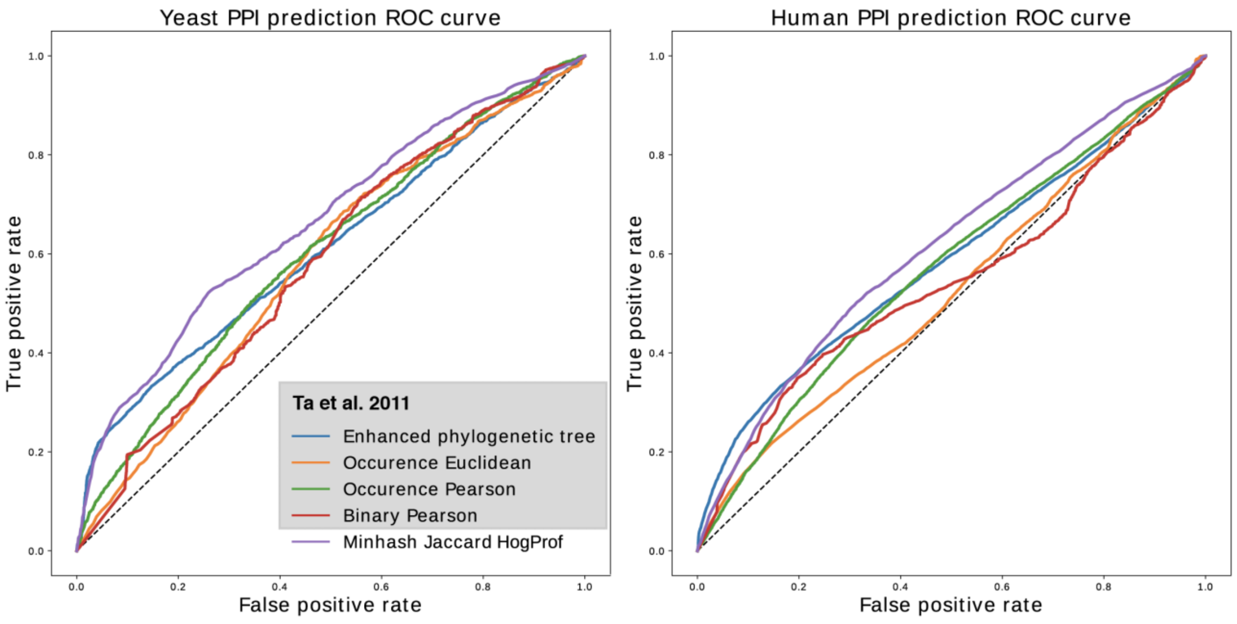


**Fig. 2. ROC curves for all profiling methods**. **a.** Yeast protein-protein interactions. Our method (MinHash Jaccard HogProf), performs best overall, but when high precision is required, Enhanced phylogenetic Tree (Ta, Koskinen, and Holm 2011) is still slightly more accurate. **b.** Human protein-protein interactions. Jaccard Hash HogProf performs better than all metrics overall but again, when high precision is required, EPT score is still slightly more accurate. Binary Pearson refers to a distance using binary vectors and Pearson correlation described in (Glazko and Mushegian 2004). Occurence Euclidean and Occurence Pearson refer to the occurence profiles with Euclidean distance and Pearson correlation as described in (Ranea et al. 2007).

**Table 1. AUC values for Profiling distance metrics.**

| Metric | AUC Yeast | AUC Human |
|---|---|---|
| Jaccard Hash | 0.6634 | 0.6155 |
| EPT | 0.6104 | 0.5875 |

| | | |
|---|---|---|
| BIN PS | 0.5840 | 0.5463 |
| OCC ED | 0.5829 | 0.5268 |
| OCC PS | 0.6028 | 0.5714 |

**Recovery of a canonical network: the kinetochore network**

To further validate our profiling approach on a known biological network, we used our pipeline to replicate previous work shown in van Hooff et al. (2017). Their analysis focuses on the evolutionary dynamics of the kinetochore complex, a microtubule organizing structure that was present in the last eukaryotic common ancestor (LECA) and has undergone many modifications throughout evolution in each eukaryotic clade where it is found. Its modular organization has allowed for clade-specific additions or deletions of modules to the core complex which remains relatively stable. This modular organisation and clade-specific emergence of certain parts of the complex make it an ideal target for phylogenetic profiling analysis.

We show that our MinHash signature comparisons are also capable of recovering the kinetochore complex organisation. After considering just the HOGs for the families used in van Hooff et al. (van Hooff et al. 2017), we augmented their set of profiles using LSH Forest (Bawa, Condie, and Ganesan 2005) to retrieve interactors that may also be involved in the kinetochore (and the also included anaphase promoting complex (APC)) networks which have not been cataloged by these authors. Using the Gene Ontology (GO) terms (Ashburner et al. 2000) of all proteins returned in our searches for novel interactors, we were able to identify proteins with specific functions we would expect to be related to our network of interest.

In their work, van Hooff et al. (van Hooff et al. 2017) used pairwise Pearson correlation coefficients between the presence and absence vectors of the various kinetochore components to recompose the organisation of the complex. Their profiles were constructed using the proteomes of a manually selected set of 90 organisms with manually curated profiles corresponding to each component of the complex. After establishing a distance kernel, they clustered the profiles and were able to recover known sub-components of the complex using just evolutionary information. Using our hash-based Jaccard distance metric in an all-vs-all comparison between the HOGs corresponding to each of these protein families, we were also able to recover the main modules of the kinetochore complex with a similar organisation to the one defined by van Hooff et al. The color clustering in Figure 3 corresponds to their original manual definition of these different subcomplex modules. We observe that the distance matrices generated by each profiling approach are correlated (with Spearman correlation of 0.268 ($p < 1e-100$) and Pearson correlation of 0.364 ($p < 1e-100$) ) and are recovering similar evolutionary signals despite their construction using different methods.

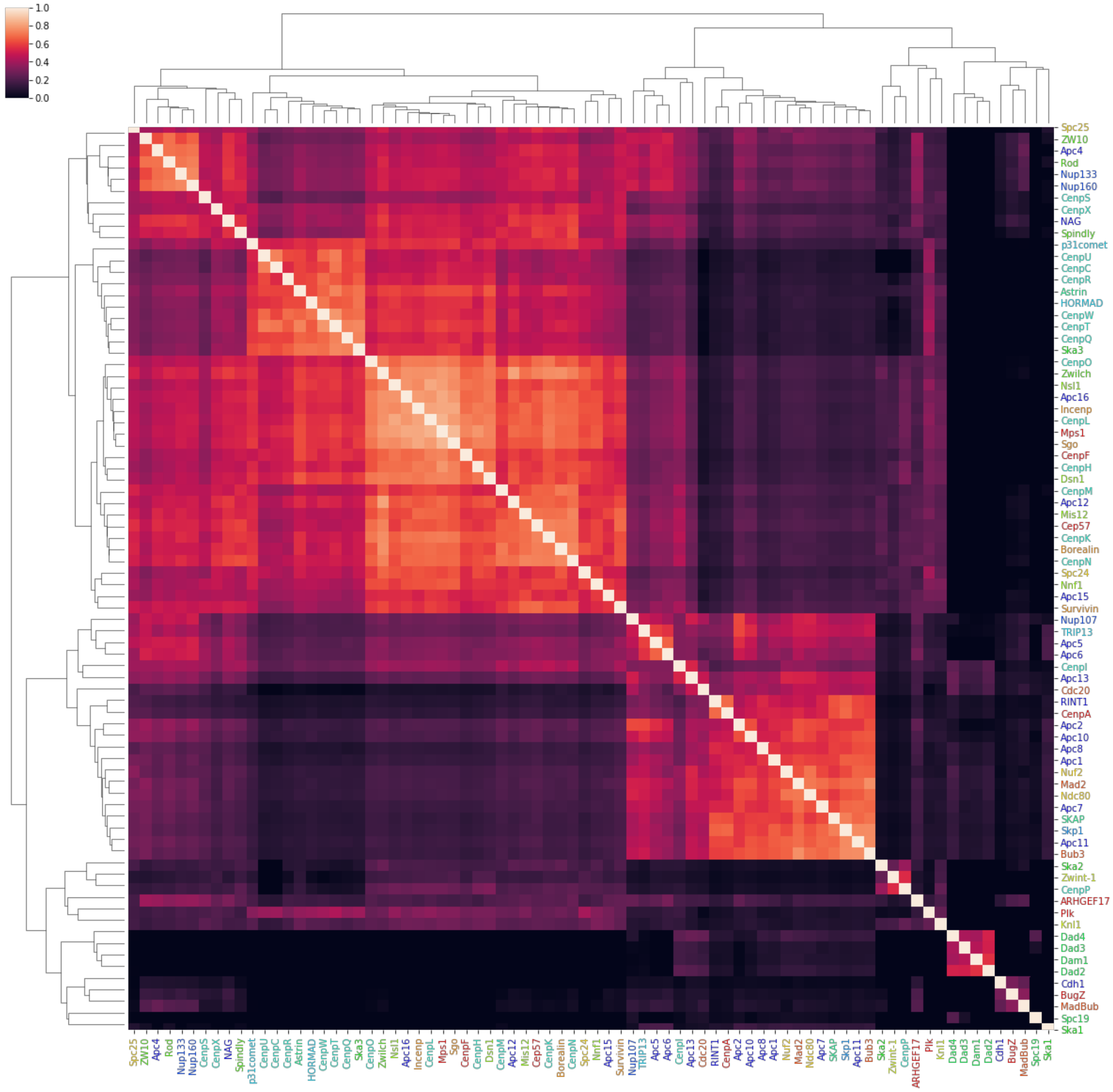


**Fig. 3. Recovery of kinetochore and APC complexes.** After mapping each of the protein families presented in Van Hooff et al. (van Hooff et al. 2017) to their corresponding HOG, a distance matrix was constructed by comparing the Jaccard hash distance between profiles using HogProf. Name colors in the rows and columns of the matrix correspond to the kinetochore

and APC subcomplex components as defined manually using literature sources (van Hooff et al. 2017).

The All-vs-All comparison of the profiles revealed several well defined clusters in both studies including the Dam-Dad-Spc19 and CenP subcomplexes. Unlike the Van Hoof er al. approach, HogProf profiles were constructed alongside all other HOGs in OMA and were not curated before being compared. With only the initial information of which proteins were in the complex, we mapped them to their corresponding OMA HOGs and, with this example, demonstrated the ability to reconstruct any network of interest or construct putative networks using the search functionality of our pipeline with minimal computing time. It should be noted that the quality of the OMA HOGs used to construct the enhanced phylogenies and hash signatures directly influences our ability to recover complex organisation.

To illustrate the utility of the search functionality of our tool, we used the profiles known to be associated with the kinetochore complex to search for other interactors. All HOGs corresponding to the protein families used to analyse the kinetochore evolutionary dynamics in van Hooff et al. (van Hooff et al. 2017) were used as queries against an LSH Forest containing all HOGs in OMA. By performing an all-vs-all comparison of the minhash signatures of the queries and returned results, a Jaccard distance matrix was generated showing potential functional modules associated with each known component of the kinetochore and APC complexes.

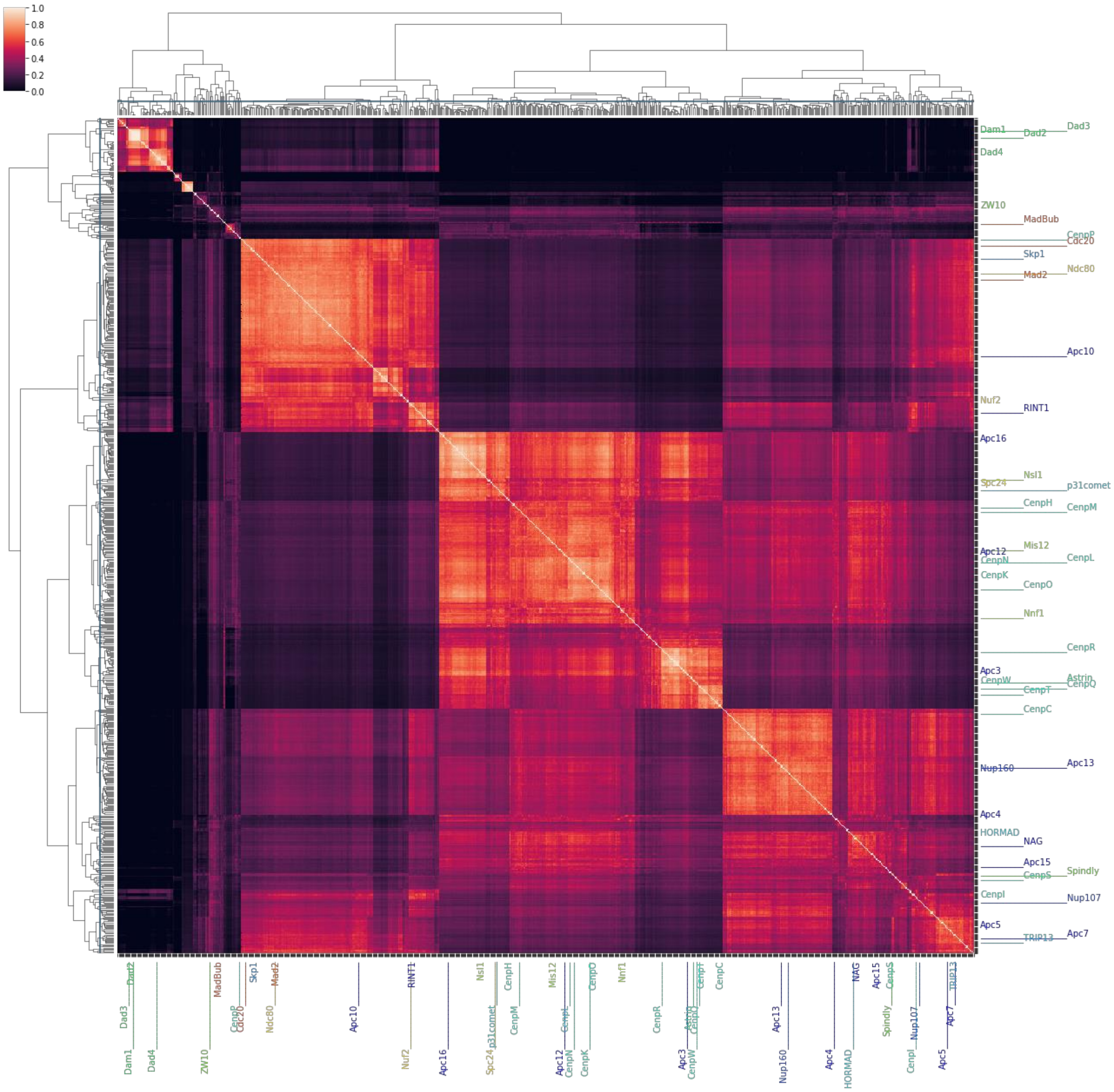


**Fig. 4. Putative novel components of the kinetochore and APC complexes.** The profiles associated with all HOGs mapping to known kinetochore components shown in Figure 3 were used to search the LSH Forest and retrieve the top 10 closest coevolving HOGs resulting in a list of 871 HOGs including the queries from the original complexes. The Jaccard distance matrix is shown between the hash signatures of all query and result HOGs. UPGMA clustering was

applied to the distance matrix rows and columns. Labelled rows and columns correspond to profiles from the starting kinetochore dataset (van Hooff et al. 2017). A cutoff hierarchical clustering distance of 1.3 was manually chosen (blue lines) to limit the maximum cluster size to less than 50 HOGs. This cutoff resulted in a total of 142 clusters of HOGs used for GO enrichment to identify functional modules. The coloring of the protein family names to the right and below the matrix is identical to the complex related coloring shown in Figure 3.

To verify that the results returned by our search were not spurious, we performed GO enrichment analysis of the returned HOGs that were not part of the original set of queries but appeared to be coevolving closely with known kinetochore components. Given the incomplete nature of GO annotations ("open world assumption", Dessimoz, Škunca, and Thomas 2013), many of these proteins may actually be involved in the kinetochore interaction network but this biological function could be still undiscovered. However, even with this limitation, salient annotations relevant to the kinetochore network were returned in the search results (Table 2 and Supplementary Data 1). The identifiers of all protein sequences contained in the HOGs returned by the search results were compiled and the GO enrichment of each cluster shown in Figure 4 was calculated using the OMA annotation corpus as a background. The enrichment results were manually parsed and salient annotations related to HOGs were selected to be reviewed further in the associated literature to check for the association of the search result with the query HOG (Table 2).

**Table 2. Manually curated biologically relevant search results for interactors coevolving with van Hooff *et al.*'s kinetochore and APC selected protein families** (van Hooff et al. 2017)**.** Protein families returned within clusters containing query HOGs are listed with their

pertinent annotation and literature. This is a non-exhaustive summary of some selected results. The full enrichment results are available as Supplementary Data 1.

| Cluster | Result | GO Term | Citation |
|---|---|---|---|
| APC1 | CFAP157 | GO:0035082 axoneme assembly | (Weidemann et al. 2016) |
| APC12 | C2CD3 | GO:0061511 centriole elongation | (Thauvin-Robinet et al. 2014) |
| CenpQ | ESCO2 | GO:0007059 chromosome segregation | (Lu et al. 2017) |
| KNL1 | TACC3 | GO:0007091 metaphase/anaphase transition of mitotic cell cycle | (Cheeseman et al. 2013) |

For instance, our search identified TACC3, which is known to be part of a structural stabilizer of kinetochore microtubules tension although it does not directly interact with the kinetochore complex (Cheeseman et al. 2013). ESCO2, a cohesin N-acetyltransferase needed for proper chromosome segregation during meiosis also plays a role in kinetochore-microtubule attachments regulation during meiosis (Lu et al. 2017). While these results are certainly promising, many of the unannotated proteins returned by our search likely contain more regulatory, metabolic and physical interactors which may prove to be interesting experimental targets.

**Search for a novel network**

Typical research use cases for profiling often involve uncharacterized protein families acting within poorly studied neworks. In this section we present search results for three HOGs known to be involved in the processes of meiosis, syngamy and karyogamy. Despite the ubiquitous nature of sex and its probable presence in LECA (Speijer, Lukeš, and Eliáš 2015), the protein networks

involved in each part of these processes have limited experimental data available, even in model organisms. Some key protein families involved in each step are known to have evolutionary patterns indicating an ancestral sequence in the LECA with subsequent modifications and losses (Speijer, Lukeš, and Eliáš 2015). The three following sections detail the returned results of the phylogenetic profiling pipeline with the Hap2, Gex1 and Spo11 families which all share this evolutionary pattern and are known to be critical for the process of gamete fusion, nuclear fusion and meiotic recombination, respectively. The proteins contained in the top 100 HOGs returned by the LSH Forest were analyzed for GO enrichment using all OMA annotations as a background. Due to the presence of biases in the GO annotation corpus (Altenhoff et al. 2012) we have also chosen to show the number of proteins annotated with each biological process selected from the enrichment out of the total number of annotated proteins.

### *Query with Hap2*

The Hap2 protein family has been shown to catalyze gamete membrane fusion in many eukaryotic clades and shares structural homology with viral and somatic membrane fusion proteins (Liu et al. 2008; Valansi et al. 2017; Fédry et al. 2017; Feng, Dong, and Springer 2018). A subset of the GO enrichment of the search results for the top 100 coevolving HOGs are shown below in Table 3.

**Table 3. Manually curated biologically relevant enriched GO terms from returned results.** The query sequence Hap2 is UniProt entry F4JP36 with OMA identifier ARATH26614 belonging to OMA HOG:0406399. The full enrichment results are available in the Supplementary Data 2.

| Term | Biological process | P-value | N-proteins |
|---|---|---|---|
| GO:0006338 | chromatin remodeling | 9.72e-54 | 61/3426 |
| GO:0048653 | anther development | 1.69e-35 | 17/3426 |
| GO:0009793 | embryo development ending in seed dormancy | 2.88e-13 | 15/3426 |
| GO:0051301 | cell division | 6.88e-16 | 5/3426 |

One particular family of interest which was returned in our search results is already characterized in angiosperms: LFR or leaf and flower related (Wang et al. 2012). This protein family is required for the development of reproductive structures in flowers and serves as a master regulator of the expression of many reproduction related genes, but its role in lower eukaryotes remains undescribed despite its broad evolutionary conservation.

### *Query with Gex1*

The nuclear fusion protein Gex1 is present in many of the same clades as Hap2, with a similar spotty pattern of absence across eukaryotes and a phylogeny indicating a vertical descent from LECA (Ning et al. 2013). A subset of the GO enrichment of the search results for the top 100 coevolving HOGs are shown below in Table 4.

**Table 4. Manually curated biologically relevant enriched GO terms from returned results.** The query sequence Gex1 is UniProt identifier Q681K7 with OMA identifier ARATH38809

belonging to OMA HOG:0416115. The full enrichment results are available as Supplementary Data 3.

| GO Term | P-value | N-Proteins |
|---|---|---|
| GO:0042753 positive regulation of circadian rhythm | 2.12e-285 | 113/2685 |
| GO:0048364 root development | 7.81e-125 | 70/2685 |
| GO:0051726 regulation of cell cycle | 1.22e-92 | 99/2685 |
| GO:0000712 resolution of meiotic recombination intermediates | 1.65e-47 | 26/2685 |
| GO:0007140 male meiotic nuclear division | 1.19e-39 | 26/2685 |
| GO:0009553 embryo sac development | 1.43e-28 | 17/2685 |
| GO:0022619 generative cell differentiation | 3.59e-18 | 5/2685 |

Gex1 has been shown to be involved in gamete development and embryogenesis (Alandete-Saez et al. 2011) and therefore GO terms 0022619 and 0009553 are applied to this protein. Thus proteins that HogProf identified as co-evolving with Gex1 and sharing these GO terms can be considered potential Gex1 interactors.

One search result of particular interest is a protein family which goes by the lyrical name of parting dancers (PTD). PTD belongs to a family that has been characterized in *Arabidopsis thaliana* and budding and fission yeast, and is known to be required in reciprocal homologous recombination during meiosis (Wijeratne et al. 2006). Our search shows that Gex1 co-evolved closely with PTD, a protein known to be involved in preparing genetic material for its eventual merger with another cell's nucleus.

***Query with Spo11***

The Spo11 helicase is involved in meiosis by catalyzing DNA double stranded breaks (DSBs) triggering homologous recombination. Spo11 is highly conserved throughout eukaryotes and homologues are present in almost all clades (Keeney, Giroux, and Kleckner 1997). A subset of the GO enrichment of the search results for the top 100 coevolving HOGs are shown below in Table 5.

**Table 5. Manually curated biologically relevant enriched GO terms from returned results.** The query sequence Spo11-1 is UniProt identifier Q9M4A2 with OMA identifier ARATH19148 belonging to OMA HOG:0605395. The full enrichment results are available in Supplementary Data 4.

| GO Term | P-value | N-Proteins |
|---|---|---|
| GO:0000737 DNA catabolic process, endonucleolytic | 0.00e+00 | 415/20562 |
| GO:0043137 DNA replication, removal of RNA primer | 0.00e+00 | 353/20562 |
| GO:0006275 regulation of DNA replication | 0.00e+00 | 552/20562 |
| GO:0006302 double-strand break repair | 8.11e-242 | 285/20562 |
| GO:0007292 female gamete generation | 2.71e-184 | 136/20562 |
| GO:0022414 reproductive process | 1.66e-93 | 127/20562 |

It is encouraging to find that Spo11, the trigger of meiotic DSBs, has co-evolved with other families involved in the inverse process of repairing the DSBs and finishing the process of recombination (Table 5). Other identified HOGs contain annotations such as gamete generation and reproduction also focusing on processes that result in cellular commitment to a gamete cell fate through meiosis. Proliferating cell nuclear antigen or PCNA (Strzalka and Ziemienowicz 2011) was also retrieved by our search. This ubiquitous protein family is an auxiliary scaffold protein to the DNA

polymerase and recruits other interactors to the polymerase complex to repair damaged DNA, making it an interesting candidate for a potential physical interactor with Spo11.

### *A broader search for the reproductive network*

A more in-depth treatment of the evolutionary conservation of gamete cell fate commitment and mating is available in previous publications (Malik et al. 2007; Loidl 2016; Speijer, Lukeš, and Eliáš 2015; Ning et al. 2013; Schurko and Logsdon 2008; Niklas, Cobb, and Kutschera 2014; Goodenough and Heitman 2014). Using these sources, a list of broadly conserved protein families known to be involved in sexual reproduction were compiled to be used as HOG queries to the LSH Forest to retrieve the top 10 closest coevolving HOGs. The hash signatures of the queries and results were compiled and used in an all-vs-all comparison to generate a Jaccard distance matrix.

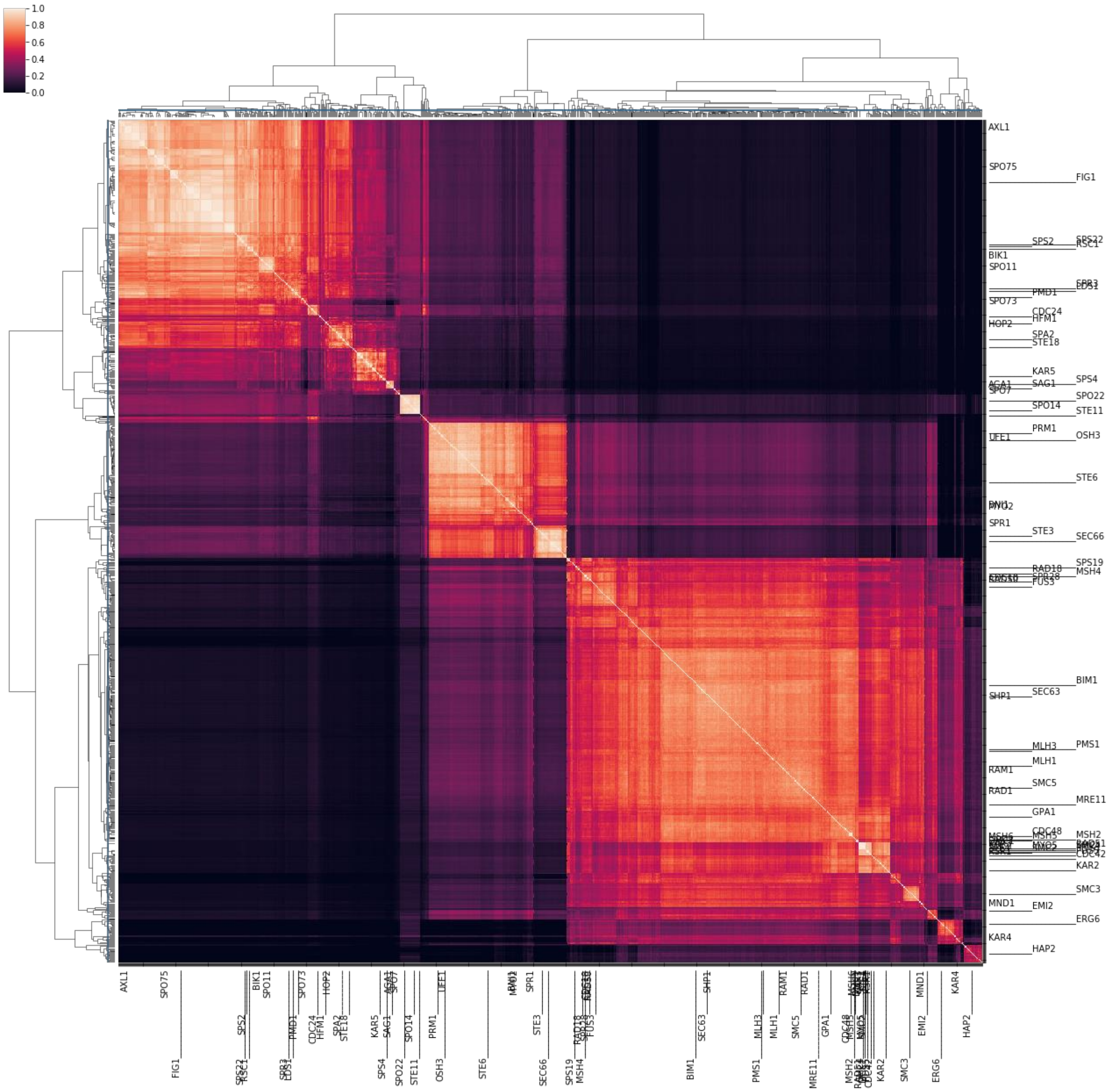


**Fig. 5. HogProf's reproductive network.** A list of proteins known to be involved in sexual reproduction was compiled and mapped to OMA HOGs. These queries were used to search for the 20 closest coevolving HOGs in an LSH forest containing all HOGs in OMA. A Jaccard kernel was generated by performing an All vs All comparison of the Hash signatures of search results and queries. UPGMA clustering was performed on the rows and columns of the kernel. A cutoff

distance of .995 ( blue lines ) was manually chosen to limit cluster sizes to less than 50 HOGs. This generated a total of 215 clusters of HOGs. Names for queries are shown with *Saccharomyces cerevisiae* gene names (apart from Hap2 which is not present in fungi ).

The all-vs-all comparison of the Jaccard distances between these returned HOGs reveals clusters of putative interactors co-evolving closely with specific parts of the sexual reproduction network. Manual analysis of GO enrichment results revealed several sexual reproduction-related proteins which are summarized in Table 6. In addition to annotated protein sequences and HOGs, many unannotated, coevolving HOGs were found.

Particularly for biological processes as complex and evolutionarily diverse as sexual reproduction, GO annotations are, unsurprisingly, incomplete. Fortunately, our profiling approach is successful in identifying protein families with similar evolutionary patterns that have already been characterised and are directly relevant to sexual reproduction (Table 6). By considering the uncharacterized or poorly characterized families at the sequence and structure level, we may be able to predict their functions and reconstitute their local interactome. Our ultimate goal is to guide *in vivo* experiments to test and characterize these targets within the broader context of eukaryotic sexual reproduction.

**Table 6. Manually curated biologically relevant putative interactors from sexual reproduction search results.** Protein families within clusters containing query HOGs are listed with their pertinent annotation and literature. GO enrichment results of clusters containing one or more queries were analyzed manually. Full enrichment results are available in the Supplementary Data 5.

| Cluster | Result | GO Term | Citation |
|---|---|---|---|

| | | | |
|---|---|---|---|
| REC8 | NSE4 | GO:0030915 Smc5-Smc6 complex | (Zelkowski et al. 2019) |
| SPC72 | MID2 | GO:0000767 cell morphogenesis involved in conjugation | (Rajavel et al. 1999) |
| SPO71 | LES2 | GO:0031011 Ino80 complex | (Serber et al. 2016; Bao and Shen 2011) |
| SHC1, SPO16 | POG1 | GO:0000321 re-entry into mitotic cell cycle after pheromone arrest | (Leza and Elion 1999; van Werven et al. 2012) |

This example related to the ancestral sexual reproduction network illustrates the utility of the LSH Forest search functionality and OMA resources in exploratory characterization of poorly described networks. The interactions presented above ( Table 6 ) only represent our limited effort to manually review literature to highlight potentially credible interactions detected by our pipeline. Again, as was the case with our kinetochore and APC related searches, several interactions might not appear obvious on their face. For example, SPC72 and MID2 are both involved in meiotic processes but localized to different parts of the cell ( centriole and plasma membrane, respectively). However, it has been shown that microtubule organization and membrane integrity sensing pathways do show interaction during gamete maturation (Gordon et al. 2006).

**Discussion**

We introduced a scalable system for phylogenetic profiling from hierarchical orthologous groups. The ROC and AUC values shown using an empirical benchmark (Results Section) ~~in the first section of Results~~ indicates that the MinHash Jaccard score estimate between profiles has slightly better performance than previous tree and vector based metrics, while also being much faster to compute. This is remarkable in that one typically expects a trade-off between speed and accuracy, which does not

appear to be the case here. We hypothesise that the error introduced by the MinHash approximation is compensated by the inclusion of an unprecedented amount of genomes and taxonomic nodes in the labelled phylogenies used to construct the profiles.

Furthermore, while our MinHash-derived Jaccard estimates are able to capture some of the differences between interacting and non-interacting HOGs, as shown above, their unique strength lies in the fast recovery of the top k closest profiles within an LSH Forest. Once these profiles are recovered, the inference of submodules or network structure can be refined using other, potentially more compute intensive methods, on this much smaller subset of data.

We have shown that HogProf is able to reconstitute the modular organisation of the kinetochore, as well as increase the list of protein families interacting within the network with several known interactors of the kinetochore and the APC. As for the other HOGs returned in these searches, our results suggest that some are yet unknown interactors involved in aspects of the cell cycle or ciliary dynamics. Likewise, our attempt at retrieving candidate members of the sexual reproduction network recapitulated many known interactions, while also suggesting new ones.

The current paradigm for exploring interaction or participation in different biological pathways across protein families relies heavily on data integration strategies that take into account heterogenous high-throughput experiments and knowledge found in the literature. Many times, these datasets only describe the networks in question in one organism at a time. Furthermore, signaling, metabolic and physical interaction networks are all covered by different types of experiments and data produced by these systems is located in heterogeneous databases. By contrast, phylogenetic profiles can potentially uncover all three types of networks from sequencing

data alone. This was highlighted in our work during retrieval of potential interactors within the sexual reproduction and kinetochore networks with the retrieval of LFR and CFAP157, respectively. CFAP157, a cilia and flagella associated protein might be involved in recruitment/regulation of APC-Cdc20 or ciliary kinases (e.g Nek1), both known to mediate APC regulation of ciliary dynamics (Wang and Kirschner, 2014). In both cases, a regulatory action within the network was the biological process which involved both the query and retrieved HOGs, not a physical interaction. The advances put forward by our new methodology and the property of retrieving entire networks and not just physical interactions opens the possibility of performing comparative profiling on an unprecedented scale and lays the groundwork for integrative modeling of the interplay between PPI, regulation and metabolic networks in a more holistic way.

Further work remains to be done on tuning the profile construction with the appropriate weights at each taxonomic level, as well as constructing profiles for subfamiles arising from duplications which may undergo neofunctionalization, a theme which has been previously explored in phylogenetic profiling efforts relying on far fewer genomes (Dey et al. 2015). Downstream processing of the explicit representation of the data, as opposed to the the hash signature, can also be designed using more computationally intensive methods to detect interactions on smaller subsets of profiles after using the LSH as a first search.

The phylogenetic profiling pipeline presented in this work will be integrated into OMA web-based services. Meanwhile, it is already available on Github as a standalone package ( https://github.com/DessimozLab/HogProf).

## Methods

The following section details the creation of phylogenetic profiles using OMA data, their transformation into MinHash based probabilistic data structures and the tools and libraries used in the implementation.

**Profile construction**

To generate large-scale gene phylogenies labelled with speciation, duplication and loss events (a.k.a. *enhanced phylogenies* or *tree profiles*) for each HOG in OMA, we processed input data in OrthoXML format (Schmitt et al. 2011) with pyHam (Train et al. 2018), using the NCBI taxonomic tree (Sayers et al. 2010) pruned to contain only the genomes represented in OMA (Altenhoff et al. 2018). Tree profiles contain a species tree annotated at each taxonomic level with information on when the last common ancestor gene appeared, where losses and duplications occurred and the copy number of the gene at each taxonomic level. More information on the pyHam inference of evolutionary events can be found in (Train et al. 2018). pyHam can also be used to infer enhanced phylogenies for other datasets available in OrthoXML format such as ENSEMBL (Zerbino et al. 2018) or with data generated from phylogenetic trees such as those found in PANTHER (Mi et al. 2017) through the use of the function `etree2orthoxml()` in the tree analysis package ETE3 (Huerta-Cepas, Serra, and Bork 2016).

The enhanced phylogeny trees for each HOG are parsed to create a vector representation of the presence or absence of a homologue at each extant and ancestral node as well as the duplication or loss events on the branch leading to that node. Each profile vector contains 9345 columns ( corresponding to the 3115 nodes of the taxonomy used and the 3 categories of presence, loss and duplication ).

To encode profile vectors as weighted MinHash signatures (Sergey Ioffe 2010) we used the Datasketch library ("Datasketch: Big Data Looks Small — Datasketch 1.0.0 Documentation" n.d.). In this formulation, the Jaccard score between multisets representing profiles can be more heavily influenced by nodes with a higher weight. The final MinHash signatures used were built with 256 hashing functions.

After transforming HOG profile vectors to their corresponding weighted MinHashes using the datasketch library, an estimation of the Jaccard distance between profiles can be obtained by calculating the Hamming distance between their hash signatures (S. Ioffe 2010). The speed of comparison and lower bound for accuracy of the estimation of the Jaccard score is set by the number of hashing functions. The comparison of hash signatures has $O(N)$ time complexity where N is the number of hash functions used to generate the MinHash signature. Due to this property, an arbitrary number of elements can be encoded in this signature without slowing down comparisons. In our use case, this enables the use of an arbitrarily large number of taxa for which we can consider evolutionary events. Additionally, hardware implementations of hash functions allow the calculation of hash signatures at rates of giga hashes per second and allow for extremely fast implementation of this step, placing the bottleneck of the pipeline at the calculation of enhanced phylogenies.

The weighted MinHash objects for each HOG's enhanced phylogeny were compiled into a searchable data structure referred to as a Locality Sensitive Hashing Forest (LSH Forest) (Bawa, Condie, and Ganesan 2005) and their signatures were stored in an HDF5 file. The LSH Forest can be queried with a hash signature to retrieve the K neighbors with the highest Jaccard similarity to the query hash. The K closest hashes are retrieved from a B-Tree data structure (Comer 1979).

This branching tree data structure allows for the querying and dynamic insertion and deletion of elements in the LSH Forest data structure built upon it with logarithmic time complexity.

The scaling properties of the MinHash data structures when compared to pairwise distance calculations and hierarchical clustering are shown below in Figure 6.

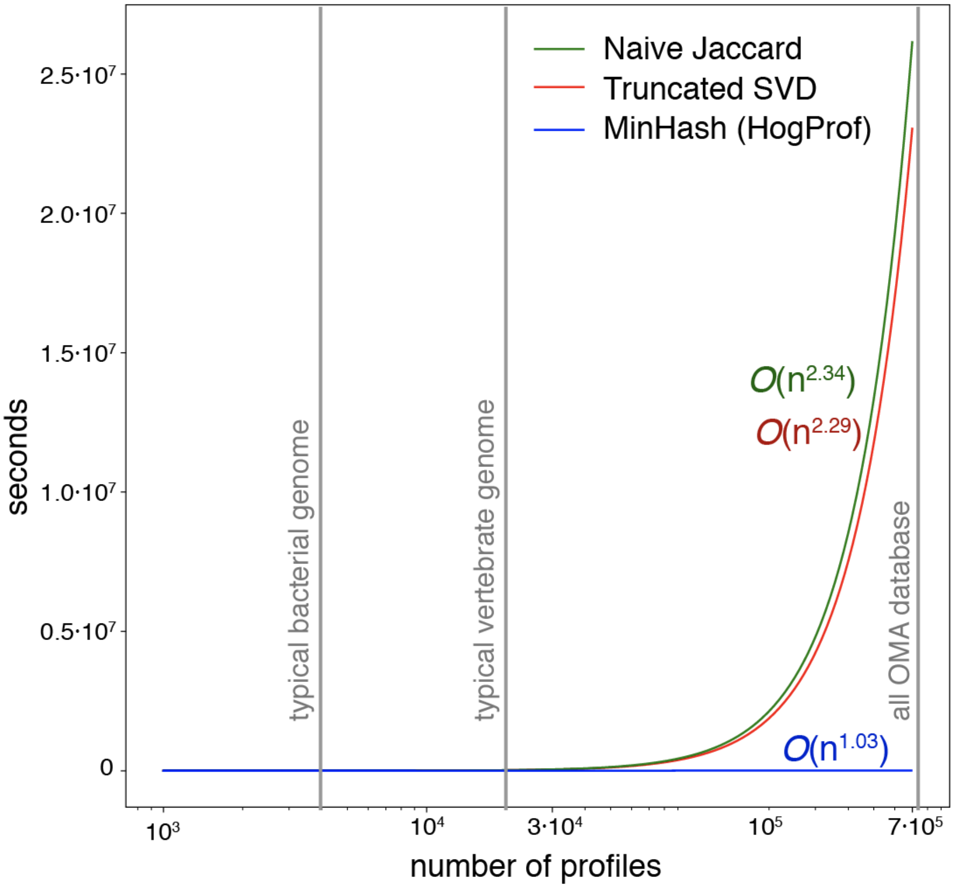


**Fig. 6.** To illustrate the advantageous scaling properties of MinHash data structures, synthetic profiles of length 100 were generated in the form of binary vectors (0 and 1 equiprobable). Profiles were then clustered using an explicit calculation of the Jaccard distance, reduced to a lower dimensionality (5 dimensions) with truncated SVD, normalized and explicitly clustered using Euclidean distance as in SVD-Phy (Franceschini et al. 2016) or transformed into MinHash

signatures and inserted into an LSH Forest object as in our method. Orders of magnitude showing typical use cases for profiling pipelines are shown on the x-axis. Curves were fitted to each set of timepoints to empirically determine the time complexity of each approach.

**Computational resources, data and libraries**

Our dataset contains approximately 600,000 HOGs computed from the 2,167 genomes in OMA (June 2018 release) The main computational bottleneck in our pipeline is the calculation of the labelled gene trees for each HOG using pyHam. Even with this computation, compiled LSH forest objects containing the hash signatures of all HOGs' gene trees can be compiled in under 3 hours (with 10 CPUs but this can scale easily to more cores) with only 2.5 GB of RAM and queried extremely efficiently (an average of 0.01 seconds over 1000 queries against a database containing profiles for all HOGs in OMA on an Intel(R) Xeon(R) CPU E5530 @ 2.40 GHz and 2 GB of RAM to load the LSH database object into memory). This performance makes it possible to provide online search functionality, which we aim to release in an upcoming web-based version of the OMA browser. Meanwhile, the compiled profile database can be used for analysis on typical workstations (note that memory and CPU requirements will depend on the number of hash functions implemented in the construction of profiles and the filtering of the initial dataset to clades of interest to the user).

All gene ontology (GO) annotations (encompassing molecular functions, cellular locations, and biological processes) for HOGs contained in OMA were analyzed with GOATOOLS (Klopfenstein et al. 2018). To calculate the enrichment of annotations, the results returned by the LSH Forest

annotations for all protein sequences contained in the HOGs returned by the search were collected and the entire OMA annotation corpus was used as background.

HDF5 files were compiled with H5PY (ver. 2.9.0). Pandas (ver. 0.24.0) was used for data manipulation. Labelled phylogenies were manipulated with ETE3 (Huerta-Cepas, Serra, and Bork 2016). Datasketch (ver. 1.0.0) was used to compile weighted MinHashes and LSH Forest data structures. Plots were generated using matplotlib (ver. 3.0.2). PyHam (ver 1.1.6) was used to calculate labelled phylogenies for the HOGs in OMA.

Time complexity analysis in Figure 6 was done with the scikit-learn implementation of truncated SVD (Pedregosa et al. 2011) and scipy (Jones, Oliphant, and Peterson 2001) distance functions.

**Pearson and Spearman correlation comparison of distance matrices**

Distance matrices between all pairs of profiles in the kinetochore and APC complex protein families defined in (van Hooff et al. 2017) were compared using the Spearman and Pearson statistical analysis functions from the the SciPy python package to verify the monotonicity of the scores between families.

## Acknowledgements

We thank Monique Zahn for helpful feedback on the manuscript. This work was funded by a grant by the Novartis Foundation for Medical-Biological Research (#17B111 to CD), by the Swiss National Science Foundation (Grant 183723 to CD), by the Swiss Leading House for the Latin American Region (to CD and PSA), and by the Agencia Nacional de Promoción Científica y Tecnológica (PICT-2017-0854 to PSA) .

*Conflict of Interest:* none declared.

## Supplementary data

- **Supplementary Data 1—kineto_augment_goenrich.csv**: Contains the results of GO enrichment analysis done on the results of our search for kinetochore interactors. After searching with the HOGs corresponding to each of the kinetochore components, the returned HOGs were clustered according to their jaccard similarity. Using a hierarchical clustering and a manually defined cutoff the results were separated into discrete clusters. Each cluster was analyzed using goatools for GO enrichment. Enrichment results for clusters containing a query gene were recorded in this CSV file.
- **Supplementary Data 2—hap_enrich.csv**: Contains the goatools output for the GO enrichment analysis of the top 100 closest coevolving HOGs returned by a query with Hap2.
- **Supplementary Data 3—gex_enrich.csv**: Contains the goatools output for the GO enrichment analysis of the top 100 closest coevolving HOGs returned by a query with Gex1.
- **Supplementary Data 5—repro_augment_goenrich.csv:** Contains the results of GO enrichment analysis done on the results of our search for sexual reproduction network interactors. After searching with the HOGs corresponding to each of the manually curated list of conserved sexual reporduction network components, the returned HOGs were clustered according to their jaccard similarity. Using a hierarchical clustering and a manually defined cutoff the results were separated into discrete clusters. Each cluster was analyzed using goatools for GO enrichment. Enrichment results for clusters containing a query were recorded in this csv file.
- **Supplementary Data 4- repro_hogs.csv**: Contains a manually selected set of highly conserved protein families involved in sexual reproduction.

## References

Alandete-Saez, Monica, Mily Ron, Samuel Leiboff, and Sheila McCormick. 2011. "Arabidopsis Thaliana GEX1 Has Dual Functions in Gametophyte Development and Early Embryogenesis: Dual Functions of GEX1." *The Plant Journal: For Cell and Molecular Biology* 68 (4): 620–32.

Altenhoff, Adrian M., Manuel Gil, Gaston H. Gonnet, and Christophe Dessimoz. 2013. "Inferring Hierarchical Orthologous Groups from Orthologous Gene Pairs." *PloS One* 8 (1): e53786.

Altenhoff, Adrian M., Natasha M. Glover, Clément-Marie Train, Klara Kaleb, Alex Warwick Vesztrocy, David Dylus, Tarcisio M. de Farias, et al. 2018. "The OMA Orthology Database in 2018: Retrieving Evolutionary Relationships among All Domains of Life through Richer Web and Programmatic Interfaces." *Nucleic Acids Research* 46 (D1): D477–85.

Altenhoff, Adrian M., Romain A. Studer, Marc Robinson-Rechavi, and Christophe Dessimoz. 2012. "Resolving the Ortholog Conjecture: Orthologs Tend to Be Weakly, but Significantly, More Similar in Function than Paralogs." *PLoS Computational Biology* 8 (5): e1002514.

Ashburner, M., C. A. Ball, J. A. Blake, D. Botstein, H. Butler, J. M. Cherry, A. P. Davis, et al. 2000. "Gene Ontology: Tool for the Unification of Biology. The Gene Ontology Consortium." *Nature Genetics* 25 (1): 25–29.

Bao, Yunhe, and Xuetong Shen. 2011. "SnapShot: Chromatin Remodeling: INO80 and SWR1." *Cell* 144 (1): 158–158.e2.

Bawa, Mayank, Tyson Condie, and Prasanna Ganesan. 2005. "LSH Forest: Self-Tuning Indexes for Similarity Search." In *Proceedings of the 14th International Conference on World Wide Web*, 651–60. WWW '05. New York, NY, USA: ACM.

Cheeseman, Liam P., Edward F. Harry, Andrew D. McAinsh, Ian A. Prior, and Stephen J. Royle. 2013. "Specific Removal of TACC3-Ch-TOG-Clathrin at Metaphase Deregulates Kinetochore Fiber Tension." *Journal of Cell Science* 126 (Pt 9): 2102–13.

Comer, Douglas. 1979. "Ubiquitous B-Tree." *ACM Computing Surveys (CSUR)* 11 (2): 121–37.

Cozzetto, Domenico, and David T. Jones. 2017. "Computational Methods for Annotation Transfers from Sequence." *Methods in Molecular Biology* 1446: 55–67.

"Datasketch: Big Data Looks Small — Datasketch 1.0.0 Documentation." n.d. Accessed September 26, 2018. https://ekzhu.github.io/datasketch/index.html.

Dessimoz, Christophe, Nives Škunca, and Paul D. Thomas. 2013. "CAFA and the Open World of Protein Function Predictions." *Trends in Genetics: TIG* 29 (11): 609–10.

Dey, Gautam, Ariel Jaimovich, Sean R. Collins, Akiko Seki, and Tobias Meyer. 2015. "Systematic Discovery of Human Gene Function and Principles of Modular Organization through Phylogenetic Profiling." *Cell Reports*, February. https://doi.org/10.1016/j.celrep.2015.01.025.

Fédry, Juliette, Yanjie Liu, Gérard Péhau-Arnaudet, Jimin Pei, Wenhao Li, M. Alejandra Tortorici, François Traincard, et al. 2017. "The Ancient Gamete Fusogen HAP2 Is a Eukaryotic Class II Fusion Protein." *Cell* 168 (5): 904–15.e10.

Feng, J., X. Dong, and T. A. Springer. 2018. "Fusion Surface Structure, Function, and Dynamics of Gamete

Fusogen HAP2." https://doi.org/10.2210/pdb6dbs/pdb.

Franceschini, Andrea, Jianyi Lin, Christian von Mering, and Lars Juhl Jensen. 2016. "SVD-Phy: Improved Prediction of Protein Functional Associations through Singular Value Decomposition of Phylogenetic Profiles." *Bioinformatics* 32 (7): 1085–87.

Giurgiu, Madalina, Julian Reinhard, Barbara Brauner, Irmtraud Dunger-Kaltenbach, Gisela Fobo, Goar Frishman, Corinna Montrone, and Andreas Ruepp. 2018. "CORUM: The Comprehensive Resource of Mammalian Protein Complexes-2019." *Nucleic Acids Research*, October. https://doi.org/10.1093/nar/gky973.

Glazko, Galina V., and Arcady R. Mushegian. 2004. "Detection of Evolutionarily Stable Fragments of Cellular Pathways by Hierarchical Clustering of Phyletic Patterns." *Genome Biology* 5 (5): R32.

Goodenough, Ursula, and Joseph Heitman. 2014. "Origins of Eukaryotic Sexual Reproduction." *Cold Spring Harbor Perspectives in Biology* 6 (3). https://doi.org/10.1101/cshperspect.a016154.

Gordon, Oren, Christof Taxis, Philipp J. Keller, Aleksander Benjak, Ernst H. K. Stelzer, Giora Simchen, and Michael Knop. 2006. "Nud1p, the Yeast Homolog of Centriolin, Regulates Spindle Pole Body Inheritance in Meiosis." *The EMBO Journal* 25 (16): 3856–68.

Hooff, Jolien Je van, Eelco Tromer, Leny M. van Wijk, Berend Snel, and Geert Jpl Kops. 2017. "Evolutionary Dynamics of the Kinetochore Network in Eukaryotes as Revealed by Comparative Genomics." *EMBO Reports* 18 (9): 1559–71.

Huerta-Cepas, Jaime, François Serra, and Peer Bork. 2016. "ETE 3: Reconstruction, Analysis, and Visualization of Phylogenomic Data." *Molecular Biology and Evolution* 33 (6): 1635–38.

Huerta-Cepas, Jaime, Damian Szklarczyk, Kristoffer Forslund, Helen Cook, Davide Heller, Mathias C. Walter, Thomas Rattei, et al. 2016. "eggNOG 4.5: A Hierarchical Orthology Framework with Improved Functional Annotations for Eukaryotic, Prokaryotic and Viral Sequences." *Nucleic Acids Research* 44 (D1): D286–93.

Ioffe, S. 2010. "Improved Consistent Sampling, Weighted Minhash and L1 Sketching." In *2010 IEEE International Conference on Data Mining*, 246–55.

Ioffe, Sergey. 2010. "Improved Consistent Sampling, Weighted Minhash and textL1 Sketching ICDM." *Sydney, AU.*

Jones, Eric, Travis Oliphant, and Pearu Peterson. 2001. "SciPy: Open Source Scientific Tools for Python." https://www.scienceopen.com/document?vid=ab12905a-8a5b-43d8-a2bb-defc771410b9.

Jothi, Raja, Teresa M. Przytycka, and L. Aravind. 2007. "Discovering Functional Linkages and Uncharacterized Cellular Pathways Using Phylogenetic Profile Comparisons: A Comprehensive Assessment." *BMC Bioinformatics* 8 (May): 173.

Keeney, S., C. N. Giroux, and N. Kleckner. 1997. "Meiosis-Specific DNA Double-Strand Breaks Are Catalyzed by Spo11, a Member of a Widely Conserved Protein Family." *Cell* 88 (3): 375–84.

Kensche, Philip R., Vera van Noort, Bas E. Dutilh, and Martijn A. Huynen. 2008. "Practical and Theoretical Advances in Predicting the Function of a Protein by Its Phylogenetic Distribution." *Journal of the*

*Royal Society, Interface / the Royal Society* 5 (19): 151–70.

Klopfenstein, D. V., Liangsheng Zhang, Brent S. Pedersen, Fidel Ramírez, Alex Warwick Vesztrocy, Aurélien Naldi, Christopher J. Mungall, et al. 2018. "GOATOOLS: A Python Library for Gene Ontology Analyses." *Scientific Reports* 8 (1): 10872.

Leza, M. A., and E. A. Elion. 1999. "POG1, a Novel Yeast Gene, Promotes Recovery from Pheromone Arrest via the G1 Cyclin CLN2." *Genetics* 151 (2): 531–43.

Liu, Yanjie, Rita Tewari, Jue Ning, Andrew M. Blagborough, Sara Garbom, Jimin Pei, Nick V. Grishin, et al. 2008. "The Conserved Plant Sterility Gene HAP2 Functions after Attachment of Fusogenic Membranes in Chlamydomonas and Plasmodium Gametes." *Genes & Development* 22 (8): 1051–68.

Li, Yang, Sarah E. Calvo, Roee Gutman, Jun S. Liu, and Vamsi K. Mootha. 2014. "Expansion of Biological Pathways Based on Evolutionary Inference." *Cell* 158 (1): 213–25.

Loidl, Josef. 2016. "Conservation and Variability of Meiosis Across the Eukaryotes." *Annual Review of Genetics* 50 (November): 293–316.

Lu, Yajuan, Xiaoxin Dai, Mianqun Zhang, Yilong Miao, Changyin Zhou, Zhaokang Cui, and Bo Xiong. 2017. "Cohesin Acetyltransferase Esco2 Regulates SAC and Kinetochore Functions via Maintaining H4K16 Acetylation during Mouse Oocyte Meiosis." *Nucleic Acids Research* 45 (16): 9388–97.

Malik, Shehre-Banoo, Arthur W. Pightling, Lauren M. Stefaniak, Andrew M. Schurko, and John M. Logsdon Jr. 2007. "An Expanded Inventory of Conserved Meiotic Genes Provides Evidence for Sex in Trichomonas Vaginalis." *PloS One* 3 (8): e2879.

Mewes, H. W., C. Amid, R. Arnold, D. Frishman, U. Güldener, G. Mannhaupt, M. Münsterkötter, et al. 2004. "MIPS: Analysis and Annotation of Proteins from Whole Genomes." *Nucleic Acids Research* 32 (Database issue): D41–44.

Mi, Huaiyu, Xiaosong Huang, Anushya Muruganujan, Haiming Tang, Caitlin Mills, Diane Kang, and Paul D. Thomas. 2017. "PANTHER Version 11: Expanded Annotation Data from Gene Ontology and Reactome Pathways, and Data Analysis Tool Enhancements." *Nucleic Acids Research* 45 (D1): D183–89.

Mukherjee, Supratim, Dimitri Stamatis, Jon Bertsch, Galina Ovchinnikova, Olena Verezemska, Michelle Isbandi, Alex D. Thomas, et al. 2017. "Genomes OnLine Database (GOLD) v.6: Data Updates and Feature Enhancements." *Nucleic Acids Research* 45 (D1): D446–56.

Nevers, Yannis, Megana K. Prasad, Laetitia Poidevin, Kirsley Chennen, Alexis Allot, Arnaud Kress, Raymond Ripp, et al. 2017. "Insights into Ciliary Genes and Evolution from Multi-Level Phylogenetic Profiling." *Molecular Biology and Evolution* 34 (8): 2016–34.

Niklas, Karl J., Edward D. Cobb, and Ulrich Kutschera. 2014. "Did Meiosis Evolve before Sex and the Evolution of Eukaryotic Life Cycles?" *BioEssays: News and Reviews in Molecular, Cellular and Developmental Biology* 36 (11): 1091–1101.

Ning, Jue, Thomas D. Otto, Claudia Pfander, Frank Schwach, Mathieu Brochet, Ellen Bushell, David Goulding, et al. 2013. "Comparative Genomics in Chlamydomonas and Plasmodium Identifies an Ancient Nuclear Envelope Protein Family Essential for Sexual Reproduction in Protists, Fungi, Plants,

and Vertebrates." *Genes & Development* 27 (10): 1198–1215.

Niu, Yulong, Chengcheng Liu, Shayan Moghimyfiroozabad, Yi Yang, and Kambiz N. Alavian. 2017. "PrePhyloPro: Phylogenetic Profile-Based Prediction of Whole Proteome Linkages." *PeerJ* 5 (August): e3712.

Pedregosa, Fabian, Gaël Varoquaux, Alexandre Gramfort, Vincent Michel, Bertrand Thirion, Olivier Grisel, Mathieu Blondel, et al. 2011. "Scikit-Learn: Machine Learning in Python." *Journal of Machine Learning Research: JMLR* 12 (Oct): 2825–30.

Pellegrini, M., E. M. Marcotte, M. J. Thompson, D. Eisenberg, and T. O. Yeates. 1999. "Assigning Protein Functions by Comparative Genome Analysis: Protein Phylogenetic Profiles." *Proceedings of the National Academy of Sciences of the United States of America* 96 (8): 4285–88.

Rajavel, M., B. Philip, B. M. Buehrer, B. Errede, and D. E. Levin. 1999. "Mid2 Is a Putative Sensor for Cell Integrity Signaling in Saccharomyces Cerevisiae." *Molecular and Cellular Biology* 19 (6): 3969–76.

Ranea, Juan A. G., Corin Yeats, Alastair Grant, and Christine A. Orengo. 2007. "Predicting Protein Function with Hierarchical Phylogenetic Profiles: The Gene3D Phylo-Tuner Method Applied to Eukaryotic Genomes." *PLoS Computational Biology* 3 (11): e237.

Ruano-Rubio, Valentín, Olivier Poch, and Julie D. Thompson. 2009. "Comparison of Eukaryotic Phylogenetic Profiling Approaches Using Species Tree Aware Methods." *BMC Bioinformatics* 10 (November): 383.

Sayers, Eric W., Tanya Barrett, Dennis a. Benson, Evan Bolton, Stephen H. Bryant, Kathi Canese, Vyacheslav Chetvernin, et al. 2010. "Database Resources of the National Center for Biotechnology Information." *Nucleic Acids Research* 38 (Database issue): D5–16.

Schmitt, Thomas, David N. Messina, Fabian Schreiber, and Erik L. L. Sonnhammer. 2011. "Letter to the Editor: SeqXML and OrthoXML: Standards for Sequence and Orthology Information." *Briefings in Bioinformatics* 12 (5): 485–88.

Schurko, Andrew M., and John M. Logsdon Jr. 2008. "Using a Meiosis Detection Toolkit to Investigate Ancient Asexual 'Scandals' and the Evolution of Sex." *BioEssays: News and Reviews in Molecular, Cellular and Developmental Biology* 30 (6): 579–89.

Serber, Daniel W., John S. Runge, Debashish U. Menon, and Terry Magnuson. 2016. "The Mouse INO80 Chromatin-Remodeling Complex Is an Essential Meiotic Factor for Spermatogenesis." *Biology of Reproduction* 94 (1): 8.

Sherill-Rofe, Dana, Dolev Rahat, Steven Findlay, Anna Mellul, Irene Guberman, Maya Braun, Idit Bloch, et al. 2019. "Mapping Global and Local Coevolution across 600 Species to Identify Novel Homologous Recombination Repair Genes." *Genome Research*. https://doi.org/10.1101/gr.241414.118.

Skunca, Nives, Adrian Altenhoff, and Christophe Dessimoz. 2012. "Quality of Computationally Inferred Gene Ontology Annotations." *PLoS Computational Biology* 8 (5): e1002533.

Snitkin, Evan S., Adam M. Gustafson, Joseph Mellor, Jie Wu, and Charles DeLisi. 2006. "10.1186/1471-2105-7-420." *BMC Bioinformatics*. https://doi.org/10.1186/1471-2105-7-420.

Speijer, Dave, Julius Lukeš, and Marek Eliáš. 2015. "Sex Is a Ubiquitous, Ancient, and Inherent Attribute of

Eukaryotic Life." *Proceedings of the National Academy of Sciences of the United States of America* 112 (29): 8827–34.

Strzalka, Wojciech, and Alicja Ziemienowicz. 2011. "Proliferating Cell Nuclear Antigen (PCNA): A Key Factor in DNA Replication and Cell Cycle Regulation." *Annals of Botany* 107 (7): 1127–40.

Szklarczyk, Damian, John H. Morris, Helen Cook, Michael Kuhn, Stefan Wyder, Milan Simonovic, Alberto Santos, et al. 2017. "The STRING Database in 2017: Quality-Controlled Protein-Protein Association Networks, Made Broadly Accessible." *Nucleic Acids Research* 45 (D1): D362–68.

Tabach, Yuval, Allison C. Billi, Gabriel D. Hayes, Martin A. Newman, Or Zuk, Harrison Gabel, Ravi Kamath, et al. 2013. "Identification of Small RNA Pathway Genes Using Patterns of Phylogenetic Conservation and Divergence." *Nature* 493 (7434): 694–98.

Ta, Hung Xuan, Patrik Koskinen, and Liisa Holm. 2011. "A Novel Method for Assigning Functional Linkages to Proteins Using Enhanced Phylogenetic Trees." *Bioinformatics* 27 (5): 700–706.

"TAIR - Portals - Genome Snapshot." n.d. Accessed February 19, 2020. https://www.arabidopsis.org/portals/genAnnotation/genome_snapshot.jsp.

Thauvin-Robinet, Christel, Jaclyn S. Lee, Estelle Lopez, Vicente Herranz-Pérez, Toshinobu Shida, Brunella Franco, Laurence Jego, et al. 2014. "The Oral-Facial-Digital Syndrome Gene C2CD3 Encodes a Positive Regulator of Centriole Elongation." *Nature Genetics* 46 (8): 905–11.

Train, Clément-Marie, Miguel Pignatelli, Adrian Altenhoff, and Christophe Dessimoz. 2018. "iHam & pyHam: Visualizing and Processing Hierarchical Orthologous Groups." *Bioinformatics* , December. https://doi.org/10.1093/bioinformatics/bty994.

Valansi, Clari, David Moi, Evgenia Leikina, Elena Matveev, Martín Graña, Leonid V. Chernomordik, Héctor Romero, Pablo S. Aguilar, and Benjamin Podbilewicz. 2017. "Arabidopsis HAP2/GCS1 Is a Gamete Fusion Protein Homologous to Somatic and Viral Fusogens." *The Journal of Cell Biology*.

Wang, Xiu-Tang, Can Yuan, Ting-Ting Yuan, and Su-Juan Cui. 2012. "The Arabidopsis LFR Gene Is Required for the Formation of Anther Cell Layers and Normal Expression of Key Regulatory Genes." *Molecular Plant* 5 (5): 993–1000.

Weidemann, Marina, Karin Schuster-Gossler, Michael Stauber, Christoph Wrede, Jan Hegermann, Tim Ott, Karsten Boldt, et al. 2016. "CFAP157 Is a Murine Downstream Effector of FOXJ1 That Is Specifically Required for Flagellum Morphogenesis and Sperm Motility." *Development* 143 (24): 4736–48.

Werven, Folkert J. van, Gregor Neuert, Natalie Hendrick, Aurélie Lardenois, Stephen Buratowski, Alexander van Oudenaarden, Michael Primig, and Angelika Amon. 2012. "Transcription of Two Long Noncoding RNAs Mediates Mating-Type Control of Gametogenesis in Budding Yeast." *Cell* 150 (6): 1170–81.

Wijeratne, Asela J., Changbin Chen, Wei Zhang, Ljudmilla Timofejeva, and Hong Ma. 2006. "The Arabidopsis Thaliana PARTING DANCERS Gene Encoding a Novel Protein Is Required for Normal Meiotic Homologous Recombination." *Molecular Biology of the Cell* 17 (3): 1331–43.

Zdobnov, Evgeny M., Fredrik Tegenfeldt, Dmitry Kuznetsov, Robert M. Waterhouse, Felipe A. Simão, Panagiotis Ioannidis, Mathieu Seppey, Alexis Loetscher, and Evgenia V. Kriventseva. 2017. "OrthoDB

v9.1: Cataloging Evolutionary and Functional Annotations for Animal, Fungal, Plant, Archaeal, Bacterial and Viral Orthologs." *Nucleic Acids Research* 45 (D1): D744–49.

Zelkowski, Mateusz, Katarzyna Zelkowska, Udo Conrad, Susann Hesse, Inna Lermontova, Marek Marzec, Armin Meister, Andreas Houben, and Veit Schubert. 2019. "Arabidopsis NSE4 Proteins Act in Somatic Nuclei and Meiosis to Ensure Plant Viability and Fertility." *Frontiers in Plant Science* 10 (June): 774.

Zerbino, Daniel R., Premanand Achuthan, Wasiu Akanni, M. Ridwan Amode, Daniel Barrell, Jyothish Bhai, Konstantinos Billis, et al. 2018. "Ensembl 2018." *Nucleic Acids Research* 46 (D1): D754–61.

Manuscript (with diff)

Click here to access/download;Manuscript;resubmission_tracked_changes.do



Formatted Table

# Scalable Phylogenetic Profiling using MinHash Uncovers Likely Eukaryotic Sexual Reproduction Genes

**David Moi[1,2,3,*], Laurent Kilchoer[1,2,3], Pablo S. Aguilar[4,5] and Christophe Dessimoz[1,2,3,6,7,*]**

[1]Department of Computational Biology, University of Lausanne, Switzerland; [2]Center for Integrative Genomics, University of Lausanne, Switzerland; [3]SIB Swiss Institute of Bioinformatics, Lausanne, Switzerland; [4]Instituto de Investigaciones Biotecnologicas (IIBIO), Universidad Nacional de San Martín Buenos Aires, Argentina; [5]Instituto de Fisiología, Biología Molecular y Neurociencias (IFIBYNE-CONICET), [6]Department of Genetics, Evolution, and Environment, University College London, UK; [7]Department of Computer Science, University College London, UK.

*Corresponding authors: david.moi@unil.ch and christophe.dessimoz@unil.ch

## Abstract

Phylogenetic profiling is a computational method to predict genes involved in the same biological process by identifying protein families which tend to be jointly lost or retained across the tree of life. Phylogenetic profiling has customarily been more widely used with prokaryotes than eukaryotes, because the method is thought to require many diverse genomes. There are now many eukaryotic genomes available, but these are considerably larger, and typical phylogenetic profiling methods require at least quadratic time ~~or worse in~~ as a function of the number of genes. We introduce a fast, scalable phylogenetic profiling approach entitled HogProf,

which leverages hierarchical orthologous groups for the construction of large profiles and locality-sensitive hashing for efficient retrieval of similar profiles. We show that the approach outperforms Enhanced Phylogenetic Tree, a phylogeny-based method, and use the tool to reconstruct networks and query for interactors of the kinetochore complex as well as conserved proteins involved in sexual reproduction: Hap2, Spo11 and Gex1. HogProf enables large-scale phylogenetic profiling across the three domains of life, and will be useful to predict biological pathways among the hundreds of thousands of eukaryotic species that will become available in the coming few years. HogProf is available at https://github.com/DessimozLab/HogProf.

## Introduction

The NCBI Sequence Read Archive (SRA) contains $1.6x10^{16}$ nucleotide bases of data and the quantity of sequenced organisms keeps growing exponentially. To make sense of all of this new genomic information, annotation pipelines need to overcome speed and accuracy barriers. Even in a well-studied model organism such as *Arabidopsis thaliana*, nearly a quarter of all genes are not annotated with an informative gene ontology term ~~[1]~~ (Skunca, Altenhoff, and Dessimoz 2012; "TAIR - Portals - Genome Snapshot" n.d.). One way to infer the function of a gene product is to analyse the biological network it is involved in ~~and form a hypothesis based on its~~. Using guilt by association strategies it is possible to infer function based on physical or regulatory interactors.

Unfortunately, biological network inference is mostly limited to model organisms as well and genome scale data is only available through the use of noisy high-throughput experiments.

To ascribe biological functions to these new sequences, most of which originate from non-model organisms, computational methods are essential [reviewed in 2].(reviewed in Cozzetto and Jones 2017). Among the computational function prediction techniques that leverage the existing body of experimental data, one important but still underutilised approach in eukaryotes is *phylogenetic profiling [3]*.*(Pellegrini et al. 1999)*: positively correlated patterns of gene gains and losses across the tree of life are suggestive of genes involved in the same biological pathways.

Phylogenetic profiling has been more commonly performed on prokaryotic genomes than on eukaryotic ones. Perhaps due to the relative paucity of eukaryotic genomes in the 2000s, earlier benchmarking studies observed poorer performance in retrieving known interactions with eukaryotes than with Prokaryotes [4–6].(Snitkin et al. 2006; Jothi, Przytycka, and Aravind 2007; Ruano-Rubio, Poch, and Thompson 2009). The situation today is considerably different; the GOLD database [7](Mukherjee et al. 2017) tracks over 6000 eukaryotic genomes. Multiple successful applications of phylogenetic profiling in eukaryotes have been published in recent years, e.g.. For example, they have been used to infer small RNA pathway genes [8],(Tabach et al. 2013), the kinetochore network [9],(van Hooff et al. 2017), ciliary genes [10],(Nevers et al. 2017), or homologous recombination repair genes [11].(Sherill-Rofe et al. 2019).

Still, largeLarge-scale phylogenetic profiling with eukaryotes remains complex eukaryotic genomes is computationally challenging, because eukaryotic genomes are larger and more complex than their prokaryotic counterparts, and because since most state-of-the-art phylogenetic profiling

Formatted: Footer

methods typically scale at least quadratically with the number of gene families and linearly with the number of genomes. As a result, most mainstream phylogenomic databases, such as Ensembl [12],(Zerbino et al. 2018), EggNOG [13],(Huerta-Cepas et al. 2016), OrthoDB [14],(Zdobnov et al. 2017), or OMA [15](Altenhoff et al. 2018) do not provide phylogenetic profiles.

One available resource is STRING (Szklarczyk et al. 2017), a protein interaction focused database which integrates multiple channels of evidence to support each interaction. The inference of phylogeneticlinks between profiles STRING offers are obtained using large datasets is challenging. SomeSVD-phy (Franceschini et al. 2016) which represents profiles as bit-score distances between all proteins present in a given proteome and their closest homologues in all of the genomes included in the analysis. Dimensionality reduction is applied to the matrix to remove signal coming from the species tree and the profiles are clustered to infer interactions. In STRING, this is implemented with their set of 2031 organisms for which profile distance matrices are precalculated and incorporated into their network inference pipeline. Although this approach captures information on the distribution of extant distances, it does not reconstitute the evolutionary history of protein families and may lack information relative to duplication and loss events. Furthermore, as we show in the *Methods section*, the truncated Singular Value Decomposition approach does not scale well beyond a few genomes at a time.

To construct profiles representing groups of homologues, some pipelines resort to all-vs-all sequence similarity searches to derive orthologous groups and only count binary presence or absence of a member of each group in a limited number of genomes [16,17](Ta, Koskinen, and Holm 2011; Kensche et al. 2008) or forgo this step altogether and ignore the evolutionary history of each group of homologuesprotein family, relying instead on co-occurrence in extant genomes

[18].(Niu et al. 2017). Other tree-based methods infer the underlying evolutionary history from the presence of extant homologues [19]. In our pipeline, we leveraged the already existing OMA orthology inference algorithm, which has been benchmarked and integrates with other proteomic and genomic resources [15]. The OMA database describes the orthology relationships among all protein coding genes of over 2000 cellular organisms. One core object of this database is the Hierarchical Orthologous Group (HOG) [20].(Li et al. 2014). Each HOG contains all of the descendants of a single ancestor gene. When a gene is duplicated during its evolution, the paralogous genes and the descendants of the orthologue are contained in separate subhogs which describe their lineage back to their single ancestor gene (hence the hierarchical descriptor). A brief introductory video tutorial on HOGs is available at https://youtu.be/5p5x5gxzhZA.

Here, we introduce a scalable approach which combines the efficient generation of phylogeny-aware profiles from hierarchical orthologous groups with ultrafast retrieval of similar profiles using locality sensitive hashing. Furthermore, the approach leverages the properties of minhash signatures to allow for the selection of clade subsets and for clade weightings in the construction of profiles. The improvements in performance of our method make it possible to build profiles for the over 2000 genomes contained in OMA. We show that the method is as accurate as a state-of the art phylogeny-based method, and illustrate its usefulness by retrieving biologically relevant results for several genes of interest. Because the method is unaffected by the number of genomes included and scales logarithmically with the number of hierarchical orthologous groups added, it will efficiently perform with the exponentially growing number of eukaryotic genomes.

All of the code used to produce the results shown in this manuscript can be downloaded at https://github.com/DessimozLab/HogProf.

**Formatted:** Footer

## Results

In the following sections we first compare our profiling distance metric against other profile distances in order to characterize the Jaccard hash estimation's precision and recall characteristics. Following this quantification, we show our pipeline's capacity in reconstituting a well known interaction network as well as augmenting it with more putative interactors using its search functionality. Finally, to illustrate a typical use case of our tool, we explore a poorly characterized network.

A scalable phylogenetic profiling method using locality-sensitive hashing and hierarchical orthologous groups

Formatted: Normal, Line spacing: single

Most phylogenetic profiling methods consist of two steps: creating a profile for each homologous or orthologous group, and comparing profiles. When they were first implemented, profiles were constructed as binary vectors of presence and absence across species [3].(Pellegrini et al. 1999). Since then, variants have been proposed, which take continuous values [9] (van Hooff et al. 2017) such as alignment scores with the gene of a reference species [11] (Sherill-Rofe et al. 2019) or which count the number of paralogs present in each species. Yet other variants convey the number of events on branches of the species tree [6]. However, all approaches are limited with respect to the computational power required to cluster profiles using their respective distance metrics. Due to this computational cost, profiling efforts are typically focused on reconstructing pathways with known interactors using existing annotations and evidence rather than being used

~~as an exploratory tool to search for new interactors and reconstituting completely unknown networks.~~(Ruano-Rubio, Poch, and Thompson 2009).

In our pipeline, we leveraged the already existing OMA orthology inference algorithm to provide the input data to create our profiles. The OMA database describes the orthology relationships among all protein coding genes of currently 2288 cellular organisms (1674 bacteria, 152 archaea, and 462 eukaryotes). Within eukaryotes, OMA includes 188 animals, 135 fungi, 57 plants, and 82 protists and has been benchmarked and integrated with other proteomic and genomic resources (Altenhoff et al. 2018). One core object of this database is the Hierarchical Orthologous Group (HOG) (Altenhoff et al. 2013). Each HOG contains all of the descendants of a single ancestor gene. When a gene is duplicated during its evolution, the paralogous genes and the descendants of the orthologue are contained in separate subhogs which describe their lineage back to their single ancestor gene (hence the hierarchical descriptor). ~~In our approach to the problem of profiling, we captured the evolutionary history of each HOG in enhanced phylogenies and encode~~

We captured the evolutionary history of each HOG in enhanced phylogenies and encoded them in probabilistic data structures (Fig. 1). These are used to compile searchable databases to allow for the retrieval of coevolving HOGs with similar evolutionary histories and compare the similarity of two HOGs. The two major components of the pipeline that are responsible for constructing the enhanced phylogenies and calculating probabilistic data structures to represent them are pyHam ~~and Datasketch, respectively.~~(Train et al. 2018) and Datasketch ("Datasketch: Big Data Looks Small — Datasketch 1.0.0 Documentation" n.d.), respectively. Further details on the implementation are provided in the Methods section. The combination of these two tools now allows for the main

Formatted: Footer

innovation of our pipeline: the efficient exploration and clustering of profiles to study known and novel biological networks.

Currently, existing profiling pipelines are limited with respect to the computational power required to cluster profiles using their respective distance metrics. Due to this bottleneck, profiling efforts are typically focused on reconstructing pathways with known interactors using existing annotations and evidence rather than being used as an exploratory tool to search for new interactors and reconstituting completely unknown networks.

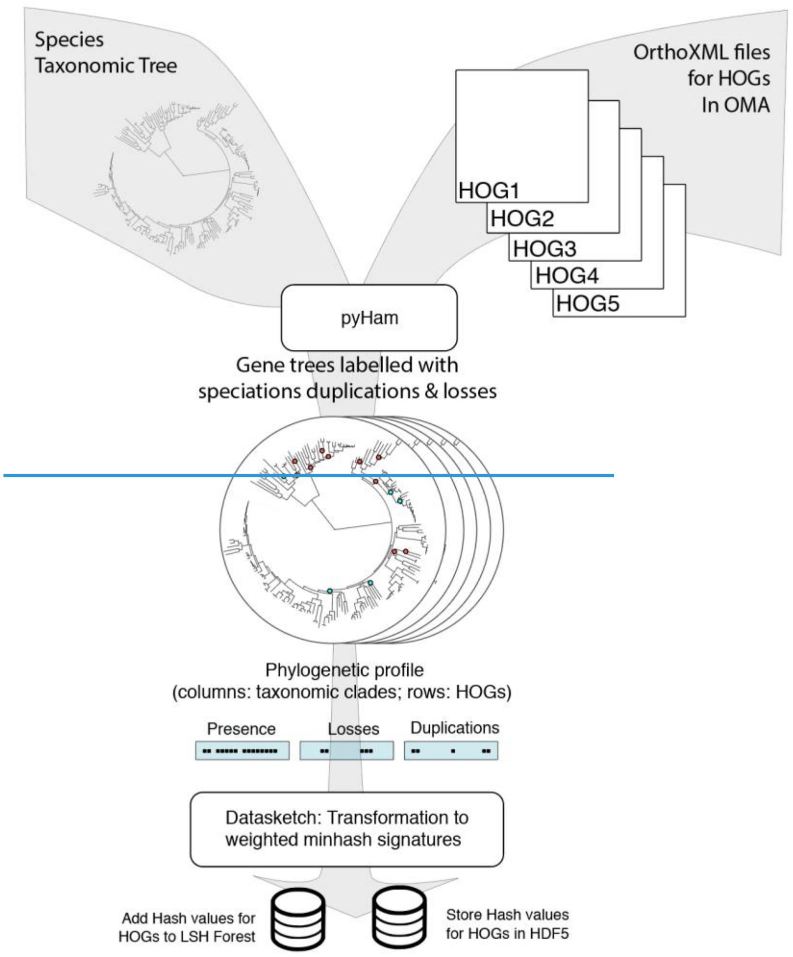


**Formatted Table**

**Formatted:** Footer

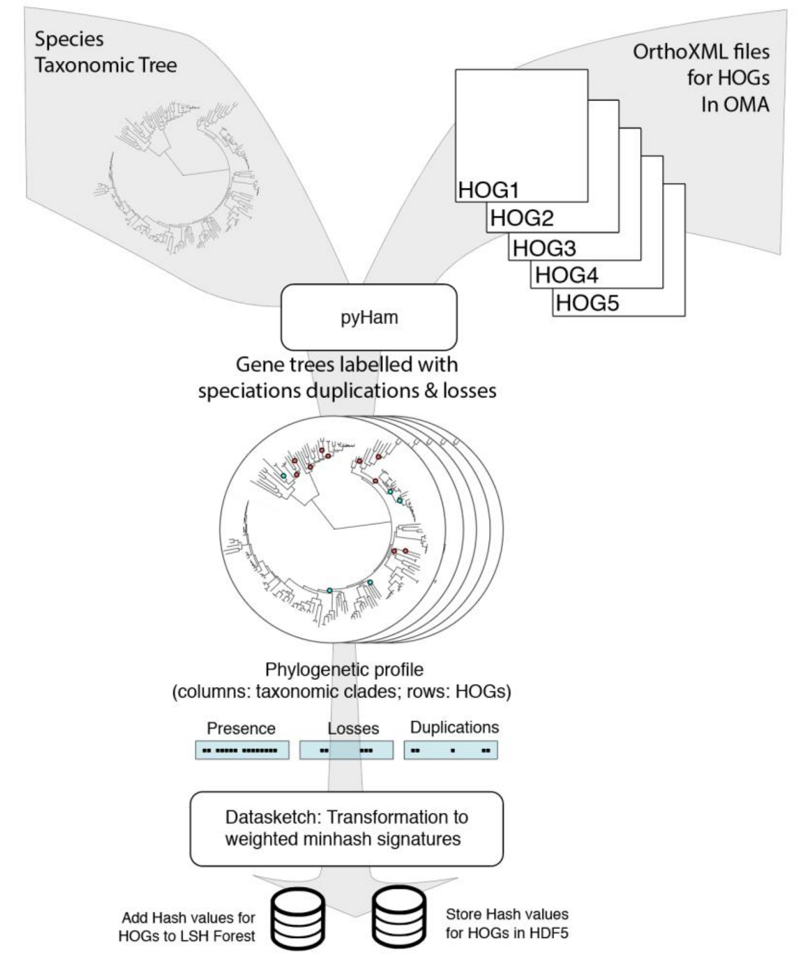


**Fig. 1. Diagram summarizing the different steps of the pipeline to generate the LSH Forest and hash signatures for each HOG.** The labelled phylogenetic trees generated by pyHam are converted into phylogenetic profiles and used to generate a weighted ~~minhash~~MinHash signature with Datasketch. The hash signatures are inserted into the LSH Forest and stored in an HDF5 file.

Formatted: Font: Bold

The tool we have created leverages the properties of MinHash signatures to allow for the selection of clade subsets and for clade weightings in the construction of profiles and make it possible to build profiles with the complete set of genomes contained in OMA. We show that the method outperforms other phylogeny-based methods (Ta, Koskinen, and Holm 2011; Glazko and Mushegian 2004; Ranea et al. 2007), and illustrate its usefulness by retrieving biologically relevant results for several genes of interest. Because the method is unaffected by the number of genomes included and scales logarithmically with the number of hierarchical orthologous groups added, it will efficiently perform with the exponentially growing number of genomes as they become available.

The code used to generate the results in this manuscript are available at https://github.com/DessimozLab/HogProf.

## Results

In the following sections we first compare our profiling distance metric against other profile distances in order to characterize the Jaccard hash estimation's precision and recall characteristics. Following this quantification, we show our pipeline's capacity in reconstituting a well known interaction network as well as augmenting it with more putative interactors using its search functionality. Finally, to illustrate a typical use case of our tool, we explore a poorly characterized network.

Formatted: Footer

**Accuracy of predicted phylogenetic profiles in an empirical benchmark**

We compared the performance of our profiling metric to existing profile distances using benchmarking data available in Ta *et al.* [16].(2011). In that benchmark, the true positive protein-protein interactions (PPIs) were constructed using data available from CORUM [21] and the MIPS [22](Giurgiu et al. 2018) and the MIPS (Mewes et al. 2004) databases for the human and yeast interaction datasets. True negatives were constructed by mixing proteins known to be involved in different complexes. The dataset is balanced with 50% positive and 50% negative samples. Using their Uniprot identifiers, these interaction pairs were mapped to their respective HOGs and their profiles were compared using the hash based Jaccard score estimate. The comparison below shows HogProf alongside other profiling distance metrics that are considerably more computationally intensive, including the Enhanced Phylogenetic Tree (EPT) metric shown in Ta *et al.* [16].(2011). Yet, our approach outperformed these previous methods, yielding the highest Area Under the Curve for both yeast and human datasets (Figure 2, Table 1).

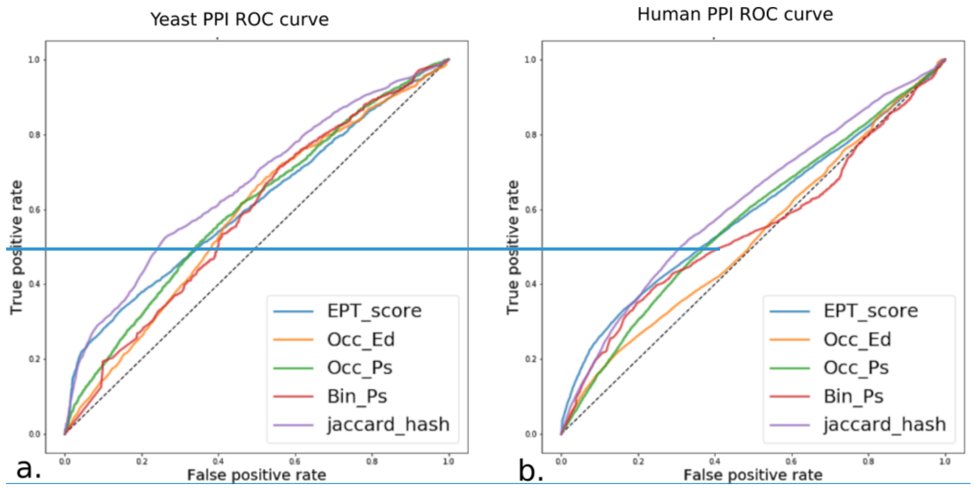


Formatted Table

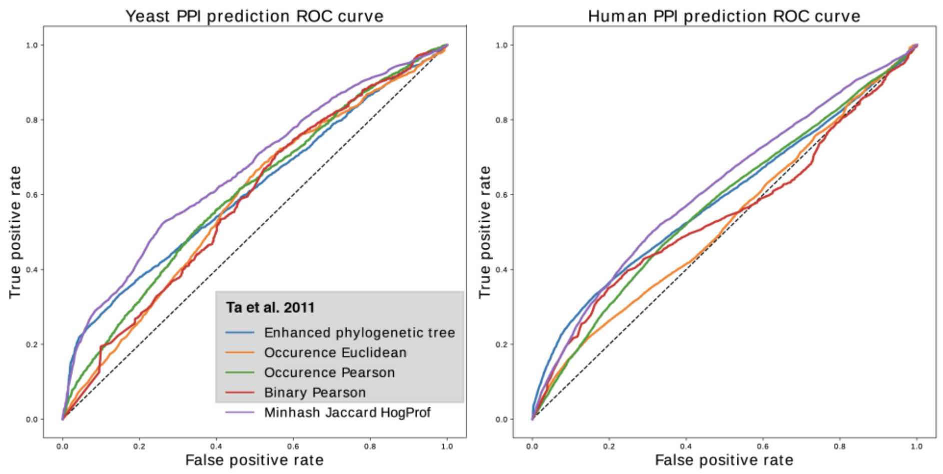


**Fig. 2. ROC curves for all profiling methods**. **a.** Yeast protein-protein interactions. ~~Jaccard Hash and Jaccard Hash Opt perform better than all metrics~~Our method (MinHash Jaccard HogProf), performs best overall, but when high precision is required, ~~EPT score~~Enhanced phylogenetic Tree (Ta, Koskinen, and Holm 2011) is still slightly more accurate. **b.** Human protein-protein interactions. Jaccard Hash ~~and Jaccard Hash Opt perform~~HogProf performs better than all metrics overall but again, when high precision is required, EPT score is still slightly more accurate. ~~In both subfigures, Jaccard hash refers to the profiles containing all clades with all weights for each event and taxonomic level set to 1. "EPT_score" refers to the Enhanced Phylogenetic Tree metric developed in [16]. "Bin_Ps"~~Binary Pearson refers to a distance using binary vectors and Pearson correlation described in ~~[23]. "Occ_Ed" and "Occ_Ps"~~(Glazko and Mushegian 2004). Occurence Euclidean and Occurence Pearson refer to the occurence profiles with Euclidean distance and Pearson correlation as described in ~~[24].~~(Ranea et al. 2007).

Formatted: Footer

**Table 1. AUC values for Profiling distance metrics.**

| Metric | AUC Yeast | AUC Human |
|---|---|---|
| Jaccard Hash | 0.6634 | 0.6155 |
| EPT | 0.6104 | 0.5875 |
| BIN PS | 0.5840 | 0.5463 |
| OCC ED | 0.5829 | 0.5268 |
| OCC PS | 0.6028 | 0.5714 |

**Formatted Table**

**Formatted:** Font: 10 pt

**Formatted:** Font: 10 pt

**Formatted:** Font: 10 pt

**Formatted:** Font: 10 pt

**Formatted:** Font: 10 pt

**Recovery of a canonical network: the kinetochore network**

To further validate our profiling approach on a known biological network, we used our pipeline to replicate previous work shown in van Hooff et al. ~~[9].~~(2017). Their analysis focuses on the evolutionary dynamics of the kinetochore complex, a microtubule organizing structure that was present in the last eukaryotic common ancestor (LECA) and has undergone many modifications throughout evolution in each eukaryotic clade where it is found. Its modular organization has allowed for clade-specific additions or deletions of modules to the core complex which remains relatively stable. This modular organisation and clade-specific emergence of certain parts of the complex make it an ideal target for phylogenetic profiling analysis.

We show that our ~~minhash~~MinHash signature comparisons are also capable of recovering the kinetochore complex organisation. After considering just the HOGs for the families used in van Hooff et al. ~~[9],~~(van Hooff et al. 2017), we augmented their set of profiles using ~~the~~ LSH Forest (Bawa, Condie, and Ganesan 2005) to retrieve interactors that may also be involved in the kinetochore (and the also included anaphase promoting complex (APC~~) network~~)) networks which

have not been ~~detected~~cataloged by these authors. ~~By using a well-studied network in eukaryotes to test the LSH Forest search, we can rely on previous work and annotations to quantify the quality of the returned results.~~ Using the Gene Ontology (GO) terms ~~[25]~~(Ashburner et al. 2000) of all proteins returned in our searches for novel interactors, we ~~quantify how enriched they are for the~~were able to identify proteins with specific functions we would expect to be related to our network of interest.

In their work, van Hooff et al. ~~[9]~~(van Hooff et al. 2017) used pairwise Pearson correlation coefficients between the presence and absence vectors of the various ~~protein~~kinetochore components ~~of~~to recompose the ~~kinetochore in the 90~~ organisation of the complex. Their profiles were constructed using the proteomes of a manually selected set of 90 organisms ~~as a distance metric between a~~with manually curated ~~set of~~ profiles corresponding to each component of the complex. ~~Using this pairwise comparison of all vectors~~After establishing a distance kernel, they clustered the profiles and were able to recover known sub-~~componenents~~components of the complex using just evolutionary information. Using our hash-based Jaccard distance metric in an all-vs-all comparison between the HOGs corresponding to each of these protein families, we were also able to recover the main modules of the kinetochore complex with a similar organisation to the one defined by van Hooff et al. The color clustering in ~~figure~~Figure 3 corresponds to their original manual definition of these different subcomplex modules~~ based. Despite the vastly different methods used in the construction and comparison of the profiles used to recover the network in both pipelines, we~~. We observe that the distance matrices generated by each profiling approach are correlated ~~and are recovering similar evolutionary signals, (~~with Spearman correlation

**Formatted:** Footer

of 0.26268 (p < 1e-100) and Pearson correlation of 0.35364 (p < 1e-100).) ) and are recovering similar evolutionary signals despite their construction using different methods.

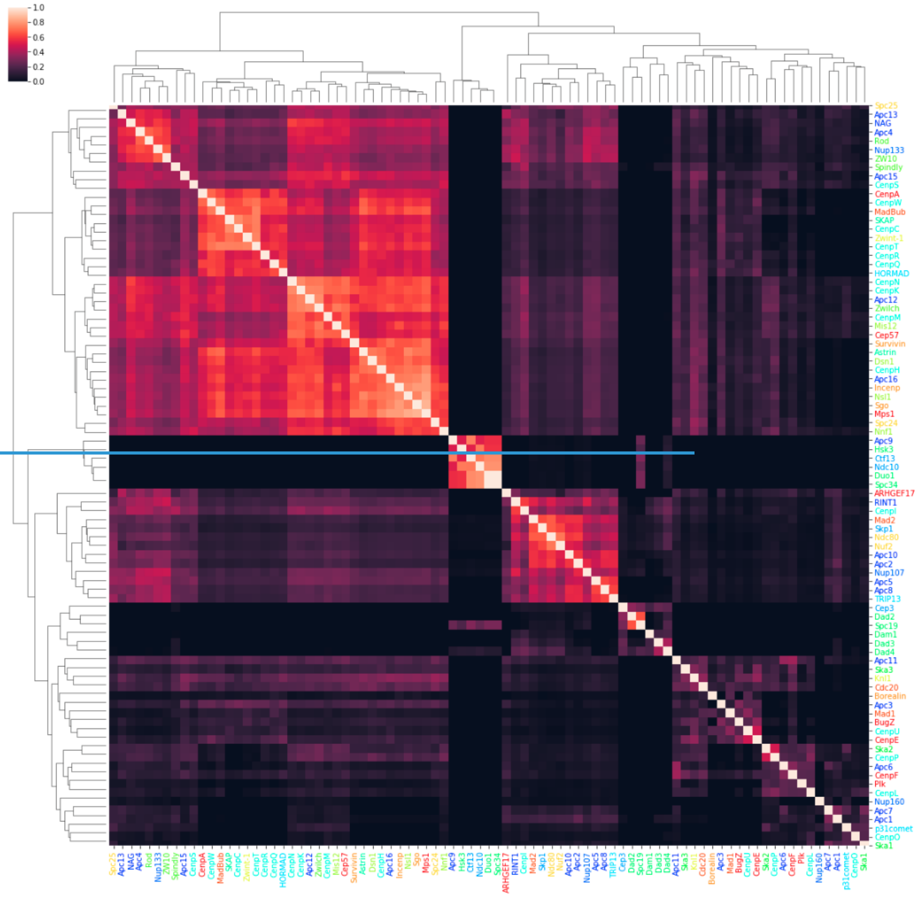


**Formatted Table**

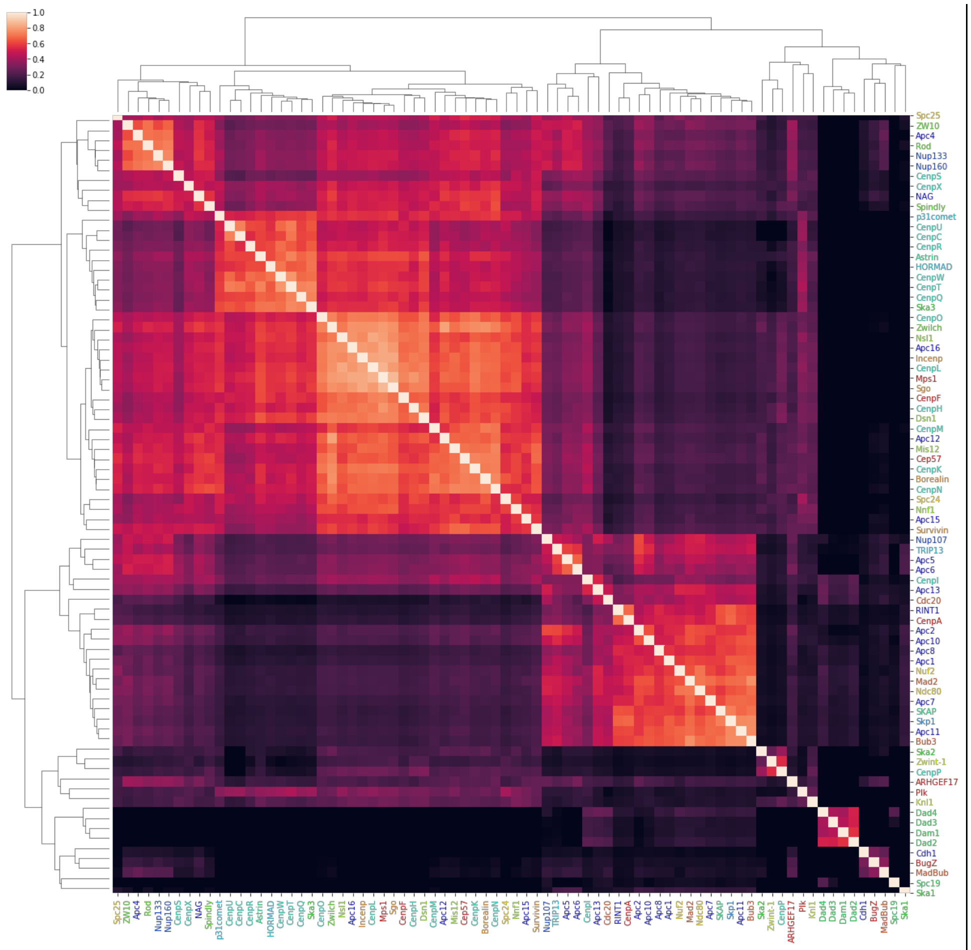


**Fig. 3. Recovery of kinetochore and APC complexes.** After mapping each of the protein families presented in Van Hooff et al. ~~[9]~~(van Hooff et al. 2017) to their corresponding HOG, a distance matrix was constructed by comparing the Jaccard hash distance between profiles using HogProf. Name colors in the rows and columns of the matrix correspond to the kinetochore and APC subcomplex components as defined manually using literature sources ~~in van Hooff et~~

**Formatted:** Footer

al. All code used to construct the figure is available on the HogProf repository.(van Hooff et al. 2017).

Formatted: Font: Bold

The All-vs-All comparison of the profiles revealsrevealed several well defined clusters in both worksstudies including the Dam-Dad-Spc19 and CenP subcomplexes. However, ourUnlike the Van Hoof er al. approach, HogProf profiles were constructed alongside all other HOGs in OMA and were not curated before being compared. With only the initial information of which proteins were in the complex, we mapped them to their corresponding OMA HOGs and, with this example, demonstrated the ability to reconstruct any network of interest or construct putative networks using the search functionality of our pipeline with minimal computing time. However, itIt should be noted that the quality of the OMA HOGs used to construct the enhanced phylogenies and hash signatures directly influences our ability to recover complex organisation.

To illustrate the utility of the search functionality of our tool, we used the profiles known to be associated with the kinetochore complex to search for other interactors. All HOGs corresponding to the protein families used to analyse the kinetochore evolutionary dynamics in van Hooff et al. [9](van Hooff et al. 2017) were used as queries against an LSH Forest containing all HOGs in OMA. By performing an all-vs-all comparison of the minhash signatures of the queries and returned results, a Jaccard distance matrix was generated showing potential functional modules associated with each known component of the kinetochore and APC complexes.

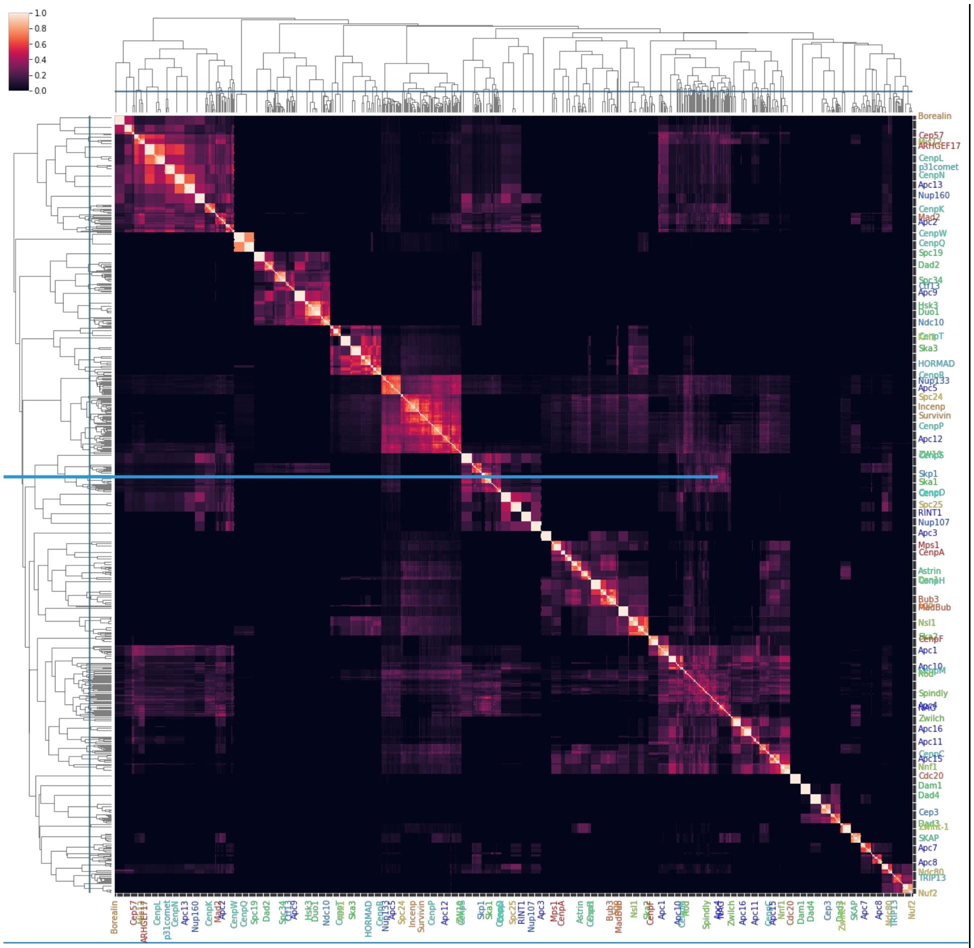


**Formatted Table**

**Formatted:** Footer

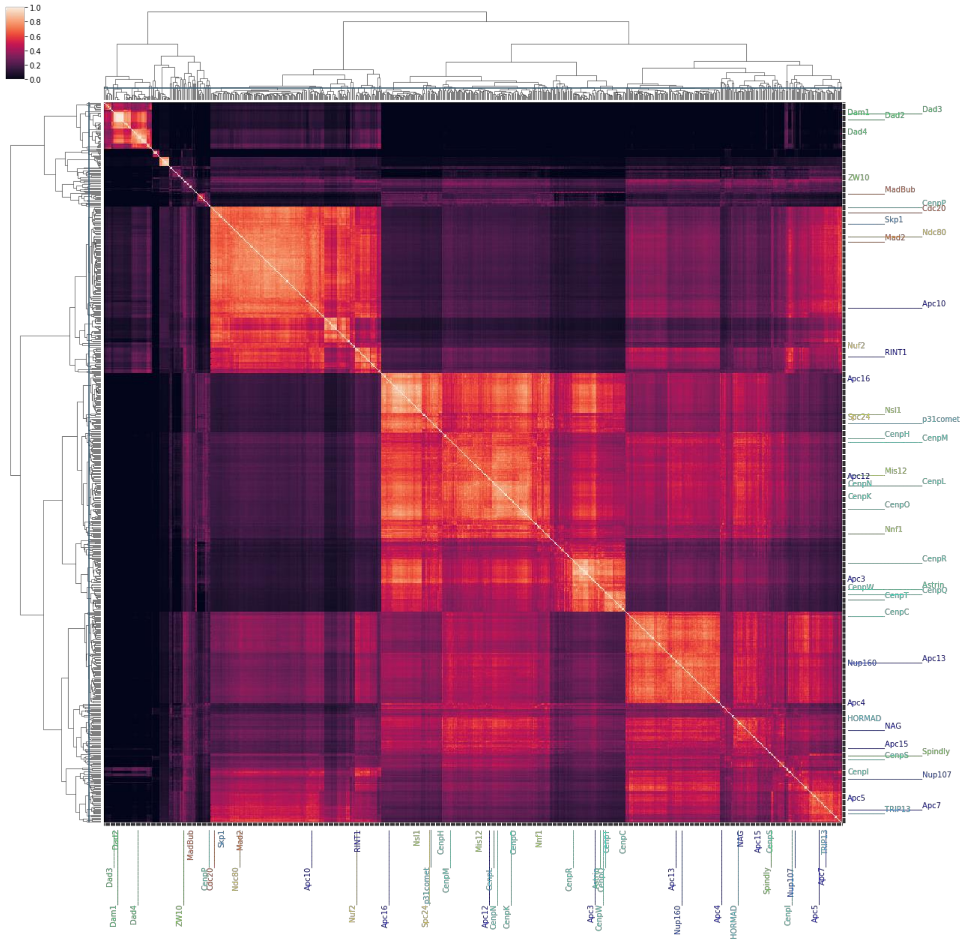


**Fig. 4. Putative novel components of the kinetochore and APC complexes.** The profiles associated with all HOGs mapping to known kinetochore components shown in Figure 3 were used to search the LSH Forest and retrieve the top 10 closest coevolving HOGs resulting in a list of 871 HOGs including the queries from the original complexes. The Jaccard distance matrix is shown between the hash signatures of all query and result HOGs. UPGMA clustering was

applied to the distance matrix rows and columns. Labelled rows and columns correspond to profiles from the starting kinetochore dataset ~~[9].~~(van Hooff et al. 2017). A cutoff hierarchical clustering distance of 1.3 was manually chosen (blue lines) ~~was used~~ to ~~generate~~limit the maximum cluster size to less than 50 HOGs. This cutoff resulted in a total of ~~136~~142 clusters of HOGs used for GO enrichment to identify functional modules. The coloring of the protein family names to the right and below the matrix is identical to the complex related coloring shown in Figure 3. ~~All code used to construct this figure is available in the HogProf repository.~~

**Formatted:** Font: Bold

To verify that the results returned by our search were not spurious, we performed GO enrichment analysis of the returned HOGs that were not part of the original set of queries but appeared to be coevolving closely with known kinetochore components. Given the incomplete nature of ~~Gene Ontology annotations ["open world assumption", 26],~~GO annotations ("open world assumption", Dessimoz, Škunca, and Thomas 2013), many of these proteins may actually be involved in the kinetochore interaction network but this biological function could be still undiscovered. However, even with this limitation, salient annotations relevant to the kinetochore network were returned in the search results (Table 2 and Supplementary Data 1). The identifiers of all protein sequences contained in the HOGs returned by the search results were compiled and the GO enrichment of each cluster shown in Figure 4 was calculated using the OMA annotation corpus as a background. The enrichment results were manually parsed and salient annotations related to HOGs were selected to be reviewed further in the associated literature to check for the association of the search result with the query HOG (Table 2).

**Formatted:** Footer

**Table 2. Manually curated biologically relevant search results for interactors coevolving with van Hooff *et al.*'s kinetochore and APC selected protein families** [9]. Notable protein(van Hooff et al. 2017). Protein families (Result) returned within clusters containing query HOGs (Cluster) are listed with their pertinent annotation and literature. GO enrichment results of clusters that contained one or more queries from the original kinetochore network were analyzed manually. We searched for literature supporting the relevant GO annotations, thereby confirming that the results returned by the LSH were associated with kinetochore and APC processes. This is a non-exhaustive summary of some selected results. The full enrichment results are available as Supplementary Data 1.

| Cluster | Result | GO Term | Citation |
|---|---|---|---|
| APC1 | CFAP157 | GO:0035082 axoneme assembly | [27](Weidemann et al. 2016) |
| APC12HOMRAD | BRWD1C2CD3 | GO:0007010 cytoskeleton organizationGO:0061511 centriole elongation | [28](Thauvin-Robinet et al. 2014) |
| CenpQAPC12 | CDC26ESCO2 | GO:0007346 regulation of mitotic cell cycleGO:0007059 chromosome segregation | [29](Lu et al. 2017) |
| KNL1 | TACC3 | GO:0007091 metaphase/anaphase transition of mitotic cell cycle | [30](Cheeseman et al. 2013) |

For instance, TACC3, a known physical interactor of the kinetochore complex and important regulator of the kinetochore tension [30]was found by our search. Another example is CFAP157,

**Formatted:** Indent: Left: 0.5"

**Formatted Table**

**Formatted:** Justified, Space After: 10 pt, Line spacing: 1.5 lines

a cilia and flagella associated protein which may seem like an unlikely interactor with the APC. However, it has previously been shown that the APC activity regulates ciliary length unstabilizing axonemal microtubules [31]. Thus, CFAP157 might be involved in recruitment of APC regulators (such as Cdc20) or ciliary kinases (such as Nek1) both known to mediate APC regulation of ciliary dynamics [31] .For instance, our search identified TACC3, which is known to be part of a structural stabilizer of kinetochore microtubules tension although it does not directly interact with the kinetochore complex (Cheeseman et al. 2013). ESCO2, a cohesin N-acetyltransferase needed for proper chromosome segregation during meiosis also plays a role in kinetochore-microtubule attachments regulation during meiosis (Lu et al. 2017). While these results are certainly promising, many of the unannotated proteins returned by our search likely contain more regulatory, metabolic and physical interactors which may prove to be interesting experimental targets. The diverse types of interactions detected by our pipeline are discussed further in the discussion section.

**Search for a novel network**

When studying networks with a lack of annotation and experimental characterization, it is difficult to quantify the relevance of retrieved search results. In typicalTypical research use cases involvingfor profiling often involve uncharacterized protein families inacting within poorly studied neworks, this will often be the case.. In this section we present the search results for three HOG queriesHOGs known to be involved in the processes of meiosis, syngamy and karyogamy. These three major events occur in almost all sexually reproducing eukaryotes during their reproductive cycle. Despite the ubiquitous nature of sex and its probable presence in LECA [32],(Speijer, Lukeš, and Eliáš 2015), the protein networks involved in each part of these processes are very poorly

Formatted: Footer

understood andhave limited experimental data is available, even in model organisms. However, someSome key protein families involved in these biological processeseach step are known to have evolutionary patterns indicating an ancestral sequence in the LECA with subsequent modifications and losses [32].(Speijer, Lukeš, and Eliáš 2015). The three following sections detail the returned results of the phylogenetic profiling pipeline with the Hap2, Gex1 and Spo11 families which all share this evolutionary pattern and are known to be critical for the process of gamete fusion, nuclear fusion and meiotic recombination, respectively. As in section 3.2 we also used GO enrichment to quantify the relevance of the returned search results. The proteins contained in the top 100 HOGs returned by the LSH Forest were analyzed for GO enrichment using all OMA annotations as a background. Due to the presence of biases in the GO annotation corpus [33](Altenhoff et al. 2012) we have also chosen to show the number of proteins annotated with each biological process selected from the enrichment out of the total number of annotated proteins.

***Query with Hap2***

The Hap2 protein family has been shown to catalyze gamete membrane fusion in many eukaryotic clades [34,35]. It has a particularly spotty pattern of presence and absence on the taxonomic tree despite its phylogeny supporting the hypothesis of vertical descent from LECA. This protein family is known to be highly divergent in amino acid sequence despite its conserved fold and shares structural homology with viral and somatic membrane fusion proteins [35-37]. The HOG containing Hap2 in OMA only contains the eukaryotic gamete fusion protein subfamily of this structural superfamily. Part(Liu et al. 2008; Valansi et al. 2017; Fédry et al. 2017; Feng, Dong, and

Springer 2018). A subset of the GO enrichment of the search results for the top 100 coevolving HOGs are shown below in ~~table~~Table 3.

**Table 3. Manually curated biologically relevant enriched GO terms from returned results.** The ~~chosen input protein~~query sequence ~~for~~ Hap2 is ~~that of~~ UniProt entry F4JP36 ~~and the corresponding~~with OMA ~~entry~~identifier ARATH26614 belonging to OMA HOG:0406399. The full enrichment results are available in the Supplementary Data 2.

| Term | Biological process | P-value | N-proteins |
|---|---|---|---|
| GO:0006338 | chromatin remodeling | 9.72e-54 | 61/3426 |
| GO:0048653 | anther development | 1.69e-35 | 17/3426 |
| GO:0009793 | embryo development ending in seed dormancy | 2.88e-13 | 15/3426 |
| GO:0051301 | cell division | 6.88e-16 | 5/3426 |

~~Widely conserved sequences not belonging to the Hap2 HOG and found in coevolving HOGs were linked to gamete development and reproductive structure development (Table 3) [38,39]. This mirrors the initial discovery of Hap2, which was first found in angiosperms and linked to pollen tube guidance before the double fertilization event. Since Mendel, extensive work has been carried out describing reproductive processes in plants. Therefore, it is expected that the corpus of available annotations would be biased for annotations related to plant reproductive processes. The Hap2 HOG also appears to be coevolving with HOGs related to chromatin remodeling, an~~

**Formatted Table**

**Formatted:** Footer

important part of the reproductive process during gamete generation, and also post fusion, after the zygote cell is formed.

One particular family of interest which was returned in our search results is already characterized in angiosperms: LFR or leaf and flower related [40].(Wang et al. 2012). This protein family is required for the development of reproductive structures in flowers and serves as a master regulator of the expression of many reproduction related genes, but its role in lower eukaryotes remains undescribed despite its broad evolutionary conservation. Experiments targeting LFR's potential regulation of Hap2 expression may provide insight into how the fusion process is transcriptionally controlled in gametes across many eukaryotes despite their distinct reproductive strategies.

***Query with Gex1***

Gex1 has been shown to be involved inThe nuclear fusion ("karyogamy") andprotein Gex1 is present in many of the same clades as Hap2, with a similar spotty pattern of absence across eukaryotes and a phylogeny indicating a vertical descent from LECA [41]. (Ning et al. 2013). A subset of the GO enrichment of the search results for the top 100 coevolving HOGs shows the predictive potential of HogProf (tableare shown below in Table 4)..

**Table 4. Manually curated biologically relevant enriched GO terms from returned results.** The input proteinquery sequence chosen for Gex1 is based on the UniProt identifier Q681K7 and the correspondingwith OMA identifier ARATH38809 belonging to OMA HOG:0416115. The full enrichment results are available as Supplementary Data 3.

**Formatted:** Font: Bold

| GO Term | P-value | N-Proteins |
|---|---|---|
| GO:0042753 positive regulation of circadian rhythm | 2.12e-285 | 113/2685 |
| GO:0048364 root development | 7.81e-125 | 70/2685 |
| GO:0051726 regulation of cell cycle | 1.22e-92 | 99/2685 |
| GO:0000712 resolution of meiotic recombination intermediates | 1.65e-47 | 26/2685 |
| GO:0007140 male meiotic nuclear division | 1.19e-39 | 26/2685 |
| GO:0009553 embryo sac development | 1.43e-28 | 17/2685 |
| GO:0022619 generative cell differentiation | 3.59e-18 | 5/2685 |

In many sexually reproducing organisms, karyogamy is followed by restarting of the cell cycle. Our results strongly suggest that HOGs related to the restarting of the cell cycle have coevolved with Gex1 (Table 4). Again we find many angiosperm specific annotations due to the prior work in the study of their sexual reproduction. As was the case for Hap2, the taxonomic spread of the HOGs found in this search is broader than just angiosperms. Gex1 has alsoGex1 has been shown to be involved in gamete development and embryogenesis [42](Alandete-Saez et al. 2011) and therefore GO terms 0022619 and 0009553 are applied to this protein. Thus proteins that HogProf identified as putativeco-evolving with Gex1 interactorsand sharing these GO terms indicates thecan be considered potential relevance of these search resultsGex1 interactors.

One search result of particular interest is a protein family which goes by the lyrical name of parting dancers (PTD). PTD belongs to a family that has been characterized in *Arabidopsis thaliana* and budding and fission yeast, and is known to be required in reciprocal homologous recombination induring meiosis and localizes to the nucleus [43].(Wijeratne et al. 2006). Our search shows that

**Formatted Table**

**Formatted:** Font: Italic

**Formatted:** Footer

Gex1 coevolvedco-evolved closely with PTD, a protein known to be involved in preparing genetic material for its eventual merger with another cell's nucleus.

***Query with Spo11***

The Spo11 is a helicase that has been shown to beis involved in meiosis by catalyzing DNA double stranded breaks (DSBs) triggering homologous recombination. Spo11 is highly conserved throughout eukaryotes and homologues are present in almost all clades [44]. The(Keeney, Giroux, and Kleckner 1997). A subset of the GO enrichment of the search results for the top 100 coevolving HOGs are shown below in tableTable 5.

**Table 5. Manually curated biologically relevant enriched GO terms from returned results.** The chosen input proteinquery sequence for Spo11-1 is based on the UniProt identifier Q9M4A2 and the correspondingwith OMA identifier ARATH19148 belonging to OMA HOG:0605395. The full enrichment results are available in Supplementary Data 4.

| GO Term | P-value | N-Proteins |
|---|---|---|
| GO:0000737 DNA catabolic process, endonucleolytic | 0.00e+00 | 415/20562 |
| GO:0043137 DNA replication, removal of RNA primer | 0.00e+00 | 353/20562 |
| GO:0006275 regulation of DNA replication | 0.00e+00 | 552/20562 |
| GO:0006302 double-strand break repair | 8.11e-242 | 285/20562 |
| GO:0007292 female gamete generation | 2.71e-184 | 136/20562 |
| GO:0022414 reproductive process | 1.66e-93 | 127/20562 |

The process of chromosome recombination is one of the crucial steps in the generation of gametes and happens during meiotic prophase I when homologous chromosomes are paired and form the synaptonemal complex. It is encouraging to find that Spo11, the trigger of meiotic DSBs, has coevolvedco-evolved with other families involved in the inverse process of repairing the DSBs and finishing the process

Formatted Table
Formatted: Font: 11 pt
Formatted: Font: 11 pt
Formatted: Font: 11 pt
Formatted: Font: 11 pt
Formatted: Font: 11 pt
Formatted: Font: 11 pt
Formatted: Font: 11 pt
Formatted: Font: 11 pt
Formatted: Font: 11 pt
Formatted: Font: 11 pt
Formatted: Font: 11 pt
Formatted: Font: 11 pt
Formatted: Font: 11 pt
Formatted: Font: 11 pt
Formatted: Font: 11 pt
Formatted: Font: 11 pt
Formatted: Font: 11 pt

of recombination (Table 5). Other identified HOGs contain annotations such as gamete generation and reproduction also focusing ~~at~~on processes that result in cellular commitment to a gamete cell fate through meiosis. Proliferating cell nuclear antigen or PCNA ~~[45]~~(Strzalka and Ziemienowicz 2011) was also retrieved by our search. This ubiquitous protein family is an auxiliary scaffold protein to the DNA polymerase and recruits other interactors to the polymerase complex to repair damaged DNA, making it an interesting candidate for a potential physical interactor with Spo11.

~~In summary, this HogProf search focused on three proteins involved in sexual reproduction yielded a list of promising candidate proteins.~~

### *A broader search for the reproductive network*

A more in-depth treatment of the evolutionary conservation of gamete cell fate commitment and mating is available in previous publications ~~[32,41,46–50].~~(Malik et al. 2007; Loidl 2016; Speijer, Lukeš, and Eliáš 2015; Ning et al. 2013; Schurko and Logsdon 2008; Niklas, Cobb, and Kutschera 2014; Goodenough and Heitman 2014). Using these sources, a list of broadly conserved protein families known to be involved in sexual reproduction were compiled to be used as HOG queries to the LSH Forest to retrieve the top 10 closest coevolving HOGs. The hash signatures of the queries and results were compiled and used in an all-vs-all comparison to generate a Jaccard distance matrix.

**Formatted:** Font: 11 pt

**Formatted:** Font: 11 pt

**Formatted:** Font: 11 pt

**Formatted:** Font: 11 pt

**Formatted:** Font: 11 pt

**Formatted:** Font: 11 pt

**Formatted:** Font: 11 pt

**Formatted:** Font: 11 pt

**Formatted:** Font: 11 pt

**Formatted:** Font: 11 pt

**Formatted:** Footer

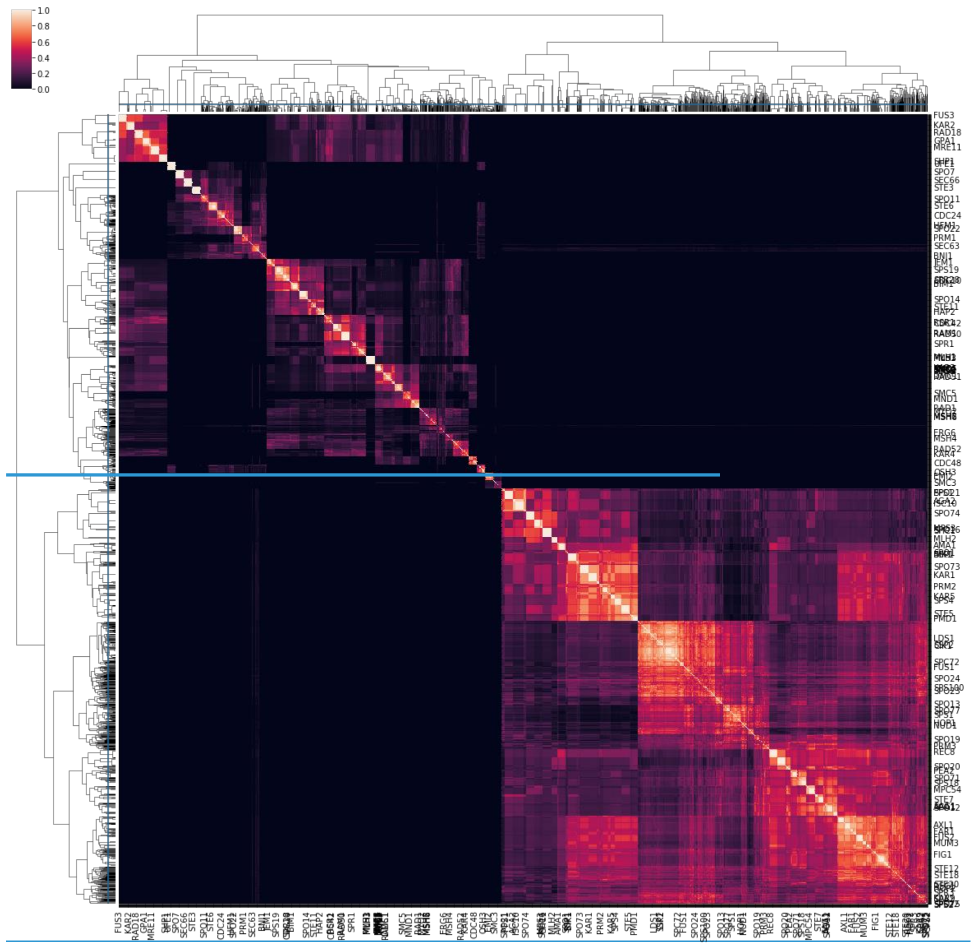


Formatted Table

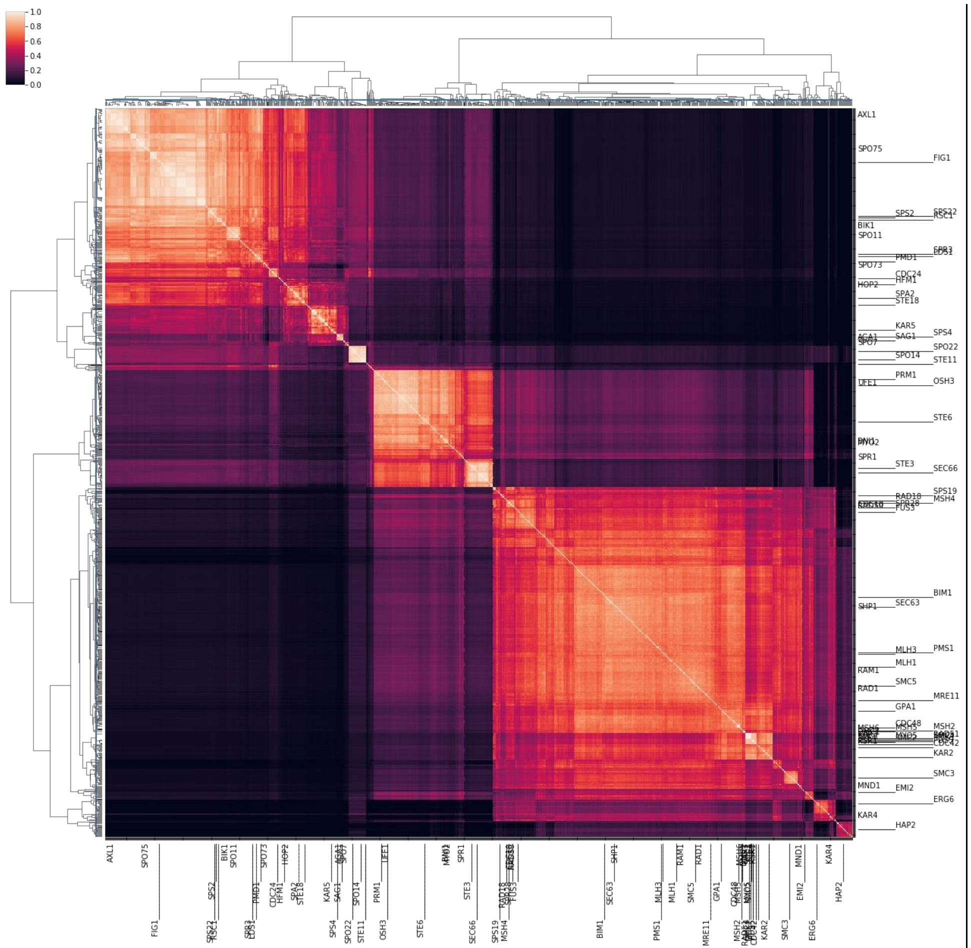


**Fig. 5. HogProf's reproductive network.** A list of proteins known to be involved in conserved sexual reproduction biological processes was compiled and each protein family was mapped to its HOG andOMA HOGs. These queries were used to search for the 20 closest coevolving HOGs in an LSH forest containing all HOGs in OMA. Each row and column of the Jaccard distance matrix corresponds to a HOG containing known sexual reproduction pathway protein

**Formatted:** Footer

~~families or a HOG returned by the search.~~ A Jaccard ~~distance matrix~~kernel was generated by performing an All vs All comparison of the Hash signatures of ~~the~~search results and queries. UPGMA clustering was performed on the rows and columns ~~to organize the HOGs into functional modules. The initial set of 121 protein sequences was augmented using the search functionality of the LSH by adding the top 20 closest returned HOGs resulting in a total of 2041 HOGs including the queries.~~of the kernel. A cutoff distance of .995 ( blue lines ) was ~~used~~manually chosen to ~~generate~~limit cluster sizes to less than 50 HOGs. This generated a total of 215 clusters of HOGs ~~(blue lines). The labels correspond to the names of the proteins used to generate the.~~ Names for queries~~. HOG names~~ are shown ~~correspond to the yeast~~with *Saccharomyces cerevisiae* gene names (~~-~~apart from Hap2 which is not present in fungi ).~~This nomenclature was chosen due to the large body of work related to the yeast pheromone response and mating pathways. The HOGs returned by our search are not labelled on the distance matrix. All code used to construct this figures is available on the HogProf repository.~~

The all-vs-all comparison of the Jaccard distances between these returned HOGs reveals clusters of putative interactors ~~coevolving~~co-evolving closely with specific parts of the sexual reproduction network. ~~The~~ Manual analysis of GO enrichment ~~of sequences within each cluster was analyzed manually and~~results revealed several ~~annotations related to~~ sexual reproduction ~~were found. These~~-related proteins which are summarized in Table 6 ~~after a manual curation and literature review as done in table 2 for the kinetochore search results.~~. In addition to annotated protein sequences and HOGs, many unannotated, coevolving HOGs ~~where found. Again, these may prove~~

to be useful experimental targets to answer open questions on the mechanisms behind sexual reproduction. were found.

Particularly for biological processes as complex and evolutionarily diverse as sexual reproduction, Gene OntologyGO annotations are, unsurprisingly, incomplete. Fortunately, our profiling approach is successful in identifying protein families with similar evolutionary patterns that have already been characterised and are directly relevant to sexual reproduction (Table 6). By considering the uncharacterized or poorly characterized families at the sequence and structure level, we may be able to predict their functions and reconstitute their local interactome. Our ultimate goal is to guide *in vivo* experiments to test and characterize these targets within the broader context of eukaryotic sexual reproduction.

Formatted: Font: Not Bold

**Table 6. Manually curated biologically relevant putative interactors from sexual reproduction search results.** Notable proteinProtein families (Result) within clusters containing query HOGs (Cluster) are listed with their pertinent annotation and literature. GO enrichment results of clusters that containedcontaining one or more queries from our list of queries were analyzed manually. We searched for literature associated to the relevant GO annotations confirming results returned by the LSH that were associated with sexual reproduction. This is a non-exhaustive summary of some salient returned results. The fullFull enrichment results are available in the Supplementary Data 5.

| Cluster | Result | GO Term | Citation |
|---|---|---|---|
| REC8 | NSE4 | GO:0030915 Smc5-Smc6 complex | [51](Zelkowski et al. 2019) |
| SPC72 | MID2 | GO:0000767 cell morphogenesis involved in conjugation | [52](Rajavel et al. 1999) |

Formatted Table

Formatted: Footer

| | | | |
|---|---|---|---|
| SPO71 | LES2 | GO:0031011 Ino80 complex | [53,54](Serber et al. 2016; Bao and Shen 2011) |
| SHC1, SPO16 | POG1 | GO:0000321 re-entry into mitotic cell cycle after pheromone arrest | [55,56](Leza and Elion 1999; van Werven et al. 2012) |

This example related to the ancestral sexual reproduction network illustrates the utility of the LSH Forest search functionality and OMA resources in exploratory characterization of poorly described networks. The interactions presented above in( Table 6 ) only represent our limited effort to manually review literature to highlight potentially credible interactions detected by our pipeline. Again, as was the case with our kinetochore and APC related searches, several interactions might not appear obvious on their face. For example, SPC72 and MID2 are both involved in meiotic processes but localized to different parts of the cell (the centriole and theplasma membrane, respectively). However, it has been shown that microtubule organization and membrane integrity sensing pathways do show interaction during gamete maturation [57]. Others, like the Ino80 complex related Les2 subunit and SPO71 appear to be directly involved in the biological process of DNA remodelling during recombination and it may be easier to imagine their mode of interaction and design experiments to probe it.(Gordon et al. 2006).

## Discussion

Formatted: Font: Bold

Formatted: Normal, Line spacing: single

We introduced a scalable system for phylogenetic profiling from hierarchical orthologous groups. The ROC and AUC values shown in the using an empirical benchmark ofin the first section 3.1of Results indicates that the minhashMinHash Jaccard score estimate between profiles is a

~~competitive alternative to~~has slightly better performance than previous tree and vector based metrics, while also being much faster to compute. This is remarkable in that one typically ~~expect~~expects a trade-off between speed and accuracy, which does not appear to be the case here. We hypothesise that the error introduced by the ~~fast minhash~~MinHash approximation is ~~more than~~ compensated by the inclusion of an unprecedented amount of genomes and taxonomic nodes in the labelled phylogenies used to construct the profiles.

Furthermore, while our ~~minhash~~MinHash-derived Jaccard estimates are able to capture some of the differences between interacting and non-interacting HOGs, as shown above, their unique strength lies in the fast recovery of ~~close~~the top k closest profiles within an LSH Forest. Once these profiles are recovered, the inference of submodules or network structure can be refined using other, potentially more compute intensive methods, on this much smaller subset of data.

~~Because phylogenetic profiling is not yet broadly used on eukaryotic data, HogProf is largely orthogonal to and thus particularly effective combined with existing functional annotations. We showed that HogProf was~~We have shown that HogProf is able to reconstitute the modular organisation of the kinetochore, as well as increase the list of protein families interacting within the network with several known interactors of the kinetochore and the APC. As for the other HOGs returned in these searches, our results suggest that some are yet unknown interactors involved in aspects of the cell cycle or ciliary dynamics. Likewise, our attempt at retrieving candidate members of the sexual reproduction network recapitulated many known interactions, while also suggesting new ones.

**Formatted:** Footer

The current paradigm for exploring interaction or participation in different biological pathways across protein families relies heavily on data integration strategies that take into account heterogenous high-throughput experiments and knowledge found in the literature. Many times, these datasets only describe the networks in question in one organism at a time. Furthermore, signaling, metabolic and physical interaction networks are all covered by different types of experiments and data produced by these systems is located in heterogeneous databases. By contrast, phylogenetic profiles can potentially uncover all three types of networks from sequencing data alone. This was highlighted in our work during retrieval of potential interactors within the sexual reproduction and kinetochore networks with the retrieval of LFR and CFAP157, respectively. CFAP157, a cilia and flagella associated protein might be involved in recruitment/regulation of APC-Cdc20 or ciliary kinases (e.g Nek1), both known to mediate APC regulation of ciliary dynamics (Wang and Kirschner, 2014). In both cases, a regulatory action within the network was the biological process which involved both the query and retrieved HOGs, not a physical interaction. The advances put forward by our new methodology and the property of retrieving entire networks and not just physical interactions opens the possibility of performing comparative profiling on an unprecedented scale and lays the groundwork for integrative modeling of the interplay between PPI, regulation and metabolic networks in a more holistic way.

Further work remains to be done on tuning the profile construction with the appropriate weights at each taxonomic level, as well as ~~when to construct~~constructing profiles for subfamiles arising from duplications which may undergo neofunctionalization~~.~~, a theme which has been previously explored in phylogenetic profiling efforts relying on far fewer genomes (Dey et al. 2015). Downstream processing of the explicit representation of the data, as opposed to the the hash

signature, can also be designed using more computationally intensive methods to detect interactions on smaller subsets of profiles after using the LSH as a first search.

The phylogenetic profiling pipeline presented in this work will be integrated into OMA web-based services. Meanwhile, it is already available on Github as a standalone package~~.~~ ( https://github.com/DessimozLab/HogProf).

## Methods

The following section details the creation of phylogenetic profiles using OMA data, their transformation into ~~minhash~~MinHash based probabilistic data structures and the ~~technical details of~~tools and libraries used in the implementation.

### Profile construction

To generate large-scale gene phylogenies labelled with speciation, duplication and loss events (a.k.a. *enhanced phylogenies* or *tree profiles*) for each HOG in OMA, we processed input data in OrthoXML format ~~[58]~~(Schmitt et al. 2011) with pyHam ~~[59],~~(Train et al. 2018), using the NCBI taxonomic tree ~~[60]~~(Sayers et al. 2010) pruned to contain only the genomes represented in OMA ~~[15].~~(Altenhoff et al. 2018). Tree profiles contain a species tree annotated at each taxonomic level with information on when the last common ancestor gene appeared, where losses and duplications occurred and the copy number of the gene at each taxonomic level. More information on the pyHam inference of evolutionary events can be found in ~~[59].~~(Train et al. 2018). pyHam can also be used to infer enhanced phylogenies for other datasets available in OrthoXML format such as ENSEMBL (Zerbino et al. 2018) or with data generated from phylogenetic trees such as those

**Formatted:** Footer

found in PANTHER (Mi et al. 2017) through the use of the function `etree2orthoxml()` in the tree analysis package ETE3 (Huerta-Cepas, Serra, and Bork 2016).

~~Using this gene tree~~The enhanced phylogeny trees for each HOG are parsed to create a vector representation of the ~~HOG, a multiset for~~ presence~~, loss~~ or absence of a homologue at each extant and ancestral node as well as the duplication ~~at each taxonomic level is compiled into a vector representation. In this representation each column corresponds to an evolutionary event or presence of a gene at a specific taxonomic level~~or loss events on the branch leading to that node. Each profile vector contains 9345 columns ( corresponding to the 3115 nodes of the taxonomy used and the ~~weight in the column corresponds to the weight (or importance) given to each node of the taxonomic tree for that class of events (~~3 categories of presence, loss and duplication ).

To encode profile vectors as weighted MinHash signatures (Sergey Ioffe 2010) we used the Datasketch library ("Datasketch: Big Data Looks Small — Datasketch 1.0.0 Documentation" n.d.).~~**Fig.** 1)~~. In this formulation, the Jaccard score between multisets ~~[61]~~ representing profiles ~~will~~can be more heavily influenced by nodes with a higher weight. ~~In this manuscript only profiles with binary vectors are considered; the optimization of weighting and other refinements of the profiling pipeline will be the subject of future publications.~~The final MinHash signatures used were built with 256 hashing functions.

Formatted: Font: Bold

~~**Profile construction with Weighted Minhashing and Database construction using LSH Forest**~~

Historically, distance metrics between profiles have fallen into two categories: tree based and vector based metrics [6,17]. Comparing all vs all profiles to define a distance matrix using metrics detailed in other phylogenetic profiling approaches, such as mutual information, Hamming distance or tree aware methods [6,18,62–64], scales quadratically with the number of profiles. The time it takes to calculate profiles and a distance between two profiles typically scales poorly with the number of genomes considered, especially with tree based methods. These computations are not practical when comparing the labelled phylogenies produced by pyHam for all HOGs in OMA, even with high performance computing.

Several studies have established the Jaccard similarity [65] between two profiles of presence and absence patterns across extant genomes as a valid phylogenetic profiling distance metric, which is able to capture an evolutionary signal closely related to shared protein functions [18,66,67]. This profile distance metric integrates well with the available algorithms and data structures available in the Datasketch library [68]. These data structures are built around minhashing techniques to retrieve similar sets of elements in sublinear time and allow a user to efficiently search the profile space without explicitly calculating the distance matrix between all profiles, as well as approximate the Jaccard similarity between profiles, by comparing hash signatures. Using these data structures to represent and search for the phylogenetic profiles effectively removes the necessity for an all-vs-all comparison.

Minhashing techniques were devised to measure the similarity of documents and search for similar documents within large datasets containing billions of elements [69–71]. A document can either be encoded as a set of unique words that occur within it or as a multiset representing the number of occurrences of each unique word. When dealing with sets where the total number of unique

**Formatted:** Footer

elements is unknown before processing all sets, it is preferable to encode them using the minhash algorithm, which allows the hash signature of the set to be updated as new unique elements are added without prior knowledge of all possible elements. When the total set of unique elements (e.g. all of the possible words in a corpus of documents or all of the taxa present in the species tree) is known, it is possible to use a minhash signature to represent the number of times each type of element occurs in a multiset of all possible elements. This representation is known as a weighted minhash and, depending on the dataset, may be more precise in retrieving relevant hash signatures (e.g. a document that mentions a specific word many times). The mathematical principles underpinning weighted minhashing and locality sensitive hashing forest algorithms and their implementation are described in earlier papers [61,72,73].

After transforming HOG profile vectors to their corresponding weighted minhashesMinHashes using the datasketch library, an estimation of the Jaccard distance between profiles can be obtained by calculating the Hamming distance between their hash signatures [61].(S. Ioffe 2010). The speed of comparison and lower bound for accuracy of the estimation of the Jaccard score is set by the number of hashing functions. The comparison of hash signatures has $O(N)$ time complexity where N is the number of hash functions used to generate the minhashMinHash signature. Due to this property, an arbitrary number of elements can be encoded in this signature without slowing down comparisons. In our use case, this enables the use of an arbitrarily large number of taxa for which we can consider evolutionary events. With other metrics, such as Pearson correlation between vectors, the profile comparison between vectors scales linearly with the number or genomes or taxa considered in the best case scenario. In more complicated tree based methods, these comparisons can be much more costlyAdditionally, hardware implementations of

hash functions allow the calculation of hash signatures at rates of giga hashes per second and allow for extremely fast implementation of this step, placing the bottleneck of the pipeline at the calculation of enhanced phylogenies.

~~Weighted minhash~~The weighted MinHash objects ~~can also be used to compile~~for each HOG's enhanced phylogeny were compiled into a searchable data structure referred to as a Locality Sensitive Hashing Forest (LSH Forest) ~~[72].~~(Bawa, Condie, and Ganesan 2005) and their signatures were stored in an HDF5 file. The LSH Forest can be queried with a hash signature to retrieve the K neighbors with the highest Jaccard similarity to the query hash. The K closest hashes are retrieved from a B-Tree data structure ~~[74].~~(Comer 1979). This branching tree data structure allows for the querying and dynamic insertion~~,~~ and deletion ~~and querying~~ of elements in the LSH Forest data structure built upon it ~~at orders of magnitude faster than previous profiling efforts. As previously mentioned, calculating linkages between all groups in non probabilistic data structures requires an all vs all comparison of profiles which scales quadratically~~ with ~~the number of profiles in the dataset and can easily become computationally prohibitive. This penalty also applies whenever new genomes or taxonomic levels are added to the input matrix and the linkages must be recalculated. In the case of the LSH Forest, hash signatures of HOGs containing the new genome can be deleted and replaced in the database with a~~ logarithmic time complexity.

The scaling properties of the MinHash data structures when compared to pairwise distance calculations and hierarchical clustering are shown below in Figure 6.

**Formatted:** Footer

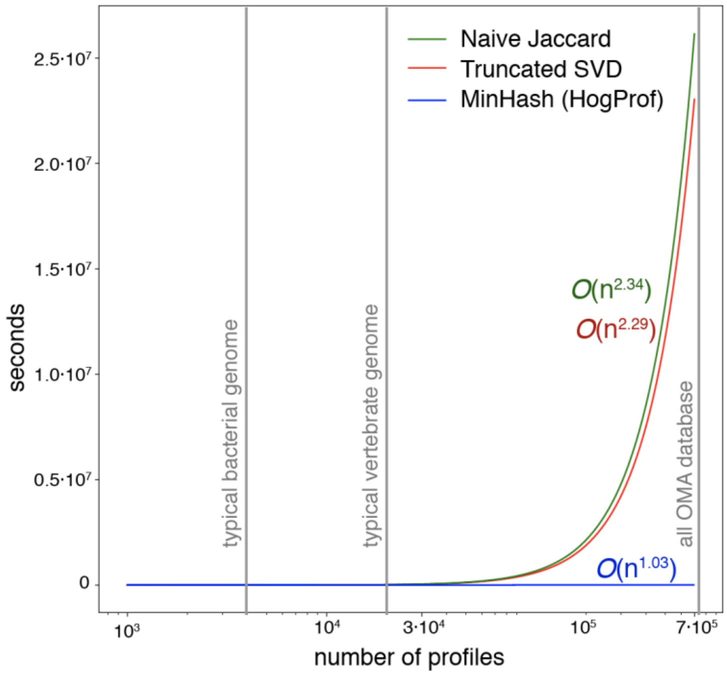


**Fig.** that scales logarithmically with the number of HOGs in the dataset. In non-probabilistic**6.**

Formatted: Font: Bold

Formatted: Indent: Left: 0.25"

To illustrate the advantageous scaling properties of MinHash data structures, whenever a new HOG is added to an existing input matrix and linkages are recalculated, the penalty is linearly proportional to the number of HOGs already in the datasetsynthetic profiles of length 100 were generated in the form of binary vectors (0 and the number of HOGs added whereas in the case of the LSH Forest, the time complexity scales logarithmically with the number of HOGs already in the dataset1 equiprobable). Profiles were then clustered using an explicit calculation of the Jaccard distance, reduced to a lower dimensionality (5 dimensions) with truncated SVD, normalized and linearly with the number of HOGs added. Query time complexity inexplicitly clustered using Euclidean distance as in SVD-Phy (Franceschini et al.

2016) or transformed into MinHash signatures and inserted into an LSH Forest object as in our method. Orders of magnitude showing typical profiling approaches is heavily penalized for the number of orthologous groups and genomes included in the analysis whereas HogProf is unaffected by the number of genomes included (since it is only dependentuse cases for profiling pipelines are shown on the number of hash functions used to generate the weighted minhash signature of HOGs) and scales logarithmically with the number of HOGs added to the database.x-axis. Curves were fitted to each set of timepoints to empirically determine the time complexity of each approach.

Orthology

## Computational resources, data and software libraries used

Our dataset contains approximately 600,000 HOGs computed from the 2,167 genomes in OMA (June 2018 release) The main computational bottleneck in our pipeline is the calculation of the labelled gene trees for each HOG using pyHam. Even with this computation, compiled LSH forest objects containing the hash signatures of all HOGs' gene trees can be compiled in under 3 hours (with 10 CPUs but this can scale easily to more cores) with only 2.5 GB of RAM and queried extremely efficiently (an average of 0.01 seconds over 1000 queries against a database containing profiles for all HOGs in OMA on an Intel(R) Xeon(R) CPU E5530 @ 2.40 GHz and 2 GB of RAM to load the LSH database object into memory). This performance makes it possible to provide online search functionality, which we aim to release in an upcoming web-based version of the OMA browser. Meanwhile, the compiled profile database can be used for analysis on typical workstations (note that memory and CPU requirements will depend on the number of hash functions

**Formatted:** Footer

implemented in the construction of profiles and the filtering of the initial dataset to clades of interest to the user).

All gene ontology (GO) annotations (encompassing molecular functions, cellular locations, and biological processes) for HOGs contained in OMA were analyzed with GOATOOLS [75].(Klopfenstein et al. 2018). To calculate the enrichment of annotations, the results returned by the LSH Forest annotations for all protein sequences contained in the HOGs returned by the search were collected and the entire OMA annotation corpus was used as background.

HDF5 files were compiled with H5PY (ver. 2.9.0). Pandas (ver. 0.24.0) was used for data manipulation. Labelled phylogenies were manipulated with ete3ETE3 [76].(Huerta-Cepas, Serra, and Bork 2016). Datasketch (ver. 1.0.0) was used to compile weighted minhashesMinHashes and LSH Forest data structures. Plots were generated using matplotlib (ver. 3.0.2). PyHam (ver 1.1.6) was used to calculate labelled phylogenies for the HOGs in OMA.

Time complexity analysis in Figure 6 was done with the scikit-learn implementation of truncated SVD (Pedregosa et al. 2011) and scipy (Jones, Oliphant, and Peterson 2001) distance functions.

**Pearson and Spearman correlation comparison of distance matrices**

Distance matrices between all pairs of profiles in the kinetochore and APC complex protein families defined in [9](van Hooff et al. 2017) were compared using the Spearman and Pearson statistical analysis functions from the the SciPy python package to verify the monotonicity of the scores between families.

## Acknowledgements

We thank Monique Zahn for helpful feedback on the manuscript. This work was funded by a grant by the Novartis Foundation for Medical-Biological Research (#17B111 to CD), by the Swiss National Science Foundation (Grant 183723 to CD), by the Swiss Leading House for the Latin American Region (to CD and PSA), and by the Agencia Nacional de Promoción Científica y Tecnológica (PICT-2017-0854 to PSA) .

*Conflict of Interest:* none declared.

## Supplementary data

- **Supplementary Data 1—kineto_augment_goenrich.csv**: Contains the results of GO enrichment analysis done on the results of our search for kinetochore interactors. After searching with the HOGs corresponding to each of the kinetochore components, the returned HOGs were clustered according to their jaccard similarity. Using a hierarchical clustering and a manually defined cutoff the results were separated into discrete clusters. Each cluster was analyzed using goatools for GO enrichment. Enrichment results for clusters containing a query gene were recorded in this CSV file.
- **Supplementary Data 2—hap_enrich.csv**: Contains the goatools output for the GO enrichment analysis of the top 100 closest coevolving HOGs returned by a query with Hap2.
- **Supplementary Data 3—gex_enrich.csv**: Contains the goatools output for the GO enrichment analysis of the top 100 closest coevolving HOGs returned by a query with Gex1.
- **Supplementary Data 5—repro_augment_goenrich.csv:** Contains the results of GO enrichment analysis done on the results of our search for sexual reproduction network interactors. After searching with the HOGs corresponding to each of the manually curated list of conserved sexual reporduction network components, the returned HOGs were clustered according to their jaccard similarity. Using a hierarchical clustering and a manually defined cutoff the results were separated into discrete clusters. Each cluster was analyzed using

**Formatted:** Normal (Web), Indent: Left: 0.25", Hanging: 0.25", Line spacing: 1.5 lines, Outline numbered + Level: 1 + Numbering Style: Bullet + Aligned at: 0.25" + Tab after: 0.5" + Indent at: 0.5", Font Alignment: Baseline

**Formatted:** Footer

goatools for GO enrichment. Enrichment results for clusters containing a query were recorded in this csv file.

Formatted: Font: (Default) Arial, 11 pt, Font color: Black

Formatted: Indent: Left: 0"

# References


1. Skunca N, Altenhoff A, Dessimoz C. Quality of computationally inferred gene ontology annotations. PLoS Comput Biol. 2012;8: e1002533.
2. Cozzetto D, Jones DT. Computational Methods for Annotation Transfers from Sequence. Methods Mol Biol. 2017;1446: 55–67.
3. Pellegrini M, Marcotte EM, Thompson MJ, Eisenberg D, Yeates TO. Assigning protein functions by comparative genome analysis: protein phylogenetic profiles. Proc Natl Acad Sci U S A. 1999;96: 4285–4288.
4. Snitkin ES, Gustafson AM, Mellor J, Wu J, DeLisi C. 10.1186/1471-2105-7-420. BMC Bioinformatics. 2006. p. 420. doi:10.1186/1471-2105-7-420
5. Jothi R, Przytycka TM, Aravind L. Discovering functional linkages and uncharacterized cellular pathways using phylogenetic profile comparisons: a comprehensive assessment. BMC Bioinformatics. 2007;8: 173.
6. Ruano-Rubio V, Poch O, Thompson JD. Comparison of eukaryotic phylogenetic profiling approaches using species tree aware methods. BMC Bioinformatics. 2009;10: 383.
7. Mukherjee S, Stamatis D, Bertsch J, Ovchinnikova G, Verezemska O, Isbandi M, et al. Genomes OnLine Database (GOLD) v.6: data updates and feature enhancements. Nucleic Acids Res. 2017;45: D446–D456.
8. Tabach Y, Billi AC, Hayes GD, Newman MA, Zuk O, Gabel H, et al. Identification of small RNA pathway genes using patterns of phylogenetic conservation and divergence. Nature. 2013;493: 694–698.
9. van Hooff JJ, Tromer E, van Wijk LM, Snel B, Kops GJ. Evolutionary dynamics of the kinetochore network in eukaryotes as revealed by comparative genomics. EMBO Rep. 2017;18: 1559–1571.
10. Nevers Y, Prasad MK, Poidevin L, Chennen K, Allot A, Kress A, et al. Insights into Ciliary Genes and Evolution from Multi-Level Phylogenetic Profiling. Mol Biol Evol. 2017;34: 2016–2034.
11. Sherill-Rofe D, Rahat D, Findlay S, Mellul A, Guberman I, Braun M, et al. Mapping global and local coevolution across 600 species to identify novel homologous recombination repair genes. Genome

Research. 2019. pp. 439–448. doi:10.1101/gr.241414.118

12. Zerbino DR, Achuthan P, Akanni W, Amode MR, Barrell D, Bhai J, et al. Ensembl 2018. Nucleic Acids Res. 2018;46: D754–D761.

13. Huerta-Cepas J, Szklarczyk D, Forslund K, Cook H, Heller D, Walter MC, et al. eggNOG 4.5: a hierarchical orthology framework with improved functional annotations for eukaryotic, prokaryotic and viral sequences. Nucleic Acids Res. 2016;44: D286–93.

14. Zdobnov EM, Tegenfeldt F, Kuznetsov D, Waterhouse RM, Simão FA, Ioannidis P, et al. OrthoDB v9.1: cataloging evolutionary and functional annotations for animal, fungal, plant, archaeal, bacterial and viral orthologs. Nucleic Acids Res. 2017;45: D744–D749.

15. Altenhoff AM, Glover NM, Train C-M, Kaleb K, Warwick Vesztrocy A, Dylus D, et al. The OMA orthology database in 2018: retrieving evolutionary relationships among all domains of life through richer web and programmatic interfaces. Nucleic Acids Res. 2018;46: D477–D485.

16. Ta HX, Koskinen P, Holm L. A novel method for assigning functional linkages to proteins using enhanced phylogenetic trees. Bioinformatics. 2011;27: 700–706.

17. Kensche PR, van Noort V, Dutilh BE, Huynen MA. Practical and theoretical advances in predicting the function of a protein by its phylogenetic distribution. J R Soc Interface. 2008;5: 151–170.

18. Niu Y, Liu C, Moghimyfiroozabad S, Yang Y, Alavian KN. PrePhyloPro: phylogenetic profile-based prediction of whole proteome linkages. PeerJ. 2017;5: e3712.

19. Li Y, Calvo SE, Gutman R, Liu JS, Mootha VK. Expansion of biological pathways based on evolutionary inference. Cell. 2014;158: 213–225.

20. Altenhoff AM, Gil M, Gonnet GH, Dessimoz C. Inferring hierarchical orthologous groups from orthologous gene pairs. PLoS One. 2013;8: e53786.

21. Giurgiu M, Reinhard J, Brauner B, Dunger-Kaltenbach I, Fobo G, Frishman G, et al. CORUM: the comprehensive resource of mammalian protein complexes-2019. Nucleic Acids Res. 2018. doi:10.1093/nar/gky973

22. Mewes HW, Amid C, Arnold R, Frishman D, Güldener U, Mannhaupt G, et al. MIPS: analysis and annotation of proteins from whole genomes. Nucleic Acids Res. 2004;32: D41–4.

23. Glazko GV, Mushegian AR. Detection of evolutionarily stable fragments of cellular pathways by hierarchical clustering of phyletic patterns. Genome Biol. 2004;5: R32.

**Formatted:** Footer

24. Ranea JAG, Yeats C, Grant A, Orengo CA. Predicting protein function with hierarchical phylogenetic profiles: the Gene3D Phylo-Tuner method applied to eukaryotic genomes. PLoS Comput Biol. 2007;3: e237.

25. Ashburner M, Ball CA, Blake JA, Botstein D, Butler H, Cherry JM, et al. Gene ontology: tool for the unification of biology. The Gene Ontology Consortium. Nat Genet. 2000;25: 25–29.

26. Dessimoz C, Škunca N, Thomas PD. CAFA and the open world of protein function predictions. Trends Genet. 2013;29: 609–610.

27. Weidemann M, Schuster-Gossler K, Stauber M, Wrede C, Hegermann J, Ott T, et al. CFAP157 is a murine downstream effector of FOXJ1 that is specifically required for flagellum morphogenesis and sperm motility. Development. 2016;143: 4736–4748.

28. Pattabiraman S, Baumann C, Guisado D, Eppig JJ, Schimenti JC, De La Fuente R. Mouse BRWD1 is critical for spermatid postmeiotic transcription and female meiotic chromosome stability. J Cell Biol. 2015;208: 53–69.

29. Wang J, Dye BT, Rajashankar KR, Kurinov I, Schulman BA. Insights into anaphase promoting complex TPR subdomain assembly from a CDC26-APC6 structure. Nat Struct Mol Biol. 2009;16: 987–989.

30. Cheeseman LP, Harry EF, McAinsh AD, Prior IA, Royle SJ. Specific removal of TACC3-ch-TOG-clathrin at metaphase deregulates kinetochore fiber tension. J Cell Sci. 2013;126: 2102–2113.

31. Wang W, Wu T, Kirschner MW. The master cell cycle regulator APC-Cdc20 regulates ciliary length and disassembly of the primary cilium. Elife. 2014;3: e03083.

32. Speijer D, Lukeš J, Eliáš M. Sex is a ubiquitous, ancient, and inherent attribute of eukaryotic life. Proc Natl Acad Sci U S A. 2015;112: 8827–8834.

33. Altenhoff AM, Studer RA, Robinson-Rechavi M, Dessimoz C. Resolving the Ortholog Conjecture: Orthologs Tend to Be Weakly, but Significantly, More Similar in Function than Paralogs. PLoS Comput Biol. 2012;8: e1002514.

34. Liu Y, Tewari R, Ning J, Blagborough AM, Garbom S, Pei J, et al. The conserved plant sterility gene HAP2 functions after attachment of fusogenic membranes in Chlamydomonas and Plasmodium gametes. Genes Dev. 2008;22: 1051–1068.

35. Valansi C, Moi D, Leikina E, Matveev E, Graña M, Chernomordik LV, et al. Arabidopsis HAP2/GCS1 is a gamete fusion protein homologous to somatic and viral fusogens. The Journal of cell biology. 2017. pp. 571–581.

36. Fédry J, Liu Y, Péhau-Arnaudet G, Pei J, Li W, Tortorici MA, et al. The Ancient Gamete Fusogen HAP2 Is a Eukaryotic Class II Fusion Protein. Cell. 2017;168: 904–915.e10.

37. Feng J, Dong X, Springer TA. Fusion surface structure, function, and dynamics of gamete fusogen HAP2. 2018. doi:10.2210/pdb6dbs/pdb

38. Hurtado L, Farrona S, Reyes JC. The putative SWI/SNF complex subunit BRAHMA activates flower homeotic genes in Arabidopsis thaliana. Plant Mol Biol. 2006;62: 291–304.

39. Dawe AL, Caldwell KA, Harris PM, Morris NR, Caldwell GA. Evolutionarily conserved nuclear migration genes required for early embryonic development in Caenorhabditis elegans. Dev Genes Evol. 2001;211: 434–441.

40. Wang X-T, Yuan C, Yuan T-T, Cui S-J. The Arabidopsis LFR gene is required for the formation of anther cell layers and normal expression of key regulatory genes. Mol Plant. 2012;5: 993–1000.

41. Ning J, Otto TD, Pfander C, Schwach F, Brochet M, Bushell E, et al. Comparative genomics in Chlamydomonas and Plasmodium identifies an ancient nuclear envelope protein family essential for sexual reproduction in protists, fungi, plants, and vertebrates. Genes Dev. 2013;27: 1198–1215.

42. Alandete-Saez M, Ron M, Leiboff S, McCormick S. Arabidopsis thaliana GEX1 has dual functions in gametophyte development and early embryogenesis: Dual functions of GEX1. Plant J. 2011;68: 620–632.

43. Wijeratne AJ, Chen C, Zhang W, Timofejeva L, Ma H. The Arabidopsis thaliana PARTING DANCERS gene encoding a novel protein is required for normal meiotic homologous recombination. Mol Biol Cell. 2006;17: 1331–1343.

44. Keeney S, Giroux CN, Kleckner N. Meiosis-specific DNA double-strand breaks are catalyzed by Spo11, a member of a widely conserved protein family. Cell. 1997;88: 375–384.

45. Strzalka W, Ziemienowicz A. Proliferating cell nuclear antigen (PCNA): a key factor in DNA replication and cell cycle regulation. Ann Bot. 2011;107: 1127–1140.

46. Malik S-B, Pightling AW, Stefaniak LM, Schurko AM, Logsdon JM Jr. An expanded inventory of conserved meiotic genes provides evidence for sex in Trichomonas vaginalis. PLoS One. 2007;3: e2879.

47. Loidl J. Conservation and Variability of Meiosis Across the Eukaryotes. Annu Rev Genet. 2016;50: 293–316.

48. Schurko AM, Logsdon JM Jr. Using a meiosis detection toolkit to investigate ancient asexual

**Formatted:** Footer

"scandals" and the evolution of sex. Bioessays. 2008;30: 579–589.

49. Niklas KJ, Cobb ED, Kutschera U. Did meiosis evolve before sex and the evolution of eukaryotic life cycles? Bioessays. 2014;36: 1091–1101.

50. Goodenough U, Heitman J. Origins of eukaryotic sexual reproduction. Cold Spring Harb Perspect Biol. 2014;6. doi:10.1101/cshperspect.a016154

51. Zelkowski M, Zelkowska K, Conrad U, Hesse S, Lermontova I, Marzec M, et al. Arabidopsis NSE4 Proteins Act in Somatic Nuclei and Meiosis to Ensure Plant Viability and Fertility. Front Plant Sci. 2019;10: 774.

52. Rajavel M, Philip B, Buehrer BM, Errede B, Levin DE. Mid2 is a putative sensor for cell integrity signaling in Saccharomyces cerevisiae. Mol Cell Biol. 1999;19: 3969–3976.

53. Serber DW, Runge JS, Menon DU, Magnuson T. The Mouse INO80 Chromatin-Remodeling Complex Is an Essential Meiotic Factor for Spermatogenesis. Biol Reprod. 2016;94: 8.

54. Bao Y, Shen X. SnapShot: Chromatin remodeling: INO80 and SWR1. Cell. 2011;144: 158–158.e2.

55. Leza MA, Elion EA. POG1, a novel yeast gene, promotes recovery from pheromone arrest via the G1 cyclin CLN2. Genetics. 1999;151: 531–543.

56. van Werven FJ, Neuert G, Hendrick N, Lardenois A, Buratowski S, van Oudenaarden A, et al. Transcription of two long noncoding RNAs mediates mating-type control of gametogenesis in budding yeast. Cell. 2012;150: 1170–1181.

57. Gordon O, Taxis C, Keller PJ, Benjak A, Stelzer EHK, Simchen G, et al. Nud1p, the yeast homolog of Centriolin, regulates spindle pole body inheritance in meiosis. EMBO J. 2006;25: 3856–3868.

58. Schmitt T, Messina DN, Schreiber F, Sonnhammer ELL. Letter to the editor: SeqXML and OrthoXML: standards for sequence and orthology information. Brief Bioinform. 2011;12: 485–488.

59. Train C-M, Pignatelli M, Altenhoff A, Dessimoz C. iHam & pyHam: visualizing and processing hierarchical orthologous groups. Bioinformatics. 2018. doi:10.1093/bioinformatics/bty994

60. Sayers EW, Barrett T, Benson D a., Bolton E, Bryant SH, Canese K, et al. Database resources of the National Center for Biotechnology Information. Nucleic Acids Res. 2010;38: D5–16.

61. Ioffe S. Improved Consistent Sampling, Weighted Minhash and L1 Sketching. 2010 IEEE International Conference on Data Mining. 2010. pp. 246–255.

62. Tillier ERM, Charlebois RL. The human protein coevolution network. Genome Res. 2009;19: 1861–1871.

63. Wu J, Hu Z, DeLisi C. Gene annotation and network inference by phylogenetic profiling. BMC Bioinformatics. 2006;7: 80.

64. Dey G, Jaimovich A, Collins SR, Seki A, Meyer T. Systematic Discovery of Human Gene Function and Principles of Modular Organization through Phylogenetic Profiling. Cell Rep. 2015. doi:10.1016/j.celrep.2015.01.025

65. Jaccard P. Étude comparative de la distribution florale dans une portion des Alpes et des Jura. Bull Soc Vaud sci nat. 1901;37: 547–579.

66. Psomopoulos FE, Mitkas PA, Ouzounis CA. Detection of genomic idiosyncrasies using fuzzy phylogenetic profiles. PLoS One. 2013;8: e52854.

67. Brilli M, Mengoni A, Fondi M, Bazzicalupo M, Liò P, Fani R. Analysis of plasmid genes by phylogenetic profiling and visualization of homology relationships using Blast2Network. BMC Bioinformatics. 2008;9: 551.

68. datasketch: Big Data Looks Small — datasketch 1.0.0 documentation. [cited 26 Sep 2018]. Available: https://ekzhu.github.io/datasketch/index.html

69. Manber U. Finding similar files in a large file system. Usenix Winter. 1994. pp. 1–10.

70. Broder AZ. On the resemblance and containment of documents. Compression and Complexity of Sequences 1997 Proceedings. IEEE; 1997. pp. 21–29.

71. Broder AZ, Charikar M, Frieze AM, Mitzenmacher M. Min-Wise Independent Permutations. J Comput System Sci. 2000;60: 630–659.

72. Bawa M, Condie T, Ganesan P. LSH Forest: Self-tuning Indexes for Similarity Search. Proceedings of the 14th International Conference on World Wide Web. New York, NY, USA: ACM; 2005. pp. 651–660.

73. Andoni A, Indyk P. Near-Optimal Hashing Algorithms for Approximate Nearest Neighbor in High Dimensions. 2006 47th Annual IEEE Symposium on Foundations of Computer Science (FOCS'06). 2006. pp. 459–468.

74. Comer D. Ubiquitous B-Tree. ACM Computing Surveys (CSUR). 1979;11: 121–137.

75. Klopfenstein DV, Zhang L, Pedersen BS, Ramírez F, Warwick Vesztrocy A, Naldi A, et al. GOATOOLS: A Python library for Gene Ontology analyses. Sci Rep. 2018;8: 10872.

76. Huerta-Cepas J, Serra F, Bork P. ETE 3: Reconstruction, Analysis, and Visualization of Phylogenomic Data. Mol Biol Evol. 2016;33: 1635–1638.

**Formatted:** Footer

Alandete-Saez, Monica, Mily Ron, Samuel Leiboff, and Sheila McCormick. 2011. "Arabidopsis Thaliana GEX1 Has Dual Functions in Gametophyte Development and Early Embryogenesis: Dual Functions of GEX1." *The Plant Journal: For Cell and Molecular Biology* 68 (4): 620–32.

Altenhoff, Adrian M., Manuel Gil, Gaston H. Gonnet, and Christophe Dessimoz. 2013. "Inferring Hierarchical Orthologous Groups from Orthologous Gene Pairs." *PloS One* 8 (1): e53786.

Altenhoff, Adrian M., Natasha M. Glover, Clément-Marie Train, Klara Kaleb, Alex Warwick Vesztrocy, David Dylus, Tarcisio M. de Farias, et al. 2018. "The OMA Orthology Database in 2018: Retrieving Evolutionary Relationships among All Domains of Life through Richer Web and Programmatic Interfaces." *Nucleic Acids Research* 46 (D1): D477–85.

Altenhoff, Adrian M., Romain A. Studer, Marc Robinson-Rechavi, and Christophe Dessimoz. 2012. "Resolving the Ortholog Conjecture: Orthologs Tend to Be Weakly, but Significantly, More Similar in Function than Paralogs." *PLoS Computational Biology* 8 (5): e1002514.

Ashburner, M., C. A. Ball, J. A. Blake, D. Botstein, H. Butler, J. M. Cherry, A. P. Davis, et al. 2000. "Gene Ontology: Tool for the Unification of Biology. The Gene Ontology Consortium." *Nature Genetics* 25 (1): 25–29.

Bao, Yunhe, and Xuetong Shen. 2011. "SnapShot: Chromatin Remodeling: INO80 and SWR1." *Cell* 144 (1): 158–158.e2.

Bawa, Mayank, Tyson Condie, and Prasanna Ganesan. 2005. "LSH Forest: Self-Tuning Indexes for Similarity Search." In *Proceedings of the 14th International Conference on World Wide Web*, 651–60. WWW '05. New York, NY, USA: ACM.

Cheeseman, Liam P., Edward F. Harry, Andrew D. McAinsh, Ian A. Prior, and Stephen J. Royle. 2013. "Specific Removal of TACC3-Ch-TOG-Clathrin at Metaphase Deregulates Kinetochore Fiber Tension." *Journal of Cell Science* 126 (Pt 9): 2102–13.

Comer, Douglas. 1979. "Ubiquitous B-Tree." *ACM Computing Surveys (CSUR)* 11 (2): 121–37.

Cozzetto, Domenico, and David T. Jones. 2017. "Computational Methods for Annotation Transfers from Sequence." *Methods in Molecular Biology* 1446: 55–67.

"Datasketch: Big Data Looks Small — Datasketch 1.0.0 Documentation." n.d. Accessed September 26, 2018. https://ekzhu.github.io/datasketch/index.html.

Dessimoz, Christophe, Nives Škunca, and Paul D. Thomas. 2013. "CAFA and the Open World of Protein Function Predictions." *Trends in Genetics: TIG* 29 (11): 609–10.

Dey, Gautam, Ariel Jaimovich, Sean R. Collins, Akiko Seki, and Tobias Meyer. 2015. "Systematic Discovery of Human Gene Function and Principles of Modular Organization through Phylogenetic Profiling." *Cell Reports*, February. https://doi.org/10.1016/j.celrep.2015.01.025.

Fédry, Juliette, Yanjie Liu, Gérard Péhau-Arnaudet, Jimin Pei, Wenhao Li, M. Alejandra Tortorici, François Traincard, et al. 2017. "The Ancient Gamete Fusogen HAP2 Is a Eukaryotic Class II Fusion Protein." *Cell* 168 (5): 904–15.e10.

Feng, J., X. Dong, and T. A. Springer. 2018. "Fusion Surface Structure, Function, and Dynamics of Gamete

Formatted: French (France)
Field Code Changed
Formatted: French (France)
Field Code Changed
Formatted: French (France)
Formatted: French (France)
Field Code Changed
Formatted: French (France)
Field Code Changed
Formatted: French (France)

Fusogen HAP2." https://doi.org/10.2210/pdb6dbs/pdb.

Franceschini, Andrea, Jianyi Lin, Christian von Mering, and Lars Juhl Jensen. 2016. "SVD-Phy: Improved Prediction of Protein Functional Associations through Singular Value Decomposition of Phylogenetic Profiles." *Bioinformatics* 32 (7): 1085–87.

Giurgiu, Madalina, Julian Reinhard, Barbara Brauner, Irmtraud Dunger-Kaltenbach, Gisela Fobo, Goar Frishman, Corinna Montrone, and Andreas Ruepp. 2018. "CORUM: The Comprehensive Resource of Mammalian Protein Complexes-2019." *Nucleic Acids Research*, October. https://doi.org/10.1093/nar/gky973.

Glazko, Galina V., and Arcady R. Mushegian. 2004. "Detection of Evolutionarily Stable Fragments of Cellular Pathways by Hierarchical Clustering of Phyletic Patterns." *Genome Biology* 5 (5): R32.

Goodenough, Ursula, and Joseph Heitman. 2014. "Origins of Eukaryotic Sexual Reproduction." *Cold Spring Harbor Perspectives in Biology* 6 (3). https://doi.org/10.1101/cshperspect.a016154.

Gordon, Oren, Christof Taxis, Philipp J. Keller, Aleksander Benjak, Ernst H. K. Stelzer, Giora Simchen, and Michael Knop. 2006. "Nud1p, the Yeast Homolog of Centriolin, Regulates Spindle Pole Body Inheritance in Meiosis." *The EMBO Journal* 25 (16): 3856–68.

Hooff, Jolien Je van, Eelco Tromer, Leny M. van Wijk, Berend Snel, and Geert Jpl Kops. 2017. "Evolutionary Dynamics of the Kinetochore Network in Eukaryotes as Revealed by Comparative Genomics." *EMBO Reports* 18 (9): 1559–71.

Huerta-Cepas, Jaime, François Serra, and Peer Bork. 2016. "ETE 3: Reconstruction, Analysis, and Visualization of Phylogenomic Data." *Molecular Biology and Evolution* 33 (6): 1635–38.

Huerta-Cepas, Jaime, Damian Szklarczyk, Kristoffer Forslund, Helen Cook, Davide Heller, Mathias C. Walter, Thomas Rattei, et al. 2016. "eggNOG 4.5: A Hierarchical Orthology Framework with Improved Functional Annotations for Eukaryotic, Prokaryotic and Viral Sequences." *Nucleic Acids Research* 44 (D1): D286–93.

Ioffe, S. 2010. "Improved Consistent Sampling, Weighted Minhash and L1 Sketching." In *2010 IEEE International Conference on Data Mining*, 246–55.

Ioffe, Sergey. 2010. "Improved Consistent Sampling, Weighted Minhash and textL1 Sketching ICDM." *Sydney, AU*.

Jones, Eric, Travis Oliphant, and Pearu Peterson. 2001. "SciPy: Open Source Scientific Tools for Python." https://www.scienceopen.com/document?vid=ab12905a-8a5b-43d8-a2bb-defc771410b9.

Jothi, Raja, Teresa M. Przytycka, and L. Aravind. 2007. "Discovering Functional Linkages and Uncharacterized Cellular Pathways Using Phylogenetic Profile Comparisons: A Comprehensive Assessment." *BMC Bioinformatics* 8 (May): 173.

Keeney, S., C. N. Giroux, and N. Kleckner. 1997. "Meiosis-Specific DNA Double-Strand Breaks Are Catalyzed by Spo11, a Member of a Widely Conserved Protein Family." *Cell* 88 (3): 375–84.

Kensche, Philip R., Vera van Noort, Bas E. Dutilh, and Martijn A. Huynen. 2008. "Practical and Theoretical Advances in Predicting the Function of a Protein by Its Phylogenetic Distribution." *Journal of the*

**Formatted:** German (Germany)

**Field Code Changed**

**Formatted:** Footer

*Royal Society, Interface / the Royal Society* 5 (19): 151–70.

Klopfenstein, D. V., Liangsheng Zhang, Brent S. Pedersen, Fidel Ramírez, Alex Warwick Vesztrocy, Aurélien Naldi, Christopher J. Mungall, et al. 2018. "GOATOOLS: A Python Library for Gene Ontology Analyses." *Scientific Reports* 8 (1): 10872.

Leza, M. A., and E. A. Elion. 1999. "POG1, a Novel Yeast Gene, Promotes Recovery from Pheromone Arrest via the G1 Cyclin CLN2." *Genetics* 151 (2): 531–43.

Liu, Yanjie, Rita Tewari, Jue Ning, Andrew M. Blagborough, Sara Garbom, Jimin Pei, Nick V. Grishin, et al. 2008. "The Conserved Plant Sterility Gene HAP2 Functions after Attachment of Fusogenic Membranes in Chlamydomonas and Plasmodium Gametes." *Genes & Development* 22 (8): 1051–68.

Li, Yang, Sarah E. Calvo, Roee Gutman, Jun S. Liu, and Vamsi K. Mootha. 2014. "Expansion of Biological Pathways Based on Evolutionary Inference." *Cell* 158 (1): 213–25.

Loidl, Josef. 2016. "Conservation and Variability of Meiosis Across the Eukaryotes." *Annual Review of Genetics* 50 (November): 293–316.

Lu, Yajuan, Xiaoxin Dai, Mianqun Zhang, Yilong Miao, Changyin Zhou, Zhaokang Cui, and Bo Xiong. 2017. "Cohesin Acetyltransferase Esco2 Regulates SAC and Kinetochore Functions via Maintaining H4K16 Acetylation during Mouse Oocyte Meiosis." *Nucleic Acids Research* 45 (16): 9388–97.

Malik, Shehre-Banoo, Arthur W. Pightling, Lauren M. Stefaniak, Andrew M. Schurko, and John M. Logsdon Jr. 2007. "An Expanded Inventory of Conserved Meiotic Genes Provides Evidence for Sex in Trichomonas Vaginalis." *PloS One* 3 (8): e2879.

Mewes, H. W., C. Amid, R. Arnold, D. Frishman, U. Güldener, G. Mannhaupt, M. Münsterkötter, et al. 2004. "MIPS: Analysis and Annotation of Proteins from Whole Genomes." *Nucleic Acids Research* 32 (Database issue): D41–44.

Mi, Huaiyu, Xiaosong Huang, Anushya Muruganujan, Haiming Tang, Caitlin Mills, Diane Kang, and Paul D. Thomas. 2017. "PANTHER Version 11: Expanded Annotation Data from Gene Ontology and Reactome Pathways, and Data Analysis Tool Enhancements." *Nucleic Acids Research* 45 (D1): D183–89.

Mukherjee, Supratim, Dimitri Stamatis, Jon Bertsch, Galina Ovchinnikova, Olena Verezemska, Michelle Isbandi, Alex D. Thomas, et al. 2017. "Genomes OnLine Database (GOLD) v.6: Data Updates and Feature Enhancements." *Nucleic Acids Research* 45 (D1): D446–56.

Nevers, Yannis, Megana K. Prasad, Laetitia Poidevin, Kirsley Chennen, Alexis Allot, Arnaud Kress, Raymond Ripp, et al. 2017. "Insights into Ciliary Genes and Evolution from Multi-Level Phylogenetic Profiling." *Molecular Biology and Evolution* 34 (8): 2016–34.

Niklas, Karl J., Edward D. Cobb, and Ulrich Kutschera. 2014. "Did Meiosis Evolve before Sex and the Evolution of Eukaryotic Life Cycles?" *BioEssays: News and Reviews in Molecular, Cellular and Developmental Biology* 36 (11): 1091–1101.

Ning, Jue, Thomas D. Otto, Claudia Pfander, Frank Schwach, Mathieu Brochet, Ellen Bushell, David Goulding, et al. 2013. "Comparative Genomics in Chlamydomonas and Plasmodium Identifies an Ancient Nuclear Envelope Protein Family Essential for Sexual Reproduction in Protists, Fungi, Plants,

Formatted: German (Germany)
Formatted: German (Germany)
Field Code Changed
Formatted: German (Germany)
Field Code Changed
Formatted: German (Germany)

and Vertebrates." *Genes & Development* 27 (10): 1198–1215.

Niu, Yulong, Chengcheng Liu, Shayan Moghimyfiroozabad, Yi Yang, and Kambiz N. Alavian. 2017. "PrePhyloPro: Phylogenetic Profile-Based Prediction of Whole Proteome Linkages." *PeerJ* 5 (August): e3712.

Pedregosa, Fabian, Gaël Varoquaux, Alexandre Gramfort, Vincent Michel, Bertrand Thirion, Olivier Grisel, Mathieu Blondel, et al. 2011. "Scikit-Learn: Machine Learning in Python." *Journal of Machine Learning Research: JMLR* 12 (Oct): 2825–30.

Pellegrini, M., E. M. Marcotte, M. J. Thompson, D. Eisenberg, and T. O. Yeates. 1999. "Assigning Protein Functions by Comparative Genome Analysis: Protein Phylogenetic Profiles." *Proceedings of the National Academy of Sciences of the United States of America* 96 (8): 4285–88.

Rajavel, M., B. Philip, B. M. Buehrer, B. Errede, and D. E. Levin. 1999. "Mid2 Is a Putative Sensor for Cell Integrity Signaling in Saccharomyces Cerevisiae." *Molecular and Cellular Biology* 19 (6): 3969–76.

Ranea, Juan A. G., Corin Yeats, Alastair Grant, and Christine A. Orengo. 2007. "Predicting Protein Function with Hierarchical Phylogenetic Profiles: The Gene3D Phylo-Tuner Method Applied to Eukaryotic Genomes." *PLoS Computational Biology* 3 (11): e237.

Ruano-Rubio, Valentín, Olivier Poch, and Julie D. Thompson. 2009. "Comparison of Eukaryotic Phylogenetic Profiling Approaches Using Species Tree Aware Methods." *BMC Bioinformatics* 10 (November): 383.

Sayers, Eric W., Tanya Barrett, Dennis a. Benson, Evan Bolton, Stephen H. Bryant, Kathi Canese, Vyacheslav Chetvernin, et al. 2010. "Database Resources of the National Center for Biotechnology Information." *Nucleic Acids Research* 38 (Database issue): D5–16.

Schmitt, Thomas, David N. Messina, Fabian Schreiber, and Erik L. L. Sonnhammer. 2011. "Letter to the Editor: SeqXML and OrthoXML: Standards for Sequence and Orthology Information." *Briefings in Bioinformatics* 12 (5): 485–88.

Schurko, Andrew M., and John M. Logsdon Jr. 2008. "Using a Meiosis Detection Toolkit to Investigate Ancient Asexual 'Scandals' and the Evolution of Sex." *BioEssays: News and Reviews in Molecular, Cellular and Developmental Biology* 30 (6): 579–89.

Serber, Daniel W., John S. Runge, Debashish U. Menon, and Terry Magnuson. 2016. "The Mouse INO80 Chromatin-Remodeling Complex Is an Essential Meiotic Factor for Spermatogenesis." *Biology of Reproduction* 94 (1): 8.

Sherill-Rofe, Dana, Dolev Rahat, Steven Findlay, Anna Mellul, Irene Guberman, Maya Braun, Idit Bloch, et al. 2019. "Mapping Global and Local Coevolution across 600 Species to Identify Novel Homologous Recombination Repair Genes." *Genome Research*. https://doi.org/10.1101/gr.241414.118.

Skunca, Nives, Adrian Altenhoff, and Christophe Dessimoz. 2012. "Quality of Computationally Inferred Gene Ontology Annotations." *PLoS Computational Biology* 8 (5): e1002533.

Snitkin, Evan S., Adam M. Gustafson, Joseph Mellor, Jie Wu, and Charles DeLisi. 2006. "10.1186/1471-2105-7-420." *BMC Bioinformatics*. https://doi.org/10.1186/1471-2105-7-420.

Speijer, Dave, Julius Lukeš, and Marek Eliáš. 2015. "Sex Is a Ubiquitous, Ancient, and Inherent Attribute of

Formatted: French (France)

Field Code Changed

Field Code Changed

Formatted: German (Germany)

Formatted: Footer

Eukaryotic Life." *Proceedings of the National Academy of Sciences of the United States of America* 112 (29): 8827–34.

Strzalka, Wojciech, and Alicja Ziemienowicz. 2011. "Proliferating Cell Nuclear Antigen (PCNA): A Key Factor in DNA Replication and Cell Cycle Regulation." *Annals of Botany* 107 (7): 1127–40.

Szklarczyk, Damian, John H. Morris, Helen Cook, Michael Kuhn, Stefan Wyder, Milan Simonovic, Alberto Santos, et al. 2017. "The STRING Database in 2017: Quality-Controlled Protein-Protein Association Networks, Made Broadly Accessible." *Nucleic Acids Research* 45 (D1): D362–68.

Tabach, Yuval, Allison C. Billi, Gabriel D. Hayes, Martin A. Newman, Or Zuk, Harrison Gabel, Ravi Kamath, et al. 2013. "Identification of Small RNA Pathway Genes Using Patterns of Phylogenetic Conservation and Divergence." *Nature* 493 (7434): 694–98.

Ta, Hung Xuan, Patrik Koskinen, and Liisa Holm. 2011. "A Novel Method for Assigning Functional Linkages to Proteins Using Enhanced Phylogenetic Trees." *Bioinformatics* 27 (5): 700–706.

"TAIR - Portals - Genome Snapshot." n.d. Accessed February 19, 2020. https://www.arabidopsis.org/portals/genAnnotation/genome_snapshot.jsp.

Thauvin-Robinet, Christel, Jaclyn S. Lee, Estelle Lopez, Vicente Herranz-Pérez, Toshinobu Shida, Brunella Franco, Laurence Jego, et al. 2014. "The Oral-Facial-Digital Syndrome Gene C2CD3 Encodes a Positive Regulator of Centriole Elongation." *Nature Genetics* 46 (8): 905–11.

Train, Clément-Marie, Miguel Pignatelli, Adrian Altenhoff, and Christophe Dessimoz. 2018. "iHam & pyHam: Visualizing and Processing Hierarchical Orthologous Groups." *Bioinformatics* , December. https://doi.org/10.1093/bioinformatics/bty994.

Valansi, Clari, David Moi, Evgenia Leikina, Elena Matveev, Martín Graña, Leonid V. Chernomordik, Héctor Romero, Pablo S. Aguilar, and Benjamin Podbilewicz. 2017. "Arabidopsis HAP2/GCS1 Is a Gamete Fusion Protein Homologous to Somatic and Viral Fusogens." *The Journal of Cell Biology.*

Wang, Xiu-Tang, Can Yuan, Ting-Ting Yuan, and Su-Juan Cui. 2012. "The Arabidopsis LFR Gene Is Required for the Formation of Anther Cell Layers and Normal Expression of Key Regulatory Genes." *Molecular Plant* 5 (5): 993–1000.

Weidemann, Marina, Karin Schuster-Gossler, Michael Stauber, Christoph Wrede, Jan Hegermann, Tim Ott, Karsten Boldt, et al. 2016. "CFAP157 Is a Murine Downstream Effector of FOXJ1 That Is Specifically Required for Flagellum Morphogenesis and Sperm Motility." *Development* 143 (24): 4736–48.

Werven, Folkert J. van, Gregor Neuert, Natalie Hendrick, Aurélie Lardenois, Stephen Buratowski, Alexander van Oudenaarden, Michael Primig, and Angelika Amon. 2012. "Transcription of Two Long Noncoding RNAs Mediates Mating-Type Control of Gametogenesis in Budding Yeast." *Cell* 150 (6): 1170–81.

Wijeratne, Asela J., Changbin Chen, Wei Zhang, Ljudmilla Timofejeva, and Hong Ma. 2006. "The Arabidopsis Thaliana PARTING DANCERS Gene Encoding a Novel Protein Is Required for Normal Meiotic Homologous Recombination." *Molecular Biology of the Cell* 17 (3): 1331–43.

Zdobnov, Evgeny M., Fredrik Tegenfeldt, Dmitry Kuznetsov, Robert M. Waterhouse, Felipe A. Simão, Panagiotis Ioannidis, Mathieu Seppey, Alexis Loetscher, and Evgenia V. Kriventseva. 2017. "OrthoDB

**Field Code Changed**

**Formatted:** French (France)

**Formatted:** German (Germany)

**Field Code Changed**

v9.1: Cataloging Evolutionary and Functional Annotations for Animal, Fungal, Plant, Archaeal, Bacterial and Viral Orthologs." *Nucleic Acids Research* 45 (D1): D744–49.

Zelkowski, Mateusz, Katarzyna Zelkowska, Udo Conrad, Susann Hesse, Inna Lermontova, Marek Marzec, Armin Meister, Andreas Houben, and Veit Schubert. 2019. "Arabidopsis NSE4 Proteins Act in Somatic Nuclei and Meiosis to Ensure Plant Viability and Fertility." *Frontiers in Plant Science* 10 (June): 774.

Zerbino, Daniel R., Premanand Achuthan, Wasiu Akanni, M. Ridwan Amode, Daniel Barrell, Jyothish Bhai, Konstantinos Billis, et al. 2018. "Ensembl 2018." *Nucleic Acids Research* 46 (D1): D754–61.

**Formatted:** Footer

Figure 2

Click here to access/download;Figure;2-ROC_curves.eps

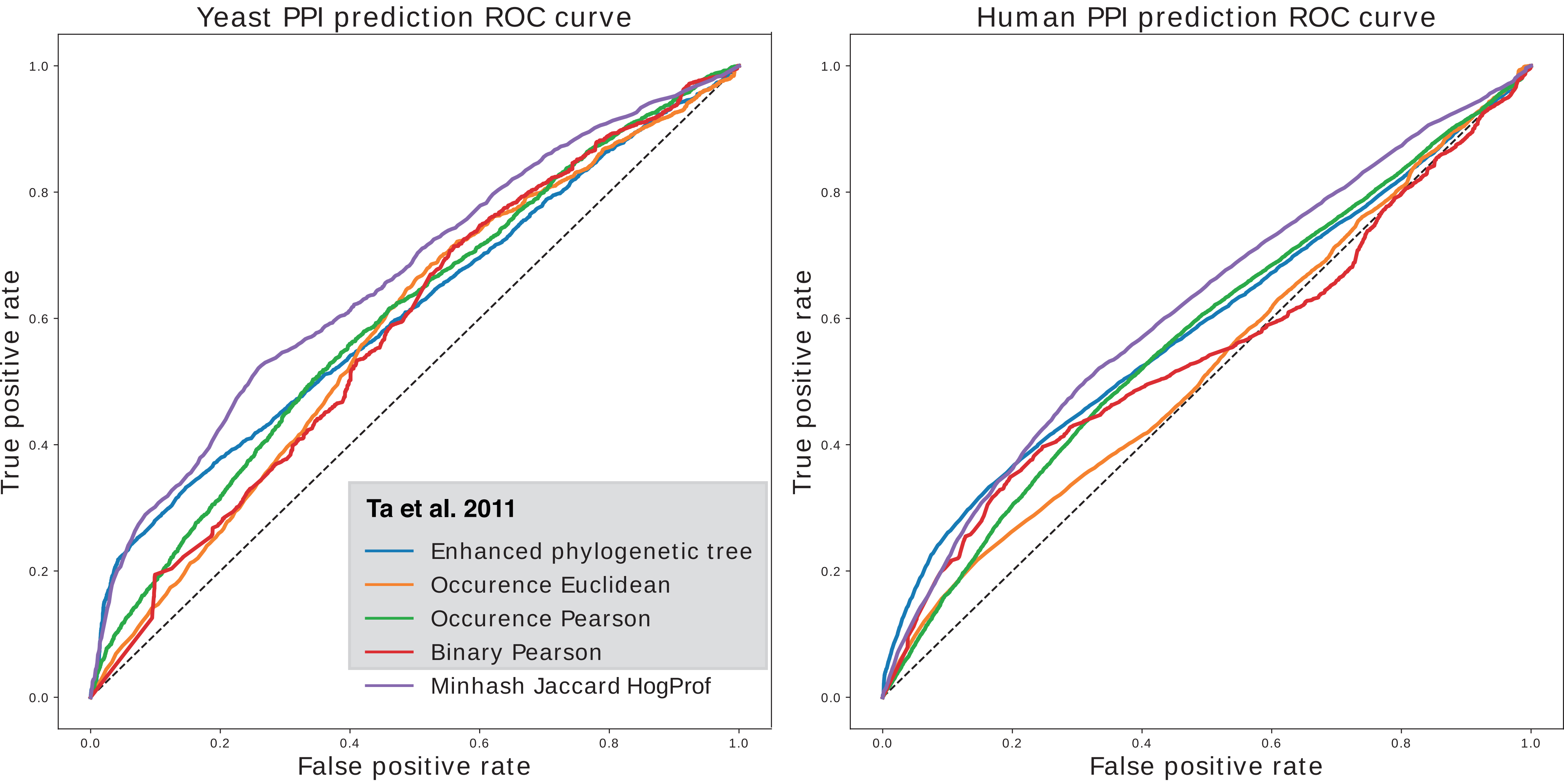

Figure 3

Click here to access/download;Figure;3-kineto_hash.eps

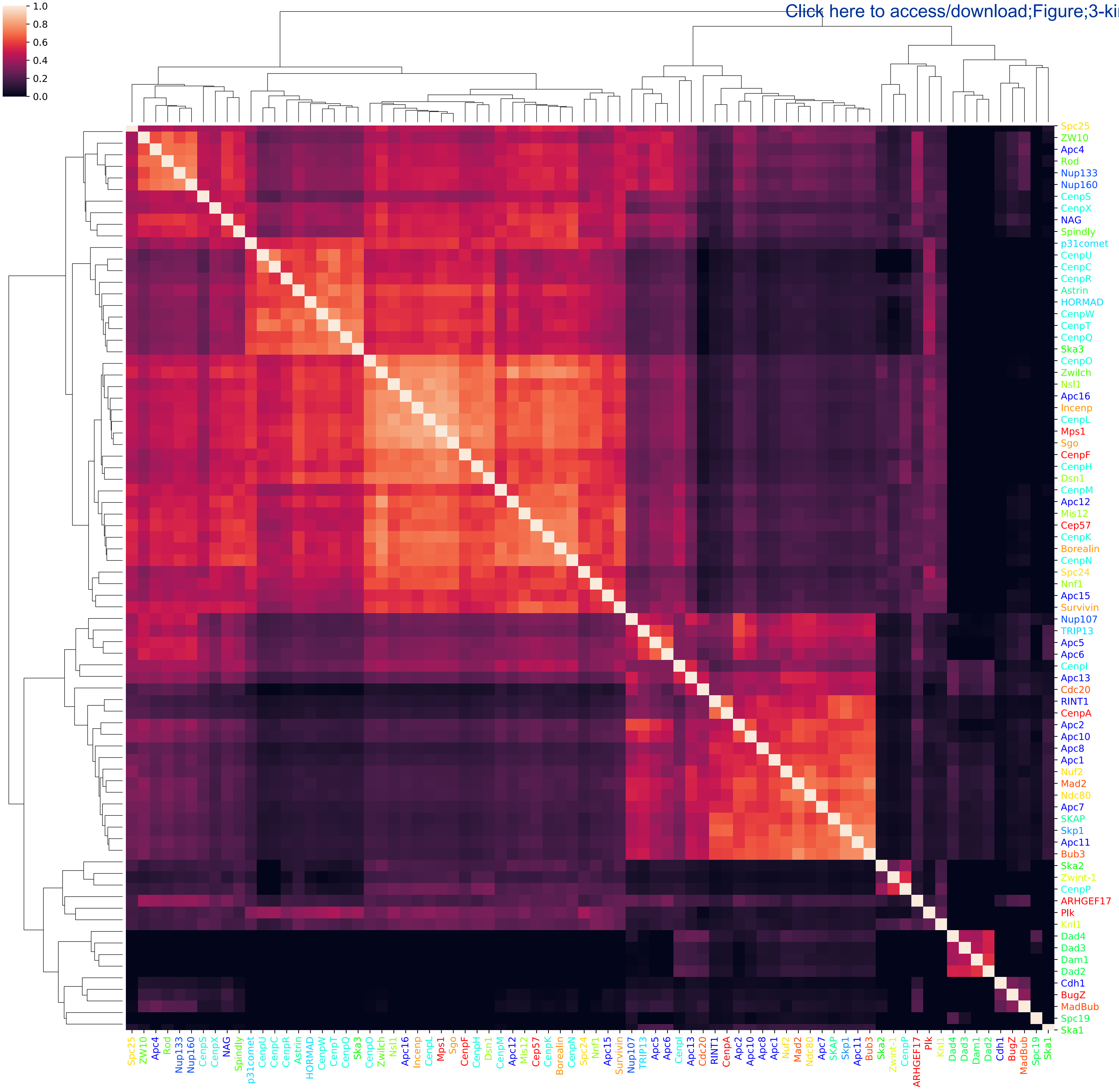

Figure 4

Click here to access/download;Figure;4-kineto_augment.eps

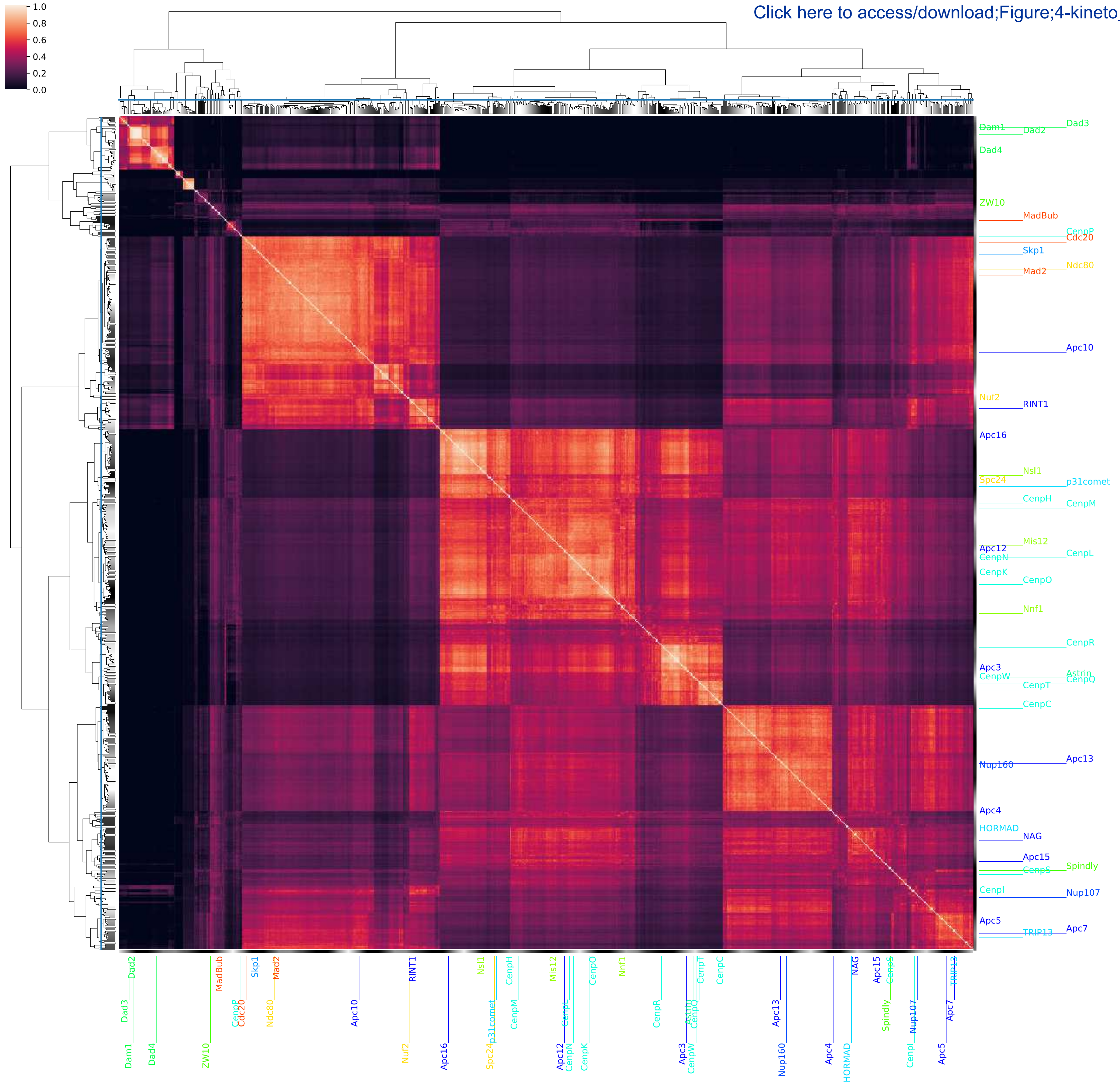

Figure 5

Click here to access/download;Figure;5-repro_hash_augment.eps

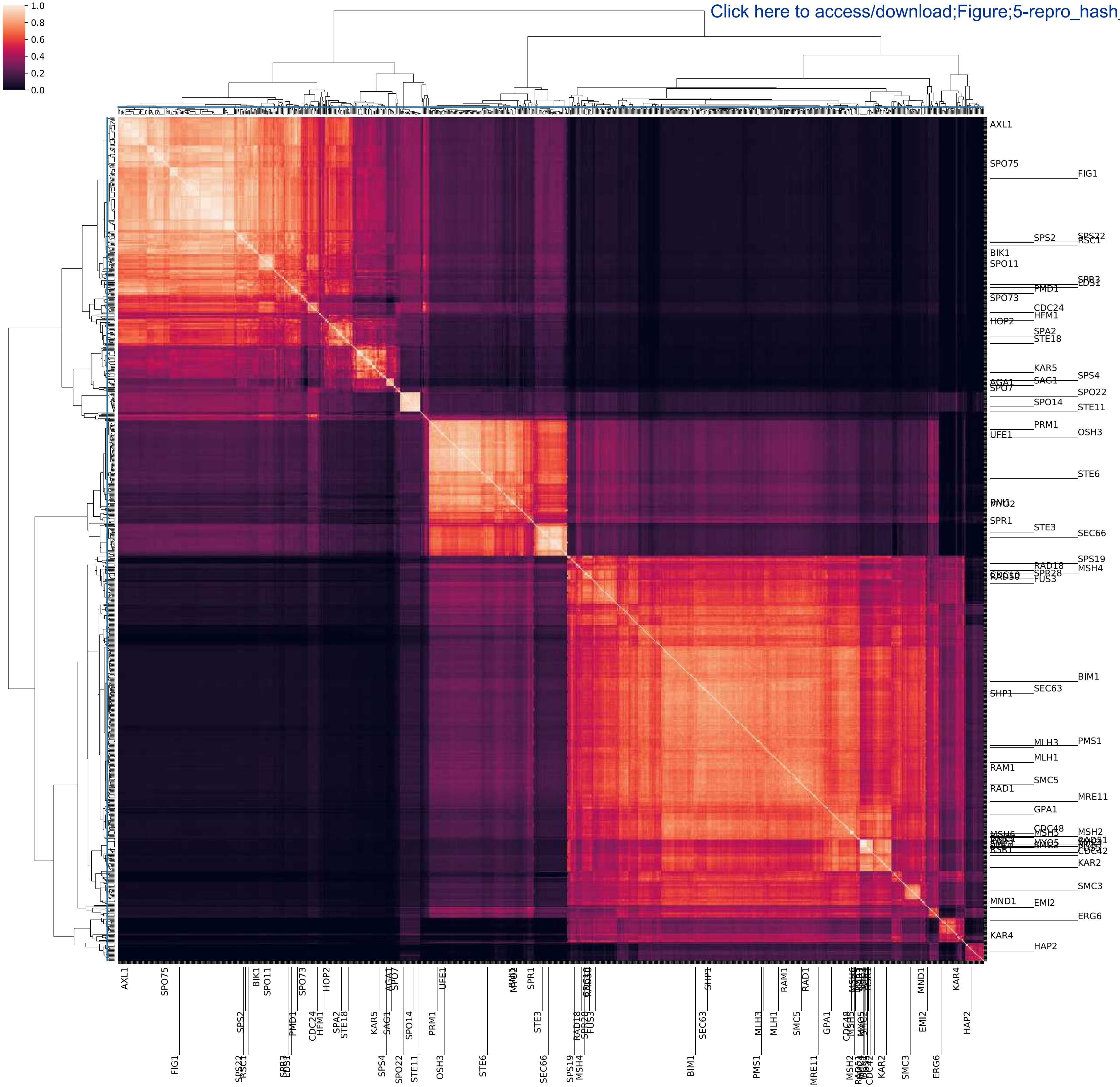

Figure 6

Click here to access/download;Figure;6-scaling.eps

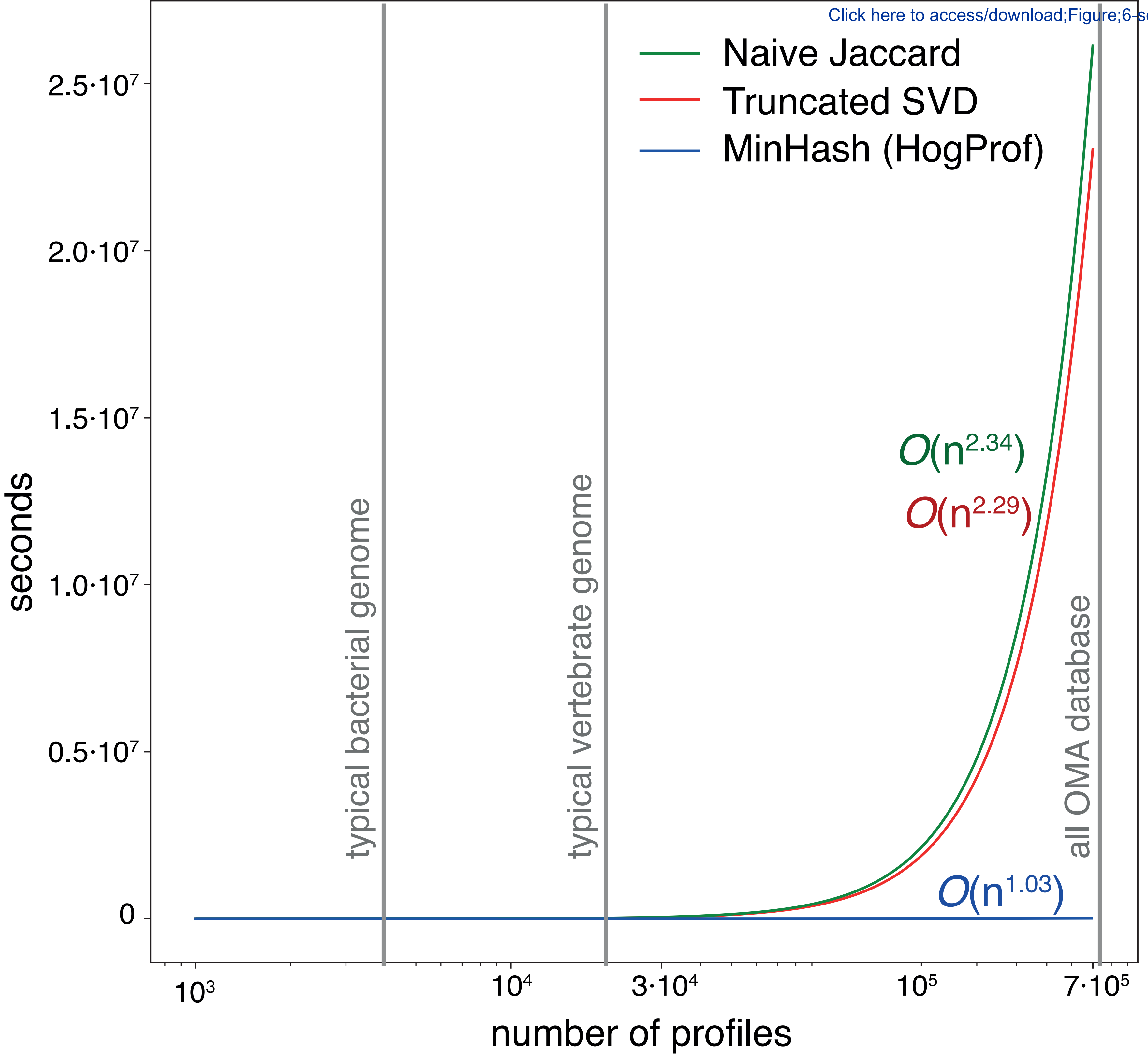

Figure 1

Click here to access/download;Figure;1-conceptual.eps

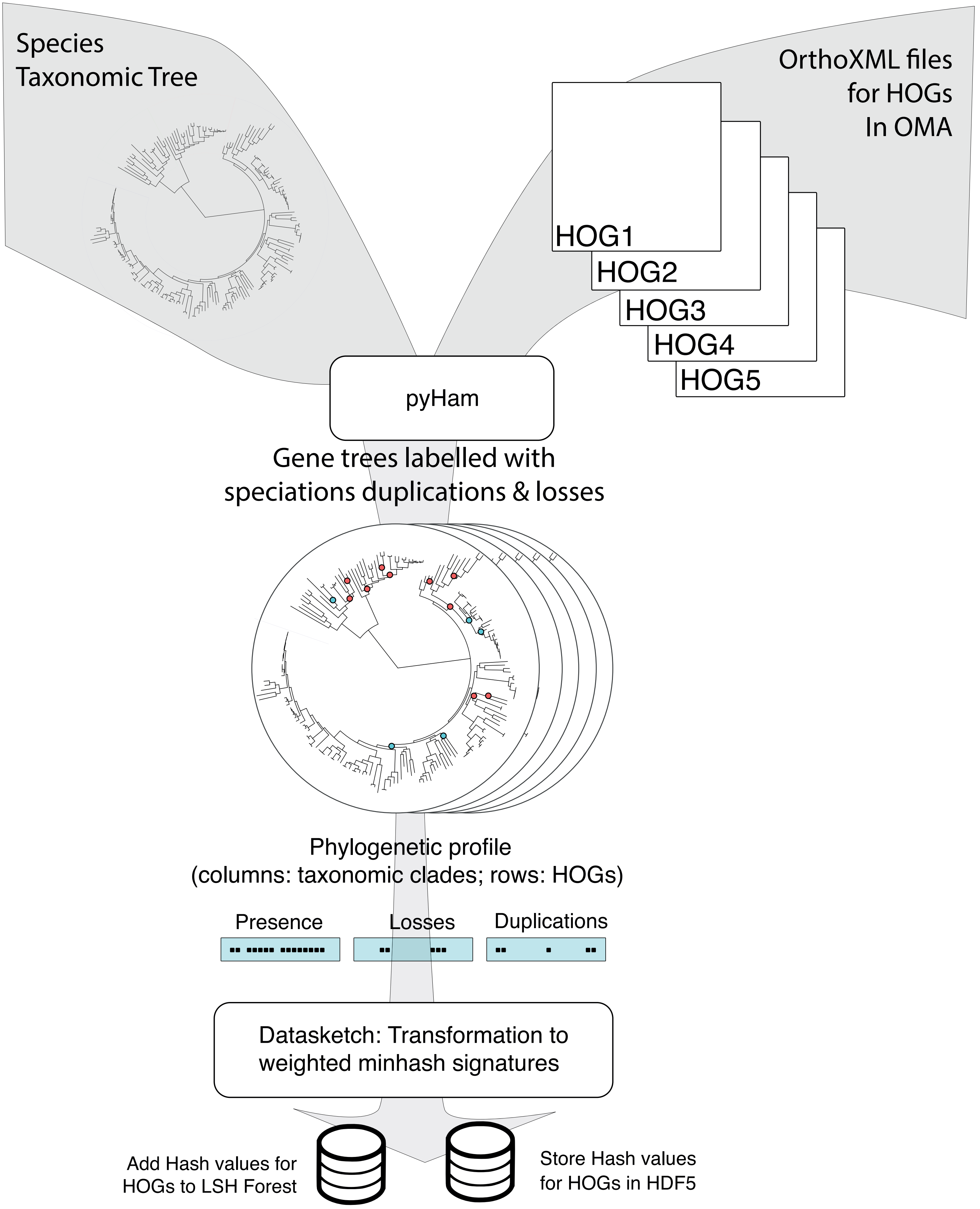

Click here to access/download
**Supporting Information**
Supplementary Data 1 -kineto_augment_goenrich.csv

Click here to access/download
**Supporting Information**
Supplementary Data 2 - hap_enrich.csv

Click here to access/download
**Supporting Information**
Supplementary Data 3 - gex_enrich.csv

Click here to access/download
**Supporting Information**
Supplementary Data 4- repro_hogs.csv

Click here to access/download
**Supporting Information**
Supplementary Data 5 -repro_augment_goenrich.csv
